# Attacking COVID-19 Progression Using Multi-Drug Therapy for Synergetic Target Engagement

**DOI:** 10.3390/biom11060787

**Published:** 2021-05-23

**Authors:** Mathew A. Coban, Juliet Morrison, Sushila Maharjan, David Hyram Hernandez Medina, Wanlu Li, Yu Shrike Zhang, William D. Freeman, Evette S. Radisky, Karine G. Le Roch, Carla M. Weisend, Hideki Ebihara, Thomas R. Caulfield

**Affiliations:** 1 Department of Cancer Biology, Mayo Clinic, 4500 San Pablo Road South, Jacksonville, FL 32224, USA; Coban.Mathew@mayo.edu (M.A.C.); radisky.evette@mayo.edu (E.S.R.); 2 Department of Microbiology and Plant Pathology, University of California, 900 University, Riverside, CA 92521, USA; jmorriso@ucr.edu; 3 Division of Engineering in Medicine, Department of Medicine, Brigham and Women’s Hospital, Harvard Medical School, 65 Landsdowne St, Cambridge, MA 02139, USA; SMAHARJAN@bwh.harvard.edu (S.M.); A01207537@itesm.mx (D.H.H.M.); wli46@bwh.harvard.edu (W.L.); yszhang@research.bwh.harvard.edu (Y.S.Z.); 4 Department of Neurology, Mayo Clinic, 4500 San Pablo South, Jacksonville, FL 32224, USA; freeman.william1@mayo.edu; 5 Department of Molecular, Cell and Systems Biology, University of California, 900 University, Riverside, CA 92521, USA; karine.leroch@ucr.edu; 6 Department of Molecular Medicine, Mayo Clinic, Rochester, MN 55905, USA; Weisend.Carla@mayo.edu (C.M.W.); Ebihara.Hideki@mayo.edu (H.E.); 7 Department of Neuroscience, Mayo Clinic, Jacksonville, FL 32224, USA; 8 Department of Quantitative Health Science, Division of Computational Biology, Mayo Clinic, Jacksonville, FL 32224, USA; 9 Department of Clinical Genomics, Mayo Clinic, Rochester, MN 55905, USA; 10 Department of Biochemistry & Molecular Biology, Mayo Clinic, Rochester, MN 55905, USA

**Keywords:** COVID, drug discovery, multi-drug therapy, bioprinting

## Abstract

COVID-19 is a devastating respiratory and inflammatory illness caused by a new coronavirus that is rapidly spreading throughout the human population. Over the past 12 months, severe acute respiratory syndrome coronavirus 2 (SARS-CoV-2), the virus responsible for COVID-19, has already infected over 160 million (>20% located in United States) and killed more than 3.3 million people around the world (>20% deaths in USA). As we face one of the most challenging times in our recent history, there is an urgent need to identify drug candidates that can attack SARS-CoV-2 on multiple fronts. We have therefore initiated a computational dynamics drug pipeline using molecular modeling, structure simulation, docking and machine learning models to predict the inhibitory activity of several million compounds against two essential SARS-CoV-2 viral proteins and their host protein interactors—S/Ace2, Tmprss2, Cathepsins L and K, and Mpro—to prevent binding, membrane fusion and replication of the virus, respectively. All together, we generated an ensemble of structural conformations that increase high-quality docking outcomes to screen over >6 million compounds including all FDA-approved drugs, drugs under clinical trial (>3000) and an additional >30 million selected chemotypes from fragment libraries. Our results yielded an initial set of 350 high-value compounds from both new and FDA-approved compounds that can now be tested experimentally in appropriate biological model systems. We anticipate that our results will initiate screening campaigns and accelerate the discovery of COVID-19 treatments.

## 1. Introduction

COVID-19 is a disease caused by severe acute respiratory syndrome coronavirus 2 (SARS-CoV-2). It was identified in Wuhan city, in the Hubei province of China in December 2019 [1,2,3]. The virus is spread between people via small droplets produced by talking and coughing. The disease was declared a global pandemic by the World Health Organization (WHO) on March 11th, 2020. While a large proportion of the cases results in mild symptoms such as fever, cough, fatigues, loss of smell and taste, as well as shortness of breath, some cases progress into more acute respiratory symptoms such as pneumonia, multi-organ failure, septic shock and blood clots. These more severe symptoms can lead to death and are likely to be precipitated by a cytokine storm after infection and multiplication of the virus in humans. Indeed, data indicate that the levels of IL-6 correlate with respiratory and organ failures [4]. So far, the estimated death rate from SARS-CoV-2 is above 1.3%, which is more than 10-fold higher than the death rate from seasonal influenza [5]. Older patients and patients who have serious underlying medical conditions such as hypertension, diabetes, and asthma are at higher risk for severe disease outcomes [6]. A clear understanding of the genetics and molecular mechanisms controlling severe illness remains to be determined.

**Figure 1:**
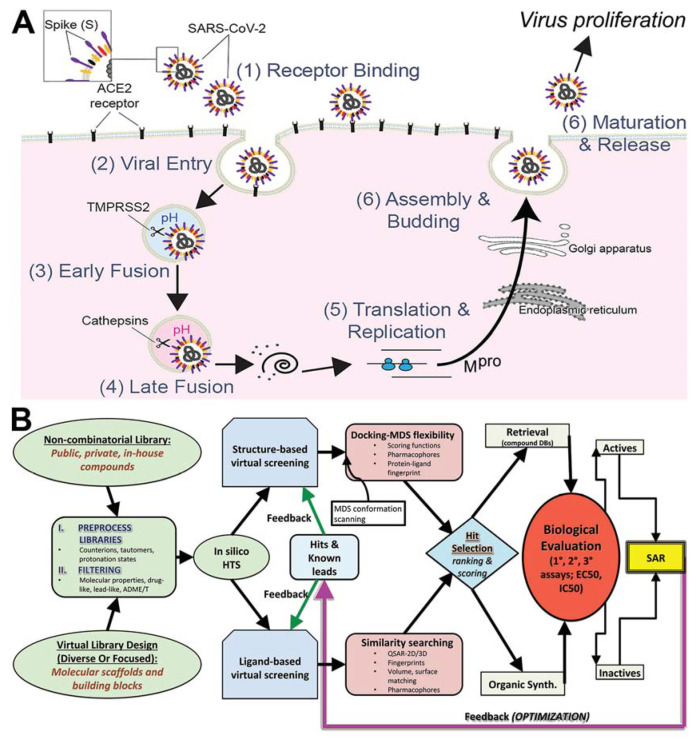
Flowchart for drug pipeline for attacking COVID-19 via a polypharma small-molecule approach using in silico screening and advanced simulation biasing. (**A**) Biological infection of SARS-CoV-2 from initial binding, entry and replication to virus proliferation. (**B**) Overview of COVID-19 Drug Discovery Pipeline.

SARS-CoV-2 is a positive-sense, single-stranded RNA betacoronavirus, closely related to SARS-CoV, which caused severe acute respiratory syndrome (SARS) in 2003, and Middle East respiratory syndrome coronavirus (MERS-CoV), which caused MERS in 2012. Positive-strand RNA viruses are a large fraction of known viruses including common pathogens such as rhinoviruses that cause common colds, as well as dengue virus, hepatitis C virus (HCV), and West Nile virus. The first genome sequence of SARS-CoV-2 was released in early January 2020 on the open-access virological website (http://virological.org/ (accessed on 22 May 2021)) [7]. Its genome is ~29.8 kb and possesses 14 open reading frames (ORFs), encoding 27 proteins [8]. The genome contains four structural proteins: spike (S) glycoprotein, envelope (E) protein, membrane (M) protein, and nucleocapsid (N) protein. The E and M proteins form the viral envelope, while the N protein binds to the virus’s RNA genome. The spike glycoprotein is a key surface protein that interacts with cell surface receptor, angiotensin-converting enzyme 2 (ACE2), mediating entrance of the virus into host cells [9]. In addition to its dependence on the binding of S to ACE2, cell entry also requires priming of S by the host serine protease, transmembrane serine protease 2 (TMPRSS2). TMPRSS2 proteolytically processes S, promoting membrane fusion, cell invasion and viral uptake [10,11]. Blocking viral entry by targeting S/ACE2 interaction or TMPRSS2-mediated priming may constitute an effective treatment strategy for COVID-19. The non-structural proteins, which include the main viral protease (nsp5 or M^pro^) and RNA polymerase (nsp12), regulate virus replication and assembly. They are expressed as two long polypeptides, pp1a and pp1ab, which are proteolytically processed by M^pro^. The key role of M^pro^ in viral replication makes it a good therapeutic target as well. A third group of proteins are described as accessory proteins. This group is the least understood, but its members are thought to counteract host innate immunity (Figure 1A) [12].

Until February 2021, there was no treatment or vaccine available to prevent or treat COVID-19 [13,14]. While the FDA granted emergency use authorization (EUA) for the 65-year-old antimalarial drug, hydroxychloroquine, COVID-19 treatment based on early results from clinical trial in China and France [15,16,17,18], results from larger cohorts reported that hydroxychloroquine did not decrease viral replication, pneumonia or hospital mortality, and may in fact increase cardiac arrest in patients infected with COVID-19 [19,20]. In another study published in the New England Journal of Medicine, the antiviral remdesivir, a drug that was originally developed to fight Ebola, seemed to improve patients with severe breathing problems [21] and has also recently been granted EUA by the FDA. Repurposing drugs that are designed to treat other diseases is one of the quickest ways to find therapeutics to control the current pandemic. Such drugs have already been tested for toxicity issues and can be granted EUA by the FDA to help doctors treat COVID-19 patients. More recently, the USDA issued an EUA for bamlanivimab and etesevimab, two monoclonal antibodies directed against the Spike protein of SAR-CoV-2 that block the virus entry into human cells. This EUA was issued for the treatment of mild to moderate COVID-19 in adults and pediatric patients of 12 years old or older. A randomized, double-blind, placebo-controlled clinical trial demonstrated that hospitalization or death was reduced by 70% when both antibodies were administrated together in non-hospitalized adults with mild COVID-19 symptoms [22]. While the search for drugs efficient against COVID-19 has been so far limited, several companies have developed successful vaccine strategies. The most successful vaccines to date have been developed by Moderna, Pfizer/BioNTech and Johnson & Johnson. Both Moderna and the Pfizer/BioNTech vaccines are designed novel mRNA and lipid nanoparticle (LNP)-based vaccines that have been approved for adult use in the U.S. in late 2020 [23,24,25].

In addition to vaccine strategies, one of the most efficient ways to attack a virus is to use drug cocktails to target multiple enzymes/pathways used by the virus. Combination therapy has the advantage of being less likely to select for treatment-resistant viral mutants. Such a strategy has been successfully used to treat HCV and human-immunodeficiency virus (HIV) infections. In the case of HCV, the treatment, Epclusa, combines sofosbuvir, which inhibits the viral RNA-dependent RNA polymerase (NS5B), and velpatasvir, a defective substrate that inhibits NS5A. Antiretroviral therapy (ART) against HIV combines drugs from different drug classes to target disparate aspects of the HIV replication cycle. These drug classes include nucleoside reverse transcriptase inhibitors, non-nucleoside reverse transcriptase inhibitors, protease inhibitors, fusion inhibitors, CCR5 antagonists, post-attachment inhibitors, and integrase inhibitors. One example from the HIV-AIDS literature is the randomized comparison of four groups of patients comparing monotherapy to combination therapies: zidovudine (ZDV) monotherapy; ZDV zidovudine and didanosine; ZDV plus zalcitabine; or didanosine monotherapy. This randomized trial showed positive results when ZDT was combined with didanosine or zalcitabine, and for didanosine compared to ZDT monotherapy in raising CD4 counts greater than 50% [26]. Combination therapy has become standard of care initial treatment in other infectious diseases such as tuberculosis, and failure to cure with monotherapy requires multi-drug therapy (MDT) [27]. Similar MDT is also found effective in HCV infection using glecaprevir and pibrentasvir combination therapies which lead to sustained virological response rates as far out as 12 weeks post-treatment [28].

Here, we propose a combination small-molecule therapy for COVID-19 (antivirals). We aim to target the SARS-CoV-2 replication cycle at multiple levels to synergistically inhibit viral spread and dissemination. Using a computational pipeline that aimed to expeditiously identify lead compounds against COVID-19, we combined compound library preparation, molecular modeling, and structure simulations to generate an ensemble of conformations and increase high-quality docking outcomes against two essential SARS-CoV-2 viral proteins and their host protein interactions; S/ACE2, Tmprss2, Cathepsin L and K, and M^pro^ that are known to control both viral binding or entry and virus replication (Figure 1A). Our approach (Figure 1B) will most likely lead into experimental virus inhibition screening, structural characterization of binding interactions by X-ray crystallography, and compound safety profiling. Virtual screening (VS) is a rational driven controller for identification of new hits from compound libraries [29] using either ligand-based (LBvs) or structure-based (SBvs) virtual screening [30]. LBvs tactics use structural and biological data of known active compounds to select favorable candidates with biological activity from experiments [31,32]. SBvs approaches, on the other hand, examine quantitative structure–activity relationships (QSAR), clustering, pharmacophore and 3D shape matching [33]. The utility of VS is evident in the growth of our knowledge base of new compounds and existing drugs as well as the expansion of our structural databases. SBvs is generally the preferred approach when access to the target 3D information derived from NMR, X-ray crystallography or homology models [31,32] is possible. Molecular docking (docking) is the most common SBvs approach used today [34,35,36,37,38,39] and searches for the ideal position and orientation (called “pose”) of the small molecule within a target’s binding site, which gives a score for the pose. When including knowledge of experimentally known compounds (“actives”) from a 3D target, LBvs and SBvs can be combined to increase the likelihood of obtaining new actives from searches [40].

Hit identification in VS also requires careful selection of the methods used based on the goal of the project (e.g., compound databases and libraries can be either proprietary, commercial or public) [41]. ZINC is one such large public database often used in VS [42] that contains millions of compounds. By contrast, other libraries have structure–activity relationship (SAR) databases [43] that integrate information about compound interactions with their known targets. DrugBank and Chem-Space are other attractive sources of compounds for drug repurposing (or repositioning) [44,45,46,47], and contain drug diversity that is useful for scaffold development [48,49].

Advances in computing power have increased the utility of in silico screening capabilities and balanced the need for accuracy with virtual high-throughput screening approximations and assumptions [50,51,52,53], while recent techniques have improved accuracy without sacrificing CPU time [54,55,56,57,58,59] (Figure 1B). Further innovations in docking methods have improved the exactness of empirical docking equations [34,35,38,39,40,60]. Accuracy is improved by incorporating molecular flexibility with simulations [57,61,62,63,64,65], thus capturing conformational information on structural changes that directly impact compound docking results.

Here, we present in silico screening of both the approved FDA compound library and >30 million compounds representing new chemical entities (NCEs) [66,67,68,69]. Other libraries consisting of approved drugs, natural products, and a subset of the ZINC database were also included based on their relationship with SARS-CoV-2 [42,70,71,72]. Our findings include >350 compounds, including both NCEs (310) and FDA repurposing compounds (40). Our approach combines VS and careful library selection with advanced docking techniques to efficiently search the behemoth chemical landscape of possible organic compounds [73] and identify high-value hits against key SARS-CoV-2 targets.

## 2. Materials and Methods

### 2.1. Structural Modeling of ACE2–S protein

For ACE2–S protein, PDB code 6VW1 was used to construct the model [74]. While the structure was mostly complete, chain F (S protein) was missing more residues, though it had residue Ala522. Chain E (S protein) was only missing residue 522. Residue Ala522 was built into chain E using COOT and the extraneous molecules (solvent/cryoprotectant) and chains were deleted to leave only the heterodimer ACE2–S protein. The entire full-length structure was modeled, filling in any gaps or unresolved portions from the X-ray crystallography experimental structure. This process prepared the structure to be used for computational studies, not to generate a de novo model. All information about the protein was found on the corresponding Uniprot page. The PDBePISA server was used to data mine the interface between ACE2 and S protein [75].

### 2.2. Structural Modeling of TMPRSS2

A homology model was constructed on the basis of a prothrombin crystal structure in complex with the ligand analog (PDB code 3F68) [76]. We modeled the 492 amino acid TMPRSS2 protein in two different ways: YASARA based and SwissModel server based [77,78,79]. First, the YASARA-based model begins with the FASTA sequence: 

MALNSGSPPAIGPYYENHGYQPENPYPAQPTVVPTVYEVHPAQYYPSPVPQYAPRVLTQASNPVVCTQPKSPSGTVCTSKTKKALCITLTLGTFLVGAALAAGLLWKFMGSKCSNSGIECDSSGTCINPSNWCDGVSHCPGGEDENRCVRLYGPNFILQVYSSQRKSWHPVCQDDWNENYGRAACRDMGYKNNFYSSQGIVDDSGSTSFMKLNTSAGNVDIYKKLYHSDACSSKAVVSLRCIACGVNLNSSRQSRIVGGESALPGAWPWQVSLHVQNVHVCGGSIITPEWIVTAAHCVEKPLNNPWHWTAFAGILRQSFMFYGAGYQVEKVISHPNYDSKTKNNDIALMKLQKPLTFNDLVKPVCLPNPGMMLQPEQLCWISGWGATEEKGKTSEVLNAAKVLLIETQRCNSRYVYDNLITPAMICAGFLQGNVDSCQGDSGGPLVTSKNNIWWLIGDTSWGSGCAKAYRPGVYGNVMVFTDWIYRQMRADG. Topological domains have the following characteristics: residues 1–84 form the cytoplasmic sequence; residues 85–105 form the transmembrane domain region (helical 21 aa); and residues 106–492 form the signal-anchor for type II membrane protein (extracellular), where the protein has two main chains: a non-catalytic chain (Met1-Arg225) and a catalytic chain (Ile256-Gly492), where each domain is modeled as a separate unit built together in composite. Disulfide bonds exist between several residues (113 ↔ 126), (120 ↔ 139), (133 ↔ 148), (172 ↔ 231), (185 ↔ 241), (244 ↔ 365), (281 ↔ 297), (410 ↔ 426), (437 ↔ 465), which can be informative for building the structure. Glycosylation sites are also possible at residues N213 and N249. A cleavage site exists between Arg255 and Ile256 to produce the mature S1 peptidase domain. The second method, homology modeling, was performed using the SwissModel server (2020) after performing a BLAST search on available protein structures in the RCSB database. 

### 2.3. Structural Modeling of M^pro^

The structure for M^pro^ co-crystallized with tert-butyl (1-((*S*)-1-(((*S*)-4-(benzylamino)-3,4-dioxo-1-((*S*)-2-oxopyrrolidin-3-yl)butan-2-yl)amino)-3-cyclopropyl-1-oxopropan-2-yl)-2-oxo-1,2-dihydropyridin-3-yl)carbamate (also referred to as alpha-ketoamide 13b) PDB 6Y2F was used. This structure was also mostly complete. Residues E47 and D48 were built in COOT. To build the missing residues, the coordinates and structural factors were downloaded, 2mFo-DFc and FEM maps were generated, and real space refine zone/regularize zone was used to manually fit added residues to electron density as well as optimize local geometry. The ligand (alpha-ketoamide 13b) was left for usage as a cognate ligand for virtual screening.

### 2.4. Molecular Simulation Refinement of Protein Models

Molecular dynamics simulations (MDS) and Monte Carlo (MC) simulations were performed on the protein to allow local regional changes for all acids in each structure. The X-ray refinement for MC was built using the YASARA SSP/PSSM method [80,81,82,83,84,85]. The structure was relaxed to the YASARA/Amber force field using knowledge-based potentials within YASARA. The side chains and rotamers were adjusted with knowledge-based potentials, simulated annealing with explicit solvent, and small equilibration simulations using YASARA’s refinement protocol [86]. 

Refinement of the finalized models was completed using either Schrodinger’s LC-MOD MC-based module or NAMD2 protocols. These refinements started with YASARA-generated initial refinement of the proteins [80,81,82,84]. The superposition and subsequent refinement of each protein region yields a complete model. The final structures were subjected to energy optimization with Polak–Ribière conjugate gradient (PRCG) and an R-dependent dielectric. Atom consistency was checked for all amino acids of the full-length wild-type structure, verifying correctness of chain name, dihedrals, angles, torsions, non-bonded interactions, electrostatics, atom typing, and parameters. Models were exported to the following formats: Maestro (MAE), YASARA (PDB). Model manipulation was performed with Maestro (Macromodel, version 9.8, Schrodinger, LLC, New York, NY, 2010), or Visual Molecular Dynamics (VMD) [87].

MDS and MC searching were completed on each model for conformational sampling using methods previously described in the literature [57,58,64,65]. Briefly, each protein system was minimized with relaxed restraints using either Steepest Descent or PRCG, then allowed to undergo the MC search criteria, as shown in the literature [58,64,65,88]. The primary purpose of MC, in this scenario, is examining any conformational variability that may occur with each protein.

### 2.5. Production (Analytical) MDS Protocol

The total atomic force field was used to minimize the energy of the system, namely, the descent algorithm for 20,000 steps, with an iteration interval of 2 fs. Solvent equilibration was carried out using positional restrictions imposed on the atoms of protein structures, while the solvent molecules remained mobile for all 100 ps. Each system was placed in a box in which the layer of TIP3P water molecules extended 10 Å from the nearest protein atom. The final systems were neutralized by the addition of Na^+^ and Cl^−^ ions to a concentration of 150 mM. All simulations were performed under periodic boundary conditions using the V-Rescale Thermostat algorithm to maintain temperature (310 K) and the Parrinello–Rahman Barostat algorithm for constant pressure (1 bar) [89,90]. Long-range unrelated interactions were calculated using the Particle-Mesh-Ewald (PME) method [91]. All molecules were relaxed with a MDS of 100 ns. Ligand topologies were created using the antechamber module from the AmberTools18 package [92].

Calculations on MDS trajectories including RMSD, RMSF, and H bonds were performed using VMD and internal tools therein (RMSD trajectory tool and Tk Console). Prior to calculations, the backbone (CONCα) atoms of each frame of the trajectories were aligned to the first frame as a reference, to remove the effect of random rotation/translation. After alignment, the per residue average of RMSF or RMSD in Å per frame across the entire MDS trajectory is given. For the ACE2-ligand simulations, the number of hydrogen bonds between the protein and ligand were recorded for each frame, and the occupancy of each specific H bond was defined as the percentage of frames the bond is present. RMSD, RMSF, and H bond data were plotted in 2D format in Excel. The RMSF was also appended to the beta column of the PDB and heat-mapped to the structure using a custom Tcl/Tk script and PyMOL. All molecular graphics were generated in PyMOL [93]. 

### 2.6. Docking Methods

#### 2.6.1. Site Mapping and Preparation of Proteins

Prior to the docking with ACE2, TMPRSS2, and M^pro^, we had completed rigorous MDS and MC conformational searching for each model for additional conformational sampling using methods previously described in the literature [57,58,64]. The primary purpose of MC, in this scenario, is examining any conformational variability that may occur with different orientations in the region near to protein–protein interfaces.

We used SiteMapper (version 20-3) [94] to identify possible binding sites for docking affinity with the proteins ACE2, TMPRSS2, and M^pro^. We also used our novel MDS biasing technique algorithm, Maxwell’s demon MD, for searching within these sites for potential flexible zones that would have beneficial peptide interactions, which served as a reductive filter limiting the total number of possible sites screened on the proteins to those with adequately deep binding grooves [62,65] or interesting insertion sites (ACE2). For M^pro^, the alpha-ketoamide 13b present in 6Y2F was used as a cognate ligand. For TMPRSS2, the ligand in 3F68, our template for the rebuilt homology model version, served as a cognate ligand.

#### 2.6.2. Libraries Used

Compounds were derived from either a set of all FDA-approved and clinically tested compounds, bioactive set of compounds, or a large multi-million compound set from ZINC database. In all cases the libraries were prepared using LigPrep. The ZINC database was pruned using parameters for better drug-like profile and removal of reactive functional groups and poor chemoinformatics properties delivering a large set suitable for screening all targets across dynamic time points from MDS.

#### 2.6.3. Docking Parameters

Over three million compounds were docked to each site using the Glide XP docking program (version 20-3) [94]. Each compound was converted into a set of energy minimized three-dimensional shapes with the Ligprep module. Without protein preparation, it was used for the correct distribution of protonation and post-minimization in the Optimized Potentials for Liquid Simulations (OPLS) 3 force field within the Maestro program (version 20-3) [95]. In the case of assigning restrictions based on ligands (M^pro^, TMPRSS2), we tried to cover the most important and strongest interactions. In the case of ACE2, a set of constraints was generated in sufficient quantities to produce combinations of possible interactions. H bond and 1.8 Å radius positional constraints were generated in the Schrodinger Glide module’s mesh generation tool. Aromatic and hydrophobic features were represented with short SMARTS. A partial matching protocol for applying constraints was also used to improve process accuracy. A high-throughput screening protocol with regulated ligand flexibility was applied.

Conformations of compound orientations were created using our standard protocols [60,94,96]. The starting conformation of relaxed protein structures was first obtained by the method of PRCG energy minimization with the OPLS 2005 force field [97,98] for 5000 steps, or until the energy difference between subsequent structures was less than 0.001 kJ/mol-Å units. Our docking methodology has been described previously [34,99,100]. 

Briefly, compounds were docked within the Schrödinger software suite (version 20-3) [101] using a virtual screening workflow (VSW) [34,60,94,102,103]. Alternative docking methods were also employed, including in-house software techniques for top leads for SAR elucidation. The top seeded poses were ranked and unfavorable scoring poses were discarded. Top favorable scores from initial dockings yielded hundreds of poses; the top five poses were retained. Molecular interactions of the ligand–protein interfaces were used to help determine the optimal binding set, which included descriptors were used to obtain atomic energy terms like hydrogen bond interaction, electrostatic interaction, hydrophobic enclosure and π–π stacking interaction that result during the docking run. Molecular modeling for importing and refining the proteins was completed [95].

Examinations of structure stability were undertaken for all proteins investigated, ACE2–S protein, TMPRSS2, and M^pro^, respectively [57,58,65,104,105,106]. Object stability was used to determine whether any changes in structure were deleterious to function from immediate inspection, which the FoldX algorithm can provide, prior to docking studies. Thus, we examined the local residues around the docking site and determined an electrostatic calculation may be useful to explain the change in function. The molecular model for the full structure and its truncated form are given (Figure S1) using our state-of-the-art methods, which have been established [57,58,63,64,65,106,107,108,109,110,111,112].

Local residues within the 12Å cutoff near docking sites were analyzed (Figures S1 and S2). Any interactions requiring inducible fit, or threonine/serine hydroxyl rotation or other docking parameters (π-stacking/halogen-directionality) were also included. Mapping electrostatics was accomplished using the Poisson–Boltzmann calculation for solvation on all amino acids for each docked structure [57,58,65,104,105,106].

For ACE2, we also used MDS to find out how the Y41A mutation affects PPI inhibition. We performed MD for wild-type and mutated protein. The in silico mutation was performed using PyMOL’s (v2.3.2) built-in tools. GROMACS 2018 and amber99 force field were used to conduct MD and further analysis of the results [93,113,114,115,116,117]. Visual inspection of every 10 frames allowed us to determine structural deformation tendencies at specific locations on the protein surface. According to the literature and our own findings, we focused on the predicted binding sites. Then, each trajectory was analyzed via the built-in clustering tool based on the RMSD distribution. Three of the most stable conformations of the binding site were chosen for the docking studies. All poses from each docking study were evaluated based on the docking scores, interaction diagrams and solvent exposure. To make predictions regarding the binding method, we carried out additional MDS for the best poses of each dock to select the most promising compounds from the docking results. 

### 2.7. In Vitro Experimentation

#### 2.7.1. In Vitro Materials

Gelatin from porcine skin (type-A, 300 bloom), methacrylic anhydride, and Triton X-100 were purchased from Sigma-Aldrich (St. Louis, MO, USA). Dulbecco’s modified Eagle medium (DMEM), Dulbecco’s phosphate-buffered saline (DPBS), Opti-MEM I reduced serum medium (Opti-MEM), fetal bovine serum (FBS), goat serum, trypsin-ethylenediaminetetraacetic acid (trypsin-EDTA), antibiotic-antimycotic solution stabilized (Anti-Anti, 100X), 4′,6-diamidino-2-phenylindole (DAPI), formalin (10% *w*/*v*), Live/Dead^®^ Viability/Cytotoxicity Kit, Alexa Fluor^®^ 594-phalloidin, dialysis membrane (M_w_ cutoff: 12–14 kDa) were obtained from Thermo Fisher Scientific (Waltham, MA, USA). The CellTiter 96^®^ AQueous One Solution Cell Proliferation Assay solution was obtained from Promega (Madison, WI, USA). Mouse cytochrome P450 3A (CYP3A4/CYP3A5 monoclonal) antibody and Alexa Fluor^®^ 594-conjugated goat anti-rabbit secondary antibody were purchased from Abcam (Cambridge, MA, USA). Ruthenium visible light photocrosslinking kit was purchased from Advanced BioMatrix (San Diego, CA, USA). Glass coverslips of 8 mm in diameter were obtained from Thomas Scientific (Swedesboro, NJ, USA). All other chemicals used in this study were obtained from Sigma-Aldrich unless otherwise mentioned.

#### 2.7.2. Synthesis of Gelatin Methacryloyl (GelMA)

GelMA was synthesized as described by us previously [118,119]. Briefly, 10 g of type A gelatin from porcine skin was dissolved in 100 mL of DPBS at 60 °C and 8.0 mL of methacrylic anhydride was added dropwise to gelatin solution with continuous stirring. The reaction was carried out at 50 °C for 3 h and then quenched by a 5-fold dilution with warm DPBS (40 °C). The product obtained was dialyzed against warm distilled water for 7 days using a dialysis membrane (M_w_ cutoff: 12–14 kDa) to remove the unreacted methacrylic anhydride. The dialyzed solution was then lyophilized to obtain a white GelMA foam. The lyophilized GelMA was stored at room temperature until further use.

#### 2.7.3. Cells

The human liver-derived HepG2-C3A cells, obtained from American Type Culture Collection (Manassas, VA, USA), were cultured in DMEM supplemented with 10% (*v*/*v*) FBS and 1% (*v*/*v*) Anti-Anti. The culture medium was replaced every 3 days. Vero E6 cells (ATCC) were maintained in complete DMEM (Invitrogen, Carlsbad, CA, USA) supplemented with 10% heat-inactivated FBS (Invitrogen) and 1X Penicillin-Streptomycin (Corning, NY, USA). Unless otherwise noted, all incubations involving human cells were carried out at 37 °C and 5% CO_2_ in a 95% humidified cell incubator.

#### 2.7.4. 3D Bioprinting of the Human Hepatic Microtissue Model

The 10% (*w*/*v*) GelMA solution was prepared by dissolving lyophilized GelMA in DPBS at 50 °C. The photoinitiator solution, a mixture of tris(2,2-bipyridyl) dichlororuthenium(II) hexahydrate (Ru) and sodium persulfate (SPS), was added to 10% (*w*/*v*) GelMA solution at concentrations of 4 mM Ru and 40 mM SPS, respectively, to obtain the GelMA hydrogel precursor solution. The GelMA hydrogel precursor solution was sterilized by filtration through a sterile 0.22-μm syringe filter and kept at 37 °C in an incubator, protected from light until use.

Approximately 80% confluent HepG2-C3A cells were treated with trypsin-EDTA solution (1X) at 37 °C after washing with DPBS (1X) to detach the cells from the culture flask. HepG2-C3A cells were then collected into 15 mL falcon tubes, and centrifuged at 1000 rpm for 5 min at room temperature. The medium was discarded, and the cells were resuspended in fresh DMEM supplemented with 10% (*v*/*v*) FBS and 1% (*v*/*v*) Anti-Anti.

Immediately prior to bioprinting of the hepatic microtissue constructs, the HepG2-C3A cell suspension was mixed with the 10% (*w*/*v*) GelMA hydrogel precursor solution, at a 1:1 ratio, to obtain a bioink solution containing 5% (*w*/*v*) GelMA, 2 mM Ru/20 mM SPS, and 4 × 10^6^ cells mL^−1^.

3D bioprinting of hepatic microtissues was achieved with a custom-built digital light processing (DLP) 3D bioprinting system as illustrated in Figure S1A [120] For each bioprinting step, 20 μL of the bioink solution was administered by a pipette on a circular coverslip placed on the stage and then exposed to the visible light (400–450 nm) source, with the digital hepatic pattern input into the bioprinter, where bioprinting of the microtissue was completed in 20 s. Each glass coverslip with the bioprinted construct was transferred into a well of 24-well plates and washed three times with warm DPBS. DMEM supplemented with 10% (*v*/*v*) FBS and 1% (*v*/*v*) Anti-Anti was then added and incubated for downstream experimental analyses.

#### 2.7.5. Characterizations of the Bioprinted 3D Hepatic Microtissues

At day 7 of culture, the hepatic microtissue constructs were washed with DPBS, fixed with 10% formalin for 15 min and permeabilized with 0.1% (*v*/*v*) Triton X-100 in DPBS for 30 min at room temperature. The hepatic microtissue constructs were then blocked with 5% (*v*/*v*) goat serum in DPBS for 2 h at room temperature and incubated with Alexa Fluor^®^ 594-phalloidin or CYP3A4/CYP3A5 monoclonal antibody (1:200 dilution in blocking buffer) overnight at 4 °C. For microtissue constructs incubated with Alexa Fluor^®^ 594-phalloidin, after washing three times with DPBS, nuclei were counterstained with DAPI (1:1000) for 5 min at room temperature and the cells within the hepatic microtissue constructs were observed under a fluorescence microscope (Nikon, Japan). On the other hand, for the tissue constructs incubated with CYP3A4/CYP3A5 primary antibody, after washing three times with DPBS, the Alexa Fluor^®^ 594-conjugated secondary antibody (1:200 dilution) was added and incubated overnight at 4 °C. Finally, the nuclei were counterstained with DAPI after washing with DPBS and examined under the fluorescence microscope.

#### 2.7.6. Evaluation of Toxicity of the Compounds

The 10 mM stock solutions of the compounds were prepared in dimethyl sulfoxide. 3D-bioprinted hepatic microtissue constructs were cultured for 7 days and then treated with 0, 2, 10, or 50 µM of the 15 selected compounds, prepared by diluting stock solutions in opti-MEM supplemented with 5% (*v*/*v*) FBS and 1% (*v*/*v*) Anti-Anti. After 24 h of the treatment, the toxicity of the compounds was evaluated by cell viability assay using the Live/Dead^®^ Viability/Cytotoxicity Kit and the 3-(4,5-dimethylthiazol-2-yl)-5-(3-carboxymethoxyphenyl)-2-(4-sulfophenyl)-2H-tetrazolium (MTS) assay using the CellTiter 96^®^ AQueous One Solution Cell Proliferation Assay kit, according to respective manufacturers’ instructions.

Briefly, for cell viability assay, the 3D human hepatic microtissue constructs were washed three times with DPBS and incubated with 200 µL/well of the combined Live/Dead assay reagents (2 μM of calcein AM and 4 μM of ethidium homodimer I (EthD-1)) for 20 min at 37 °C and 5% CO_2_. The cells were then washed with DPBS and observed under the fluorescence microscope. 

For the MTS assay, the media were removed, and the microtissue constructs were incubated with the MTS assay solution for 4 h in the dark. The absorbance values were measured at 490 nm with a microplate reader (Tecan, Austria), and the results were expressed as percentages of the control. The experiments were performed in triplicate and the results were presented as the means ± standard errors of means (SEMs).

#### 2.7.7. Live Virus Screening

Vero E6 cells were seeded into a 24-well plate at 1 × 10^5^ cells per well the day before infection. Compounds were diluted into SFM to 50 μM. SARS-CoV-2 USA/Human/USA/USA-WA1/2020 was kindly provided by the World Reference Center for Emerging Viruses and Arboviruses (WRCEVA) at the University of Texas Medical Branch (UTMB, Galveston, TX, USA). Strain of virus used was SARS-CoV-2/human/USA/USA-WA1/2020, at an MOI of 0.01, which was added to the SFM/compound and incubated for 30 min. Next, the complete growth medium was removed from the Vero E6 cells and 100 μL of the compound treated virus was added to the cells and incubated for 1 h. The cells were then washed with 0.5 mL of SFM three times. After the final wash, 0.5 mL of 50 μM compound in 2 (*v*/*v*) % FBS DMEM was added to the cells. The plate was incubated for 48 h. The supernatant was collected, spun down at 3000 rpm for 10 min and stored at −80 °C. One well per compound per experiment was performed. If a compound showed a reduction in viral replication, the experiment was performed in triplicate. A negative control with dimethyl sulfoxide (DMSO, Sigma-Aldrich) with virus and a positive control with 50 μM Remdesivir (MedChem Express) with virus were used in each experiment. Viral replication of the compound treated virus was quantified via 50% tissue culture infectious dose (TCID_50_) in Vero E6 cells seeded into 96-well plates. 10-fold serial dilutions of the supernatant were performed and plated in quadruplicate. The assay plates were read at 96–120 h post-infection. Wells were scored as either negative (no cytopathic effect) or positive (with cytopathic effect).

## 3. Results

### 3.1. Modeling and Simulations for Improved Docking Outcome

To target the SARS-CoV-2 replication on multiple fronts (e.g., ACE2–S protein, TMPRSS2, M^pro^, and Cathepsin L and K), as well as improve our screening accuracy using our selected repurposing libraries and new chemical entity libraries (ZINC database), we implemented a novel method that integrates protein flexibility/shape, adaptive biasing algorithms, machine learning from drug data (SVM and MdMD conformational sampling of the 3D target structures compound with 3D-QSAR pharmacophores using K-means analyses for a drug feedback loop (activity to inform predicted IC50 (pIC50)), and final Z-score matrix weighting to our drug modeling. We matched all FDA compounds with our realistic (X-ray derived) protein structures over a dynamic range of protein conformations with accelerated dynamics using algorithms, such as Maxwell’s demon molecular dynamics (MdMD); this approach combines docking with simulations for exploration of both ligand and protein flexibility [57,61,62,63,65,88,121]. We then refined the drug-target interface of our specific leader-like hit compounds using the quantum mechanics (QM)-based scoring within our MdMD matrix [57] to make our go/no-go assessment, which is particularly useful with NCEs and de novo compound design (DCDs). The protocol for library, structural modeling, dynamics, refinement, and hit identification as part of a pipeline is given (Figure 1B). Our Z-score approach has been described previously [116].

To improve our docking outcome, we constructed x-ray structure-based models of ACE2 bound to S protein, M^pro^, and TMPRSS2 in our MDS and virtual screening (Figure 1B and Figure S1). As S protein interfaces with ACE2 at a distinct region from the active site (Figure S1A–D), inhibition of the binding site by ligands may disrupt the ACE2–S protein interaction. Canonical inhibitors of ACE2 bind at the active site where angiotensin interacts, whereas drugs directed at the structural region for S protein binding are not overlapping with the binding site. The modulation of ACE2–S protein interaction by canonical ACE2 inhibitors is likely allosteric and suboptimal. Therefore, directly targeting the interface of the interaction should increase efficacy of the approach and block COVID viral binding, precluding entry (Figure S1). Additional investigation into the glycosylation sites of the S protein demonstrated that the ACE2 binding site is mostly unaffected by these additions (Figure S2).

#### 3.1.1. ACE2–S Protein Interaction Requires Dynamics to Reveal Binding Site

**Figure 2:**
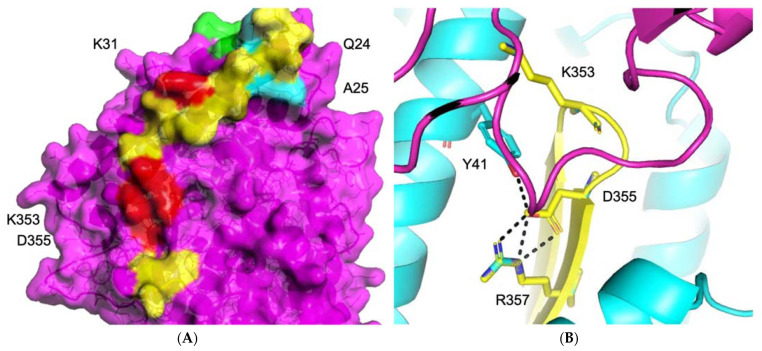
PPI region on the surface of ACE2 identifies key residues. (**A**) PPI region (yellow) on the surface of ACE2 is shown with important residues K353, D355, Y41, K31 highlighted in yellow. (**B**) Zoomed in detail panel shows beta sheet secondary structure and H-bond interactions targeted for disruption by docked small molecules.

**Figure 3:**
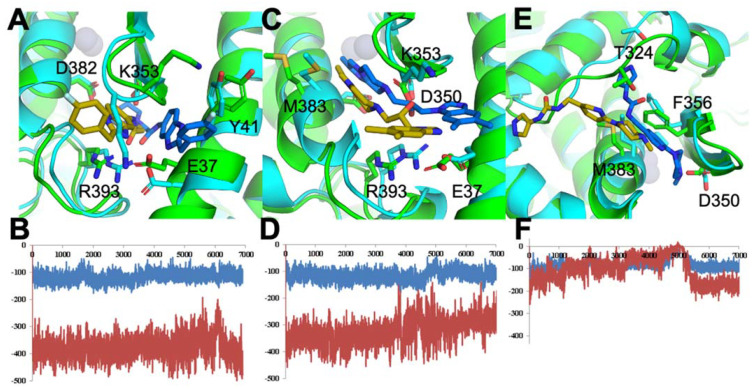
ACE2 protein docked with exemplar ligands during MD simulations and used as basis for large-scale constraint-based screening. ACE2 bound to ligand in its initial state (cyan for ACE2 and marine for ligand) vs. maximum displaced state after MDS (green for ACE2 and gold for ligand) for ligand 300 (**A**), ligand 392 (**C**), or ligand 488 (**E**). Electrostatic energy (red) and VdW energy (blue) of ligand-bound ACE2 for ligand 300 (**B**), 392 (**D**), or 488 (**F**).

To obtain the optimal interface for drug screening, we used our grid searching algorithms, as well as site mapping and protein–protein docking, to examine the protein–protein interactions surface using MDS (Figure 2, Figure 3 and Figure S1) [35,57,65,94,103,122]. The protein–protein inhibitor (PPI) interaction complex did not identify any immediate binding sites on the surface of the PPI interfaces. Nevertheless, a small pore around one single beta-sheet in the center of the PPI interaction area could be exploited as a weak point that may perturb the interface equilibrium. Using UniProt, which contains information about a number of confirmed mutations, we determined the relative potencies of PPI binding residues, identifying those that would likely affect the integrity of the complex (Figure 2). Residues K353 and Y41, which interact with D355 at the center of the PPI, are likely stabilizing its surface, potentially forming a useful “hot spot” for targeted druggability (Figure 2, Figure 3 and Figure S2).

To check whether this is true and to understand how ACE2–S protein cooperation functions, we performed two MD simulations, one with and one without the mutation of Y41A. This mutation strictly inhibits the formation of the ACE2–S protein complex. Analysis of the trajectory of the wild-type protein, which possessed an intact complex, revealed the three most stable conformations of the “hot spot” region with expanded pores inside the triangle of residues K353, D355, Y41. Since it is impossible to determine which of these three conformations is the most stable, we ran three high-throughput screenings based on the donor-acceptor atoms and hydrophobic areas of the region. We then performed three MD simulations with top pose ligands. As demonstrated in Figure 3F, some ligands failed binding within 10 ns, while other docked ligands became leaders, as determined by energetic stability, during MD and interaction energy values (electrostatic—red; Van der Waals—blue) (Figure 3B,D).

#### 3.1.2. Identification of Predicted Inhibitors to Interrupt ACE2–S Protein PPI via Docking

**Figure 4:**
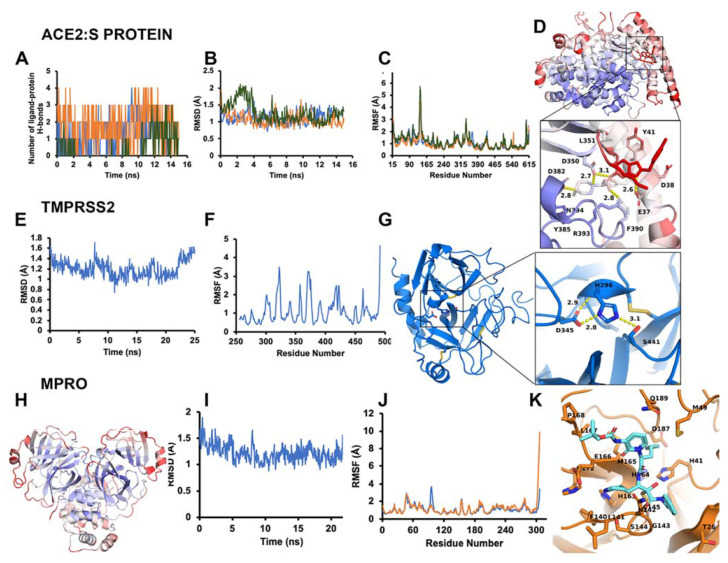
Modeling requires molecular dynamics to reflect optimal inhibitor-binding sites. (**A**–**D**) ACE2–S protein stabilization and effect of ligand binding at allosteric site. (**A**) Number of hydrogen bonds for each ligand with ACE2 against each frame of the simulation. Blue is ligand 300, orange is ligand 392, green is ligand 488. (**B**) RMSD of ACE2 across every frame in the simulation, bound to different ligands. (**C**) RMSF per residue of ACE2 in each MDS bound to different ligands. (**D**) RMSF heat-mapped onto ACE2 and ligand 300. A call-out box shows a close-up of ligand and binding site. Ligand and binding site residues represented as sticks with labels and interaction distances. The scale is a BWR gradient from 0 to 2.0 Å RMSF. (**E**–**G**) TMPRSS2 dynamics reveal the catalytically active form suitable for inhibition. (**E**) RMSD in Å across the 25 ns MDS trajectory mapped as a 2D plot. (**F**) Per residue average RMSF in Å across the trajectory mapped as a 2D plot. Disulfide bonds and catalytic triad are represented as sticks. The scale is a BWR gradient from 0 to 2.0 Å RMSF. (**G**) Post-cleavage (mature protease) extracellular domain of TMPRSS2. Call-out box shows close-up of canonical serine protease catalytic triad of mature TMPRSS2, with distances of polar contacts. (H-K) Model refinement for M^pro^ reveals ligand-binding sites suitable for docking. (**H**) Average RMSF per residue heat-mapped onto the M^pro^ structure. The scale is a BWR gradient from 0 to 2.0 Å RMSF. (**I**) RMSD of M^pro^ for each frame of the simulation. (**J**) Average RMSF per residue of M^pro^ (each chain measured separately). (**K**) M^pro^ (orange) with small-molecule inhibitor (cyan).

To identify inhibitors of the ACE2–S protein interaction via docking, we used the best-scoring compounds obtained after combination of molecular docking and molecular dynamics simulations, which feeds into the pipeline for constraint-based screening. The high-throughput screening (HTS) of a PPI library did not produce any results, since the PPI binding sites were weakly identified shallow regions (Figure 2, Figure 4A–D and Figure S1). Compounds that made good insertion into the sites situated between ACE2 and S protein were able to perturb the association of S protein with ACE2 via steric hindrance of S protein association (Figure 3). From the MDS, we detected compounds that decreased energy of stability between the ACE2–S protein complex, which is desired in an inhibitor of protein–protein interaction. As a whole, this approach identified a deep and narrow binding site to disturb the S protein interaction with ACE2 (Figure 3 and Figure 4A–D).

#### 3.1.3. Optimal Inhibitor Binding for TMPRSS2 and M^pro^ Revealed via Dynamics

To optimize the binding site of our inhibitors, we constructed a full-length (zymogen) model of TMPRSS2 (epitheliasinogen), as well as a mature version of the protease (epitheliasin), as described in our method section (Figure 4E–G). The mature protease model was used for MDS studies to generate a reference dynamical profile that can be used to assist in silico screening of TMPRSS2 inhibitors. A control experiment was also completed with the uncleaved (non-catalytic) form of TMPRSS2 to demonstrate the pocket’s instability and poor ligand-binding capacity (Figure S3) [123,124,125]. A full-length model of monomeric M^pro^ was also constructed, as well as a homodimer (Figure 4H–K and Figure S1). The structure derived from PDB code 6Y2F with its ligand was used for a consensus virtual screen [126]. In addition, we used the dimer to generate a reference dynamical profile to assist with in silico screening and study its interdomain behavior.

#### 3.1.4. TMPRSS2 Inhibitors Identified

We acquired the dimer protein sequence from the UniProt database. BLAST search showed the highest identification values against factor XI, prothrombin, kallikrein proteases (~41–42%). However, we focused on ligands that could be active against the active form of TMPRSS2 protein. Thus, we found the ligand: (2s)-1-[(2r)-2-(Benzylsulfonylamino)-5-Guanidino-Pentanoyl]-*N*-[(4-Carbamimidoylphenyl)methyl]pyrrolidine-2-Carboxamide, contained within the ChemblDB repository (CHEMBL1229259) and active against TMPRSS2, prothrombin, and Factor XI. Likewise, another docked model was recovered with macrocyclic ligand (CHEMBL3699198), called:

ethyl14-[[(E)-3-[5-chloro-2-(tetrazol-1-yl)phenyl]prop-2-enoyl]amino]-5-(methoxycarbonylamino)-17-oxo-8,16 diazatricyclo[13.3.1.02,7]nonadeca-1(18),2(7),3,5,15(19)-pentaene-9-carboxylate.

**Figure 5:**
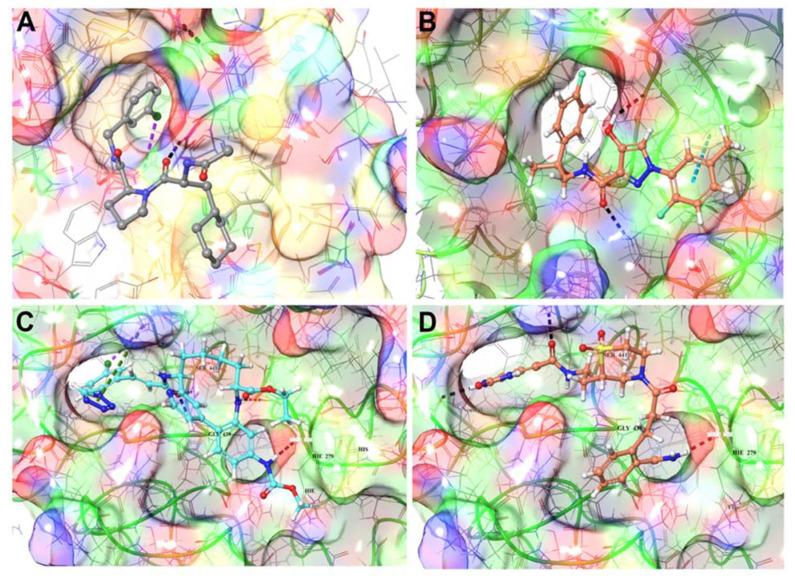
Modelled catalytically active form of TMPRSS2 bound to inhibitors. (**A**) Homology model of TMPRSS2 based on crystal structure of thrombin (3F68) is shown docked with 1-(2-fluoro-5-methylphenyl)-*N*-[2-(4-fluorophenyl)-2-hydroxypropyl]-4-hydroxy-1H-pyrazole-3-carboxamide (**B**). A proposed macrocycle-bound structure (**C**) and docked *N*-(2-4-[3-(2-Carbamoylphenyl)propanoyl]-1,1-dioxido-2-thiomorpholinyl}ethyl)-2-oxo-2,3-dihydro-*1H*-benzimidazole-4-carboxamide (**D**) as further exemplars for inhibition of Tmprss2.

We launched several molecular dynamics simulations (up to 75 ns of duration) to understand the interaction with the target protein-binding site. Figure S3 shows the initial and stable/final states of our various models (Figure 4E–G). The MD analysis provided useful results for selecting the appropriate model. After 15 ns MD, the putative binding site collapsed (Figure S3 and Figure 4E–G). Although the active form of thrombin was used for TMPRSS2 modeling, as a negative control we also examined the region with prothrombin-based binding site for completeness of the docking study (Figure S3). The overlay of the average homology model structure from MD and structure 3F68 (PDB code) was used as a template to compare protein-ligand interaction map and assign docking constraints [76]. Two optimal inhibitors for TMPRSS2 were selected for demonstration purpose in Figure 5. We also modeled Cathepsins L and K for preliminary work, since these are implicated in late-endosomal entry of the virus (Figure S4).

#### 3.1.5. M^pro^ Inhibitors Identified

**Figure 6:**
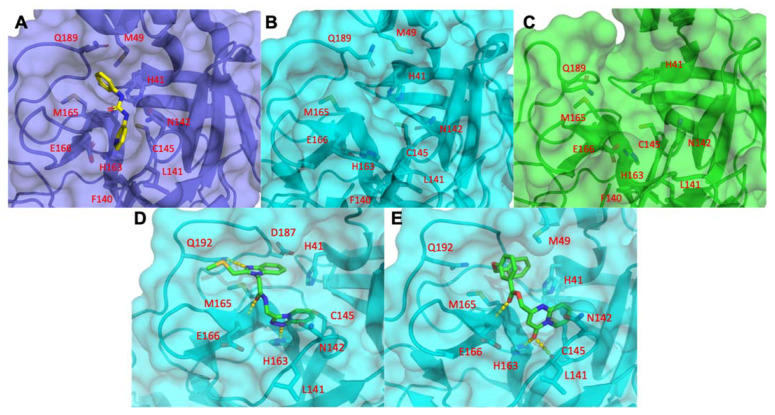
Druggability of M^pro^ is demonstrated with detailed analysis of α-Keto amide group binding using MD simulations. (**A**) The M^pro^ crystal structure PDB ID: 5R83 bound to compound containing an α-Keto amide group flanked by hydrophobic groups is shown. Sufficient structural stability of the binding site is demonstrated via comparative visualization of initial (**B**) and final (**C**) states of MD. Binding site retains its geometry and shape across the MD. Two bound states of hit compounds from the large library of compounds give further exemplars: compound **120** (**D**) and compound **247** (**E**) (Z2169338256 and Z991010090) in complex with M^pro^ protein.

For the viral main proteinase, M^pro^, a key enzyme for coronavirus replication (SARS-CoV-2), and a potential target for anti-SARS drug development, several peptidomimetics synthetized in early 2012 against SARS-CoV-1 proteases were identified as selective. There is a high degree of sequence identity between the SARS-CoV-1 and SARS-CoV-2 M^pro^. This means that SARS-focused ligands could form similar interaction maps with M^pro^ protein and offers good launching points for 3D-QSAR/machine learning-driven drug design for future iterations. To perform the virtual screening, protein structure was taken from the PDB code 7BQY complex and significant attention was paid to the interaction between the crystallized ligand from the complex and protein-binding site [127] (Figure 6). As the binding site is quite large (Figure 6A) we used a set of additional crystal structures (PDB code 6Y2F/5R83 and fragment-like compounds from https://www.diamond.ac.uk/ (accessed on 22 May 2021)) to narrow the source of possible conformations. The binding of the compounds inserted into this region demonstrated a very canonic and recurring interacting motif, represented with α-Keto amide group flanked with aliphatic or saturated rings. We then performed molecular dynamics of 75 ns for the ligand-free dimer structure of the M^pro^ to evaluate and “catch” the most flexible elements of the binding site. Our simulation revealed that the extended binding pocket was not very stable, unlike its individual subpocket, which contains the catalytic dyad cysteine (C145) and histidine (H41), among other residues (Figure 6B,C). We began our molecular docking after assigning several combinations of constraints that should define specific interactions with the protein-binding site. We performed several high-throughput screening procedures using the same set of features in different combinations of constraints by partial matching algorithm (Figure 6D–E). We then ranged docking scores and compared obtained conformations inside the binding site with the co-crystalized ligands from 7BQY, 6Y2F structures to select the most potent compounds.

### 3.2. Analysis of Identified Compounds

By disrupting the SARS-CoV-2 viral process in three different critical routes: binding, entry, and replication with our virtual screening approaches against dynamic structures, we were able to identify 350 compounds (Dataset S1) and compile data reflecting physiochemical and chemoinformatic properties. An exemplar top hit from each target is summarized for docking score in Table 1. To classify the compounds and their chemical space, we completed various regression, K-means analyses and fingerprint measurements, and provide further details about their structures and properties, including commonly evaluated traits: MW, HBA, HBD, docking score, Rule of Three (Jorgensen), Rule of Five (Lipinksi), logP_o/w_, and logS (Dataset S2). We focused on new compound searches. The MW for these initial screening compounds ranges from large fragment (~250 Da) to mature drug sized molecules (~500 Da) with only 10 of the 310 top scoring compounds being over 500 Da in size and the smallest fragment-based compound measured 178 Da. Overall the docking scores were very good with a median of approximately −7 kcal/mol using the Glide XP calculations. We also generated a list of most commonly related drugs and discuss some of our best hits to known and clinical trial drugs (Dataset S3). The general process for pruning the >30 million total chemical fragments and compounds from commercially available compounds for the initial round of virtual screening is described (Figure 1B), which reduces the primary large set to 3 million per conformation of target.

As an example, when examining some prototype compounds from our selected dataset of >300 NCEs screened from >10 million total compounds, we find the predicted interactions between drug and protein (Table S2) have some common binding modalities. When looking at the dynamical data for the drugs binding to the protein–protein site on ACE2, we find the RMSD, RMSF, and H-bond occupancy evidence strong binding capability, as calculated from three separate simulations of ACE2 with different ligands, referred to as 300, 392, and 488 (Figure 4 and Figure S1). These observations can be applied to generate constraints for additional virtual screening to improve the performance at higher throughput. Based on these results, ligand 392 reduced the overall RMSD and per residue RMSF, while maintaining strong hydrogen bonds, as demonstrated by its greater occupancy during the simulation (Table S1). This information, particularly H-bond occupancy and modulation of interface residue RMSFs, can be used in conjunction with docking and other data to profile the compounds more thoroughly (Figure 4). In some cases, where constraints were utilized, the docking score underrepresents the compound and testing is needed to obtain important single-point data to clarify actives from non-actives, as well as determine the real IC50s for the selected active compounds. We will enrich our dataset with the top compounds for future rounds of parallel chemical screening and eventual de novo chemical design for novel chemical entities. Current results of our approach are presented on all three targets (ACE2, TMPRSS2, M^Pro^).

### 3.3. Screening FDA-Approved Drugs for Repurposing

For each of our targets, we screened for hits from a library of FDA-approved compounds alongside the more extensive library of NCEs. Our final result across all three targets identified a total of 350 specific compounds, with 167 against ACE2, 40 against TMPRSS2, and 103 against M^pro^. Among these are FDA-approved drugs that could be repurposed: 21 against ACE2, 11 against TMPRSS2, and 8 against M^pro^ (Supplemental Dataset S1).

#### 3.3.1. ACE2 Repurposing Drugs (FDA Set)

Isoprenaline hydrochloride (isoprotenerol) is an adrenoreceptor agonist that can be repurposed as a vasopressor to augment cardiovascular function with a beta-receptor side benefit of bronchodilation to improve breathing function. Metaraminol bitartrate, a stereoisomer of meta-hydroxynorephedrine, is a potent sympathomimetic amine to raise blood pressure. Atenolol and nadolol are beta-receptor blocking agents used in chronic hypertension, a comorbid risk factor in COVID-19 patients. Propafenone is an anti-arrhythmic agent approved for patients with life-threatening ventricular tachycardia. Levosulpiride is an atypical antipsychotic medication with prokinetic function that can be used in patients with agitated delirium and gut immotility. Valganciclovir hydrochloride is an antiviral agent used for cytomegalovirus (CMV), varicella zoster virus (VZV), and preventative medication in HIV patients [128]. Recent data show that COVID-19 causes depletion of CD8^+^ T cells [129]. Amikacin sulfate and cephalexin are antibiotic antibacterial drugs that can treat bacterial super-infection. Prochlorperazine dimaleate is a phenothiazine derivative prescribed in medicine for nausea. Isoetharine mesylate is a selective adrenergic beta-2 agonist and fast-acting aerosolized bronchodilator for COVID-19 respiratory distress. Benserazide hydrochloride is an aromatic l-amino acid decarboxylase (DOPA decarboxylase inhibitor) used with levodopa for the treatment of Parkinson’s disease. Glucosamine hydrochloride is a constituent of cartilage and used t treat osteoarthritis joint pain. S4701 or 2-deoxy-d-glucose (2D-DG) compound can induce ketogenic state, a powerful pathway involved in reducing systemic inflammation. Inulin is a natural prebiotic agent that enhances GI function and digestion by increasing prebiotic GI homeostasis critical to stabilize downstream anti-inflammatory effects and prevent overgrowth of harmful bacteria. Metaproterenol is a bronchodilator (beta-2 receptor agonist) that is commonly used to treat a variety of respiratory disorders including asthma, COPD, bronchitis and wheezing associated with viral pneumonias in clinical practice. The novelty of this drug is that is aerosolized and can be given as a breathing treatment to reach the lungs, which have a tremendous surface area, and enter the blood rapidly. By inhalation this drug acts rapidly and potentially alone or in combination with other aerosolized drugs or oral or IV combination drugs. Its inhalational route of delivery also can reach alveolar type II cells which express ACE2 for dual synergism. Metaraminol bitartrate, a stereoisomer of meta-hydroxynorephedrine, is a potent sympathomimetic amine. This drug is used in patients with hypotension or low blood pressure. COVID-19 hospitalized patients in the intensive care unit (ICU) setting often need vasopressor agents to raise blood pressure in a condition called shock (dangerously low blood pressure) from COVID-19 disease or sepsis. Therefore, metaraminol has a dual purpose of antiviral function at ACE2 docking site/entry as well as helping with systemic blood pressure in those acutely ill COVID-19 patients. This drug has immediate repurposing use in this patient population.

#### 3.3.2. M^pro^ Repurposing Drugs (FDA Set)

Atorvastatin is a statin drug with anti-inflammatory, immunomodulatory [130] and endothelial benefits [131,132]. Carbenicillin disodium is a penicillin-derivative antibacterial antimicrobial agent. Catechins are derived from plants with many beneficial properties in human health including anticancer, anti-obesity, antidiabetic, anti-cardiovascular, anti-infectious, hepatoprotective, and neuroprotective effects [133]. These substances fall outside FDA purview since supplements generally have a wide safety margin that will be tested on the multi-drug platform. Epicatechine S5105 is a naturally occurring flavonoid found in chocolate with anti-sarcopenic effects on skeletal muscle [134]. Ivosidenib is an experimental drug for treatment of several forms of cancer. Bezafibrate is a fibrate lipid-lowering drug, which creates a favorable anti-inflammatory ratio against cardiovascular diseases. PF299804 or dacomitinib is an EGFR inhibitor used in cancer therapeutics. Metaproterenol is a bronchodilator (beta-2 receptor agonist) that is commonly used to treat a variety of respiratory disorders with viral pneumonias in clinical practice. Carbenicillin disodium is a penicillin-derivative antibacterial antimicrobial agent that as mentioned above can be used in conjunction with other anti-SARS-CoV-2 agents to shut down antiviral effects and used in combination with those COVID-19 patients with secondary super-infection with bacterial infection of lung, blood, or skin.

#### 3.3.3. TMPRSS2 Repurposing Drugs (FDA Set)

Bumetanide is a loop-diuretic used to treat edema. Aloin is an anthraquinone glycoside found naturally in aloe vera plants, a natural cathartic, and decreases 16S rRNA sequencing of dysbiosis-producing butyrate producing bacterial species via an emodin breakdown product [135]. Emodin blocks ACE2 and viral docking [136]. Salbutamol sulfate (albuterol) is a bronchodilator used in various breathing disorders. S4953 usnic acid is a naturally occurring dibenzofuran derivative found in lichen plant species and in some kombucha teas, with adrenergic function to raise blood pressure and to act as a potential bronchodilator. Usnic acid is an active ingredient and a preservative in others and has a wide array of antimicrobial action against human and plant pathogens with antiviral, antiprotozoal, antiproliferative, anti-inflammatory, and analgesic activity [137]. Avanafil belongs to a class of medications called phosphodiesterase (PDE) inhibitors, which are pulmonary artery and circulation dilators. S3612 Rosmarinic acid is a naturally occurring compound found in rosemary and sage plants that has a broad range of antimicrobial activity including antiviral activity including HIV [138]. Ractopamine is a beta-agonist used for bronchodilatation. Neohesperidin dihydrochalcone (NHDC) is a naturally derived plant sweetener (bitter orange) with anti-TMPRSS2 effects. Cidofovir and zidovudine (ZDV) are both antiviral drugs used in HIV patients.

### 3.4. Results of In Vitro Assays for New Chemical Entities (Novel Compounds)

The liver is the largest metabolic organ, and plays an important role in drug detoxification and metabolism [139]. Hepatocytes comprise ~80% of the liver mass and play a central role in liver functions [139,140]. In vitro hepatocyte culture methods that mimic the in vivo-like microenvironment provide a potential platform for evaluating drug toxicity and screening of drugs which, in turn, improve the success rate of drug discovery in clinical trials. In recent years, the 3D bioprinting method have been employed to develop high-content, physiologically relevant in vitro 3D liver models that reproduce the structures and functions of their native counterpart, for drug screening with accuracy [141,142,143,144,145,146,147,148].

In this study, the 3D hepatic microtissue constructs were fabricated by the DLP bioprinting approach using a custom-built bioprinter [120] and HepG2-C3A cells embedded in 5% (*w*/*v*) GelMA with the Ru/SPS visible light photoinitiator as the bioink. This bioprinting system utilized a visible light source from a projector to bioprint the hepatic microtissue constructs based on the digital pattern input in the system (Figure S5A). GelMA has been widely used to fabricate in vitro tissue models due to its controllable physicochemical properties, biodegradability, and on-demand photocrosslinkability [149]. The 3D human hepatic microtissue construct, approximately 5 mm in overall size and 200 µm in thickness, comprised of an array of hexagonal lobules (each approximately 200 µm in size) arranged in a honeycomb-like structure, resembling the native liver lobules (Figure S5B,C). These microtissue constructs were cultured for 7 days to achieve a desired high cell density.

The viability of the HepG2-C3A cells within the hepatic microtissue constructs was assessed at day 7 using Live/Dead staining, comprised of calcein AM and ethidium homodimer I (EthD-1), respectively. Calcein AM is a non-fluorescent cell-permeable compound that is hydrolyzed into the green fluorescent anion calcein by intracellular esterase in living cells, whereas EthD-1 is a dead cell-specific red fluorescent dye that binds to DNA. The viability of cells within the hepatic microtissue constructs were found to be more than 96% at day 7.

The morphology of the HepG2-C3A cells within the hepatic microtissue constructs was assessed by F-actin staining of the cells at day 7. F-actin is the most abundant cytoskeletal filamentous protein that determines the shape, stiffness, and movement of the cell surface, and also facilitate the transduction of mechanical signals as well as generate the intracellular forces required for many cellular functions [150] Thus, the proliferation and morphology of HepG2-C3A cells within the hepatic microtissue constructs were assessed by F-actin staining using Alexa Fluor^®^ 594-phalloidin. The cells were counterstained with DAPI for nuclei and imaged using fluorescence microscopy. Fluorescence micrographs showed that these cells were homogenously distributed within the hepatic lobules, suggesting good functions of the cells as well as the resulting microtissues (Figure S5D).

The functionality of the HepG2-C3A cells within the hepatic microtissue constructs was further assessed by immunostaining of the cells for CYP3A4 (phase I metabolic enzymes) [151,152,153]. CYP3A4 represents one of the CYP450 enzymes responsible for metabolism of drugs [154,155]. CYP3A4 is predominantly expressed in the liver and is involved in most aspects of phase I drug metabolism, including oxidation, reduction, hydrolysis, and dehydrogenation [153,156]. At day 7, the HepG2-C3A cells within the hepatic microtissue constructs were fixed and immunocytochemical staining was performed with CYP3A4 antibody. The cells were counterstained with DAPI for nuclei and imaged using fluorescence microscopy. Fluorescent micrographs of the immunostained HepG2-C3A cells exhibited strong expression of CYP3A4 (Figure S5E). These bioprinted hepatic microtissue models are well-established by us and others [141,157], and these results obtained here validated the fact that these 3D-bioprinted volumetric hepatic microtissues represent appropriate in vitro models for drug testing as well as functional and mechanistic studies.

**Figure 7:**
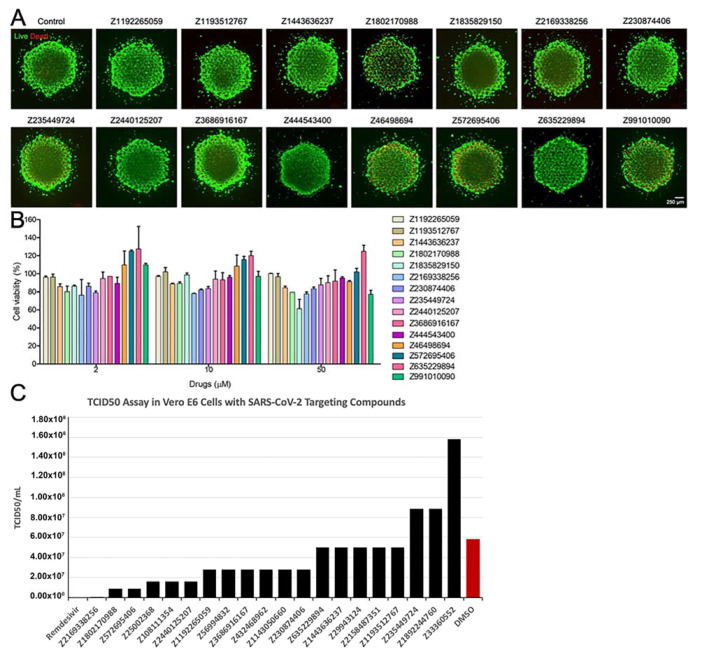
In vitro compound evaluation. (**A**) Fluorescence images showing live (green)/dead (red) staining after 24 h of treatment by the compounds (50 µM). (**B**) Quantification of cell metabolic activities by the MTS assay after 24 h of treatments by the compounds at different concentrations. (**C**) Quantification of TCID_50_ assay in Vero E6 cells when treated with Remdesivir, DMSO, or a SARS-CoV-2-targeting compound.

The 3D bioprinted hepatic microtissues were subsequently used to test the toxicity of the 15 selected compounds. The hepatic microtissue constructs were treated with 0, 2, 10, or 50 µM of these compounds for 24 h and the toxicity of the compounds was evaluated by both the cell viability assay and the MTS assay. Both Live/Dead and MTS assays revealed that none of these compounds showed significant differences in the viability and metabolic activities of the hepatic microtissues compared to those of the control without treatment (Figure 7). Thus, these compounds did not exhibit any significant toxicity in our preliminary pre-clinical screening using these physiologically relevant, bioprinted 3D hepatic microtissues, at all doses evaluated. In addition, live virus screening results in Vero E6 cell line using a TCID_50_ assay identified some new compounds with efficacy similar to the positive control Remdesivir (Figure 7), though working through different mechanism of viral inhibition. These compounds different unique targets and subsequent efficacy/potency inhibiting COVID-19 will allow us to utilize these unique modes of action in combination screening for synergistic and/or improved antiviral effects.

## 4. Discussion

### 4.1. Clinical Unmet Need for COVID-19 Acute Therapeutics

There is a critical unmet need for therapeutics to treat the acute phase of COVID-19. Efforts to vaccinate the global population are underway, but 160 million people have been confirmed infected globally (>3.3 million deaths), with over 20% of cases occurring within the United States as of mid-May 2021. In order to accelerate drug discovery, a design funnel using high-powered artificial intelligence was used to screen millions of compounds against macromolecular mechanistic viral targets. At the back end of this funnel, 40 drug candidates emerged, many of which may represent repurposing candidates for use in humans due to known safety and tolerability profiles. However, the approach with the highest probability of overall clinical therapeutic success may be not a single drug therapy for this viral RNA disease but rather a multi-pronged drug approach gleaned from decades of HIV research. A multi-drug approach for HIV has improved survival, markedly reduced viral loads, and vastly improved management of the disease by preventing AIDS end-stage fatal complications. We therefore suggest that a multi-faceted drug approach for SARS-Cov-2 may prove superior by attacking three viral entry and replication cycle sites simultaneously: ACE2 receptor docking site and entry, TMPRSS2 endosomal packaging, and M^Pro^ viral replication. Multiple drug targets for each of the three sites also allow permutations and optimization for combinatorial success.

### 4.2. Comparison of FDA-Approved Compounds Identified from Another Recent Screen

A recent study that screened commercially available >10,000 clinical-staged and FDA-approved small molecules against SARS-CoV-2 in a cell-based assay (Riva et al., 2020) identified interesting compounds for alternative targets that complement our results. These FDA-approved compounds included MDL-28170, a selective Cathepsin B inhibitor; VBY-825, a non-specific Cathepsin B, L, S, V inhibitor; Apilimod, an inhibitor of production of the interleukins IL-12 and IL-23; Z-LVG-CHN2, a tri-peptide derivative inhibitor for cysteine proteinases; ONO 5334, a selective Cathepsin K inhibitor; and SL-11128, a polyamine analog designed against *E. cuniculi*, a antimicrobial agents used as an adjuvant treatment for opportunistic AIDS-associated infections. Overall, these compounds are Cathepsin-centric or antibiotic in nature, with little to no effect on our intended targets (TMPRSS2, ACE2, M^Pro^). Additional top hits identified by Riva et al. include: AMG-2674, an AMGEN compound inhibitor of TRPV-1 (Vanilloid Receptor); SB-616234-A that possesses high affinity for human 5-HT1B receptors; SDZ 62-434 that strongly inhibited various inflammatory responses induced by lipopolysaccharide (LPS) or function-activating antibody to CD29; Hafangchin A (also called “Tetrandrine”), a bis-benzylisoquinoline alkaloid, which acts as a calcium channel blocker; Elopiprazole an antipsychotic drug of the phenylpiperazine class (antagonist at dopamine D2 and D3 receptors and an agonist at serotonin1A receptors) that was never marketed; YH-1238, which inhibits dipeptidyl peptidase IV (DPP-IV) enzyme prolonging the action of the incretin hormones, glucagon-like peptide-1 (GLP-1) and glucose-dependent insulinotropic polypeptide (GIP); KW-8232, an anti-osteoporotic agent that can reduce the biosynthesis of PGE2; Astemizole, an antihistamine; *N*-tert-butyl Isoquine (also called “GSK369796”), an antimalarial drug candidate; and Remdesivir, a broad-spectrum antiviral medication developed by the biopharmaceutical company Gilead Sciences. Again, none of these compounds were geared toward targeting TMPRSS2 or M^pro^, and are also not specific to ACE2. While the lack of overlap may be surprising, results generated by Riva and colleagues are not in opposition to our findings and both approaches can complement each other. Most importantly, these FDA-approved compounds can be combined with our set 310 compounds that have been demonstrated to have low toxicity issues based on our chemoinformatics filtering (Figure 1B). All NCE compounds identified were chemical moieties that do not overlap with any FDA-approved drugs. Altogether, the data presented here complement previously generated data and should help prioritize and rapidly identify safe treatments for COVID-19. Future work will rely on advanced 3D-QSAR, fragment-based drug design principles for de novo drug optimization.

### 4.3. Selective AI-SARS-Cov-2-Targeting and Drug Repurposing Data—ACE2, TMPRSS2, M^pro^

Among the millions of potential COVID-19 drugs screened, the majority of the final 40 drug candidates have known clinical uses and/or FDA approval for a primary indication (e.g., hypertension, cardiac indication, hyperlipidemia) with well-established patient safety and tolerability profiles from large Phase III human trials and post-market (Phase IV) analyses. These large human datasets provide both a clinically significant and scientifically innovative window of opportunity to test 40 compounds on the multi-drug platform, and, in conjunction, observe longitudinal human survival outcomes of COVID-19 patients on these drugs for comparative effectiveness within established and ongoing patient registries. An emerging example of this important parallel is ACE2 pathway drugs (ACE inhibitors and angiotensin receptor blocking drugs), which are increasingly observed in humans with COVID-19 to be associated with improved survival advantage [158,159,160,161,162]. However, there is a scientific knowledge gap within human registry data regarding a scientifically robust and testable translational platform to test mechanistic effects of these different molecular compounds. Therefore, creation of a “pandemic platform” using newer technology of AI drug high-throughput screening combined with animal multi-drug screening models creates an early Phase I/II safety, tolerability and early efficacy platform which is rapidly needed to expedite bedside human use for the COVID-19 pandemic, and as a platform that can be used in future pandemics.

### 4.4. NCE Set of Compounds

A flurry of activity to identify compounds for SARS CoV-2 targets has been underway globally. In our approach, we introduce our novel Maxwell’s demon molecular dynamics method for screening flexibility required to obtain rare and essential conformational transitions and pathways to find the most likely druggable state. We also used our quantum docking technique (QM-driven adaptive molecular dynamics scanning docking) [57] to identify compounds effective for targeting ACE2, TMPRSS2 and M^pro^. The compounds identified by our large-scale in silico platform can be experimentally validated as binders for intended targets and for efficacy in models of the disease, evaluated for EC_50_/safety-toxicity data, and carried into hit-to-lead and lead optimization in a drug development pipeline. Structural studies such as X-ray crystallography will also be important to generate structural SAR data for these efforts.

In sum, our leading edge in silico methods incorporating structural dynamics have produced a set of 350 candidate compounds suitable for screening in biological disease models. Among these, 40 FDA-approved compounds are eligible for rapid clinical trial testing. Additionally, our results bring forward 310 NCEs predicted to possess potency and specificity for viral or host accessory proteins to lower the viral load. Moreover, this resource offers the community a set of chemical tools to probe the behavior of these enzymes essential for SARS-CoV-2 progression, namely, binding, entry and replication. As SARS-CoV-2 is already endemic, the rapid identification of effective antivirals remains a paramount focus until we have generated herd immunity via global vaccination campaigns. We have expectation for >35 effective compounds based on our current 11% hit rate that meets expectations similar to that found in Remdesivir as an antiviral agent, which can then be further tested in our BSL-3 lab.

## Figures and Tables

**Table 1:** Top 40 FDA predicted compounds for ACE2–S protein, M^Pro^, and TMPRSS2.

Drug	Synonyms	Predicted Protein	In Silico Score	Target	CAS
Metaproterenol Sulfate	Orciprenaline Sulfate	ACE2	−8.05	Others	5874-97-5
Isoprenaline HCl	Isuprel, Isadrine, Euspiran, Proternol, NSC 37745, NSC 89747	ACE2	−7.44	Adrenergic Receptor	51-30-9
Epinephrine HCl	N/A	ACE2	−7.12	Adrenergic Receptor	55-31-2
Levosulpiride	N/A	ACE2	−6.87	Dopamine Receptor	23672-07-3
Metaraminol Bitartrate	Metaradrine Bitartrate	ACE2	−6.84	Others	33402-03-8
Valganciclovir HCl	N/A	ACE2	−6.58	Antifection (Anti-Infection)	175865-59-5
Isoprenaline HCl	Isuprel, Isadrine, Euspiran, Proternol, NSC 37745, NSC 89747	ACE2	−6.45	Adrenergic Receptor	51-30-9
S4817 Atenolol	Tenormin, Normiten, Blokium	ACE2	−6.35	β1 Receptor, β2 Receptor	29122-68-7
S3783 Echinacoside	N/A	ACE2	−6.09	Others	82854-37-3
Propafenone	Rythmol SR, Rytmonorm	ACE2	−6.04	Sodium Channel	34183-22-7
Amikacin sulfate	BB-K8	ACE2	−5.98	Antifection	39831-55-5
Pro-Chlorperazine Dimaleate Salt	Prochlorperazin, Compazine, Capazine, Stemetil	ACE2	−5.79	Dopamine Receptor	30718
Isoetharine Mesylate	N/A	ACE2	−5.47	Others	7279-75-6
Levosulpiride	N/A	ACE2	−6.87	Dopamine Receptor	23672-07-3
S5023 Nadolol	Corgard, Solgol, Anabet	ACE2	−5.16	Androgen Receptor	42200-33-9
Benserazide HCl	Ro-4-4602	ACE2	−5.93	Dopamine Receptor	14919-77-8
S3694 Glucosamine (HCl)	2-Amino-2-Deoxy-Glucose HCl	ACE2	−5.57	Others	66-84-2
S4701 2-Deoxy-d-Glucose	2-Deoxyglucose, NSC 15193	ACE2	−5.18	Others	154-17-6
Inulin	N/A	ACE2	−5.18	Others	9005-80-5
Cephalexin	Alcephin, Cefablan, Keflex, Cefadin, Tepaxin	ACE2	−5.11	Antifection	15686-71-2
S4722 (+)-Catechin	Cianidanol, Catechinic Acid, Catechuic Acid	M^Pro^	−6.73	Others	154-23-4
S4723 (−) Epicatechin	l-Epicatechin, (−)-Epicatechol	M^Pro^	−6.32	Others	490-46-0
S5105 Proanthocyanidins	Condensed Tannins	M^Pro^	−6.19	Others	20347-71-1
Carbenicillin Disodium	N/A	M^Pro^	−5.78	Antifection	4800-94-6
AG-120 (Ivosidenib)	N/A	M^Pro^	−5.52	Dehydrogenase	1448347-49-6
Atorvastatin Calcium	N/A	M^Pro^	−5.39	HMG-CoA Reductase	134523-03-8
Bezafibrate	N/A	M^Pro^	−4.93	PPAR	41859-67-0
PF299804	N/A	M^Pro^	−4.34	EGFR	1110813-31-4
Bumetanide	Bumex	TMPRSS2	−6.5	Others	28395-03-1
Aloin	Barbaloin	TMPRSS2	−6.45	Tyrosinase	1415-73-2
Salbutamol Sulfate	Ventolin, Asthalin, Asthavent	TMPRSS2	−6.1	Adrenergic Receptor	51022-70-9
S4953 Usnic Acid	Usniacin	TMPRSS2	−5.8	Others	125-46-2
Avanafil	N/A	TMPRSS2	−5.62	PDE	330784-47-9
S3612 Rosmarinic Acid	Rosemary Acid	TMPRSS2	−5.6	IKK-β	20283-92-5
S5105 Proanthocyanidins	Condensed Tannins	TMPRSS2	−5.51	Others	20347-71-1
Ractopamine HCl	N/A	TMPRSS2	−5.22	Others	90274-24-1
Neohesperidin Dihydrochalcone	Neohesperidin DHC	TMPRSS2	−5.2	Others	20702-77-6
Cidofovir	Vistide	TMPRSS2	−5.18	Others	113852-37-2
Zidovudine	Azidothymidine	TMPRSS2	−5.02	Reverse Transcriptase	30516-87-1

## Data Availability

The data presented in this study are available on request from the corresponding author.

## Supplementary Materials

The following are available online at https://www.mdpi.com/article/10.3390/biom11060787/s1, Table S1: Hydrogen bond occupancy over 15 ns MDS trajectory for each ligand with ACE; Figure S1: ACE2–S protein interface and indication of allosteric site relative to active binding site; Figure S2 Glycosylation sites of Ace2 protein; Figure S3. Gradually crumbling binding site; Figure S4. Proteases cathepsin L and K can be also used in blocking entry of COVID-19 during late-endosome progression; Figure S5. 3D bioprinting of the 3D human hepatic microtissue model and functional characterizations.
