# Supplementary material for: Attacking COVID-19 Progression Using Multi-Drug Therapy for Synergetic Target Engagement
Source: Biomolecules. 2021 May 23;11(6):787. doi: 10.3390/biom11060787 (PMC8224684; doi:10.3390/biom11060787)
Supplement: Supplementary file 1 [file biomolecules-11-00787-s001.zip › biomolecules-1203810 - supplementary.pdf]

Supplementary Materials

Attacking COVID-19 Progression using Multi-Drug Therapy for Synergetic Target Engagement

Mathew Coban[1], Juliet Morrison PhD[2], Sushila Maharjan[3], David Hyram Hernandez Medina[3], Wanlu Li[3], Yu Shrike Zhang[3], William D. Freeman MD[4], Evette Radisky PhD[1], Karine G. Le Roch PhD[5], Thomas R. Caulfield, PhD[* 1,6-9]

1 Department of Cancer Biology, Mayo Clinic, 4500 San Pablo Road South, Jacksonville, FL, 32224 USA
2 Department of Microbiology and Plant Pathology, University of California, Riverside, 900 University, Riverside, CA, 92521 USA
3 Division of Engineering in Medicine, Department of Medicine, Brigham and Women's Hospital, Harvard Medical School
4 Department of Neurology, Mayo Clinic, 4500 San Pablo South, Jacksonville, FL, 32224 USA
5 Department of Molecular, Cell and Systems Biology, University of California, Riverside, 900 University, Riverside, CA, 92521 USA
6 Department of Neuroscience, Mayo Clinic, 4500 San Pablo South, Jacksonville, FL, 32224 USA
7 Department of Neurosurgery, Mayo Clinic, 4500 San Pablo South, Jacksonville, FL, 32224 USA
8 Department of Health Science Research (BSI), Mayo Clinic, 4500 San Pablo South, Jacksonville, FL, 32224 USA
9 Department of Clinical Genomics (Enterprise), Mayo Clinic, Rochester, MN 55905, USA

* Correspondence: caulfield.thomas@mayo.edu; Tel.: +1 904-953-6072

Table S1. Hydrogen bond occupancy over 15 ns MDS trajectory for each ligand with ACE2.

Ligand	Ligand 300 w/ ACE H-bonds			Ligand 392 w/ ACE H-bonds			Ligand 488 w/ ACE H-bonds		
**Kind / %**	**donor**	**acceptor**	**occupancy**	**donor**	**acceptor**	**occupancy**	**donor**	**acceptor**	**occupancy**
**Type**	drug	D350-Side	31.41%	drug	D350-Side	67.77%	drug	Q325-Main	11.96%
**Type**	R393-Side	drug	30.32%	D350-Side	drug	42.52%	drug	D355-Side	11.63%
**Type**	drug	D350-Side	28.16%	drug	D350-Side	33.55%	drug	Y41-Main	9.97%
**Type**	drug	E37-Side	24.91%	drug	D382-Side	23.59%	drug	D350-Side	6.64%
**Type**	drug	D382-Side	19.13%	drug	G352-Main	4.65%	T324-Side	drug	1.00%
**Type**	drug	E37-Main	2.53%	drug	D38-Side	4.32%	drug	N322-Main	0.66%
**Type**				drug	E37-Main	0.66%	drug	K353-Main	0.66%
**Type**							drug	M383-Main	0.33%
**Type**							Q325-Main	drug	0.33%
**Type**							Q325-Side	drug	0.33%

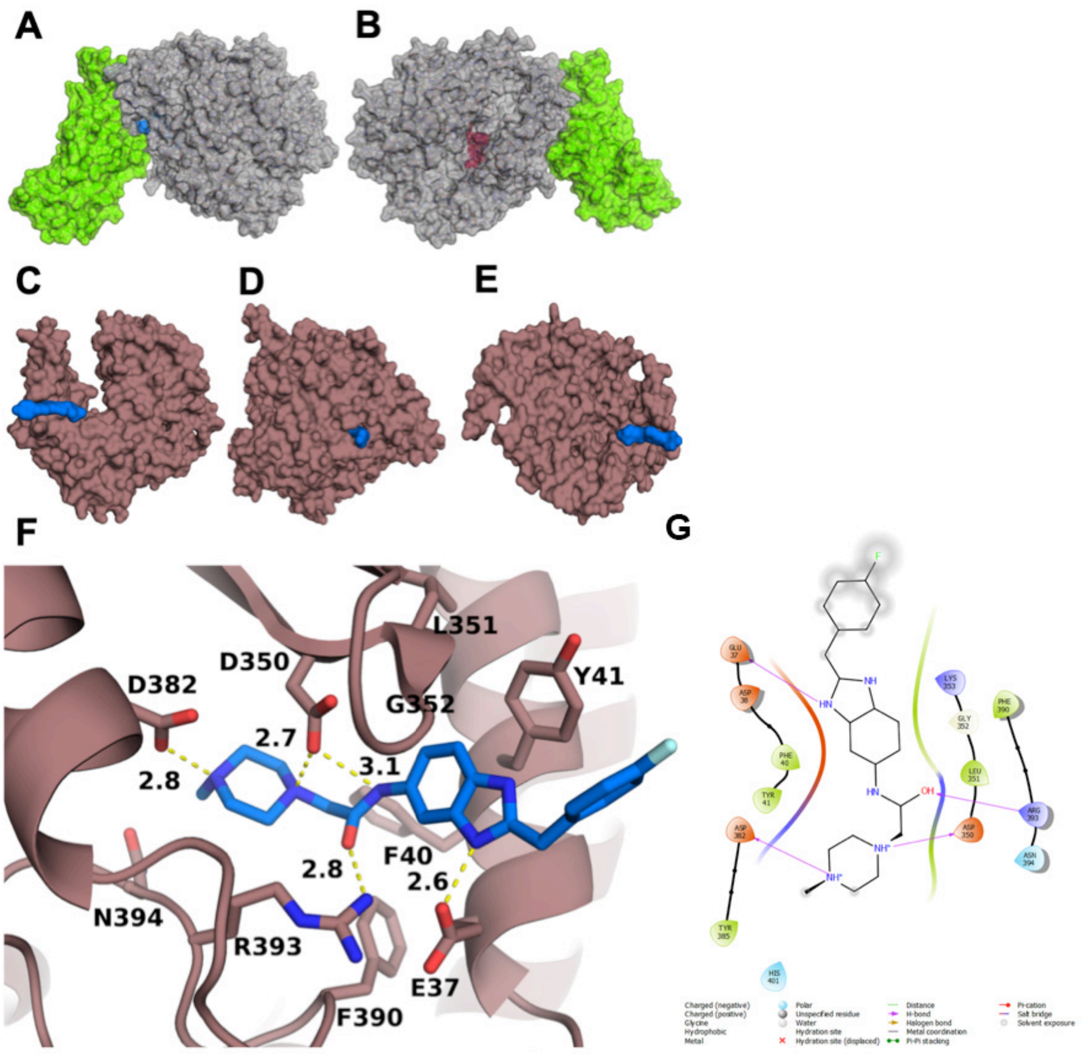

Figure S1: Ace2:S protein interface and indication of allosteric site relative to active binding site. **(A)** Sagittal view of ACE2 (gray) interface with RBD of COVID19 S-protein (green); the blue surface highlights the binding site for ligands that disrupt the interface between the two proteins. **(B)** ACE2 (gray) and S-protein (green) sagittal view. In maroon is the active site of ACE2. **(C)** ACE2 (salmon) with ligand 300 (blue) rendered as surfaces. 50% left side slab to examine deep insertion in more detail. **(D)** Full surface view of ACE2 and ligand 300. **(E)** 50% right side slab to examine deep insertion in more detail. **(F)** Example of docked compound that disrupts interface between ACE2 and S-protein. Close-up of binding site of ACE2 (salmon) and ligand 300 (blue) with residues and polar contact distances labeled. **(G)** Ligand Interaction Diagram rendered with Maestro for ACE2 with ligand 300 at the allosteric site impacting S-protein binding from SAR-CoV2. This 2D "flat" representation shows the interactions at this particular compounds interface on Ace2 that would interfere with S protein binding. In particular, extending from deeply inserted to superficial, the interactions are described in the subsequent sentences. D382 and D350 are hydrogen bond acceptors (side chains) from the opposite NH+ on the piperazine-like ring deeply inserted into the binding pocket. R393 is a hydrogen bond donor (side chain) to the alcohol group connecting the piperazine-like ring to the fused ring. E37 is a hydrogen bond acceptor (side chain) to one of the NH on the fused ring. The fluorocyclohexane group is entirely solvent-exposed at the mouth of the binding pocket.

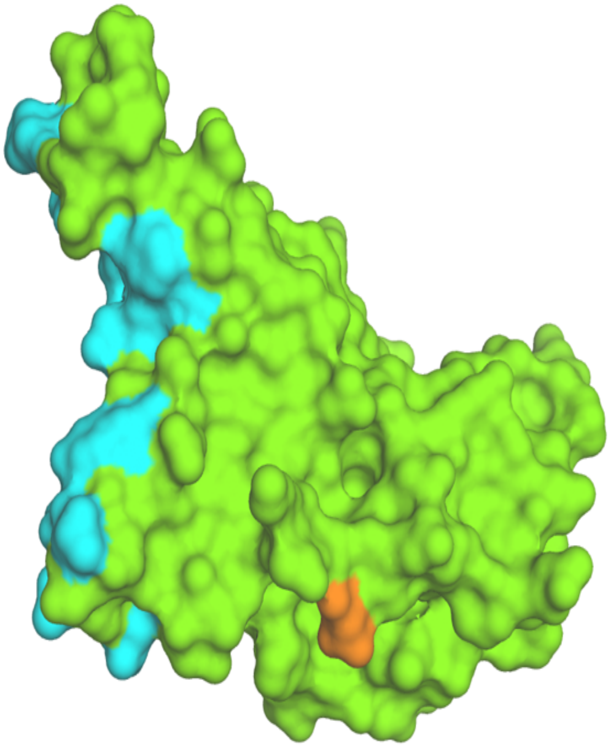

Figure S2 Glycosylation sites of Ace2 protein (D616G highlighted red). Although glycosylation sites at residues N165, N234, N343 from S-protein (PDB code 6VSB), are nearby the ACE2:S-protein binding interface, they do not overlap and interfere with the protein-protein interface, offering an adjacent site is readily available for PPI docking (S-prot glycosylation analysis: DOI: 10.1126/science.abb9983; 10.1101/2020.04.29.069054}. The majority of glycosylation sites are not on the RBD (Fig. S2), the glycosylation site that is actually present on the RBD, N343, is not in 3D proximity to the binding interface. Recently, a variant of the S-protein, D614G, was identified to possess enhanced transmissibility and resistance to contemporary interventions and this site is not present on the RBD. Neither the glycosylation sites, nor the enhanced transmissibility variant D614G, are within the 3D proximity to the drug binding site for our targeted protein-protein interface disrupting therapeutics for Ace2.

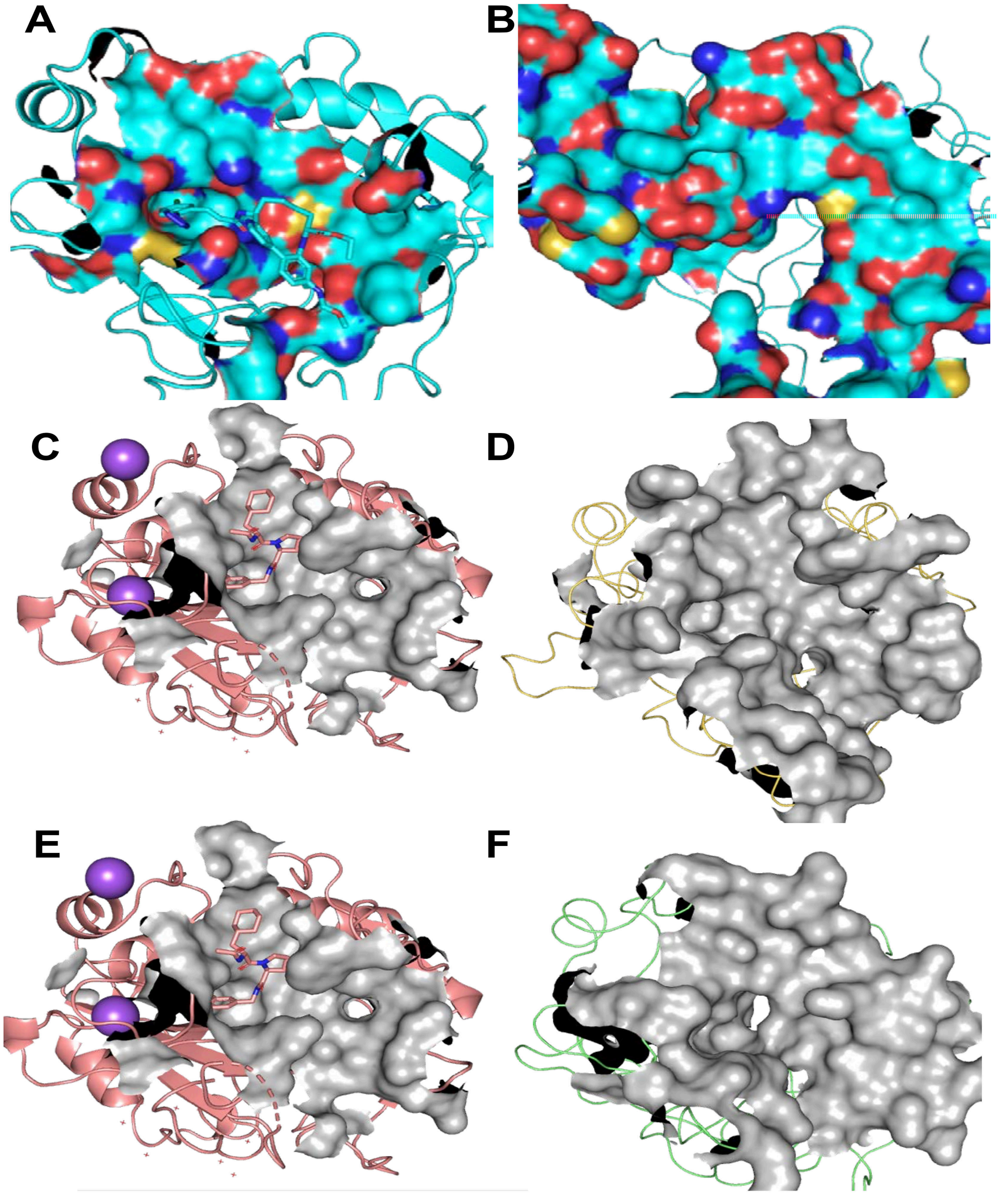

Figure S3. Gradually crumbling binding site. (A) Initital and (B) Final states - of the protein model, while catalytically active state has better preserved binding site. Prothrombin binding site (PDB 3F68) with its inhibitor (C) and the final state of Prothrombin (D) are shown. Again, Prothrombin binding site (3F68) with its inhibitor (E) and proposed structural model – a prothrombin-based homology model of TRPMSS2 (F), which looks more accurate then previous (B) model structure. This version maintains structural stability and is good candidate for drug docking with ligands. Purple spheres are constraints used to impose good relative positioning.

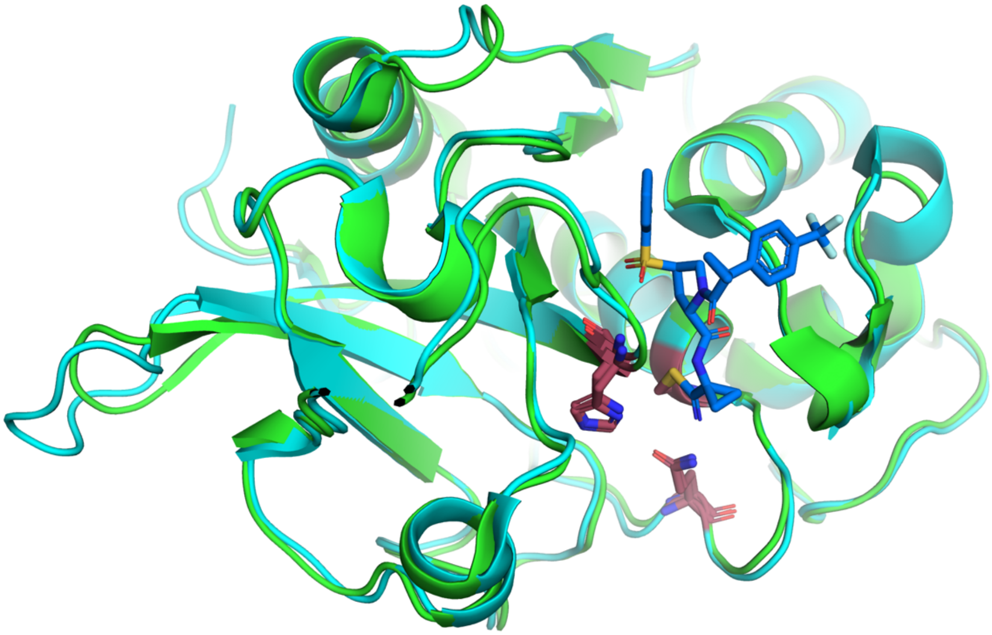

Figure S4. Proteases cathepsin L and K can be also used in blocking ENTRY of COVID-19 during late-endosome progression. Top panel. Structures for cathepsin K (PDB code: 4N8W, green) and cathepsin L (PDB code: 2YJB, cyan), shown with an inhibitor (blue). The active site residues are colored maroon. These represent additional host protease targets at another stage of the viral entry cycle. **Bottom panel**. Same as top but rotated 180°. Cathepsins K and L represent additional host protease targets at another stage of the viral entry cycle. Future efforts and alternative methods on our part may involve discovering effective compounds to exploit this point of intervention in synergy with our other therapeutics. In anticipation of this, we have already constructed models of both of these cathepsins, which exhibit remarkable structural homology with each other. For cathepsin K, 4N8W.pdb {PMID: 25422423} was used as a base from which to construct the model, and 2YJB.pdb {PMID: 21898833} was used for cathepsin L.

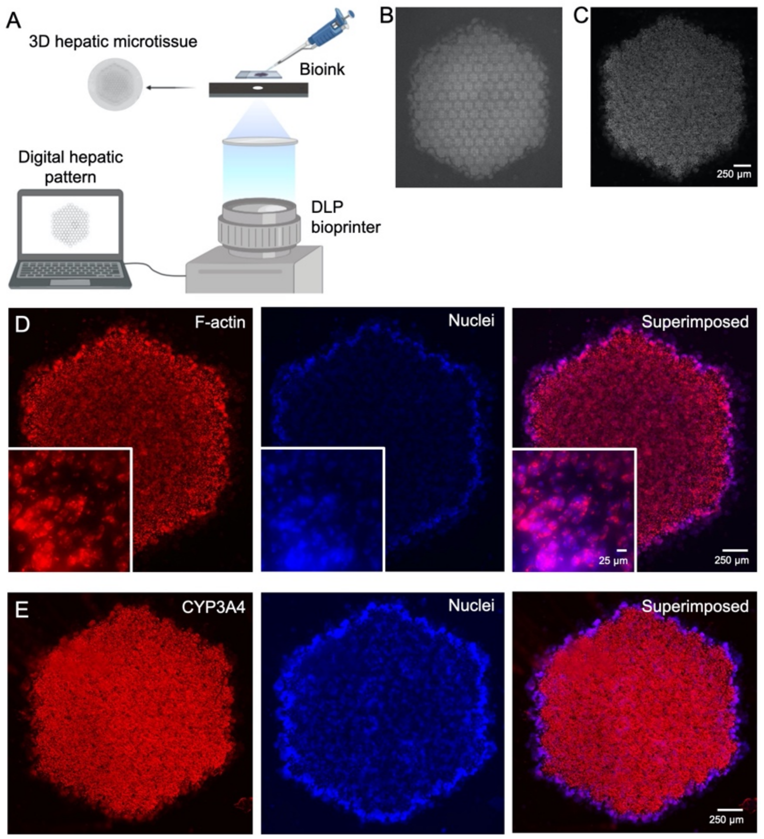

Figure S5. 3D bioprinting of the 3D human hepatic microtissue model and functional characterizations. (A) Schematic diagram showing the DLP-based bioprinting process, where which HepG2-C3A human liver-derived cells were patterned into 3D hexagonal lobule structures in a GelMA hydrogel matrix using the digital mask. (B) Bright-field image showing a bioprinted GelMA hydrogel pattern of hexagonal lobules without cells. (C) Bright-field image showing a bioprinted GelMA hydrogel pattern of hexagonal lobules with HepG2-C3A cells. (D) Fluorescence micrographs showing the expression of F-actin (red) by the cells within the hepatic microtissue at day 7. Nuclei were counterstained in blue. (E) Fluorescence micrographs showing the expression of CYP3A4 (red) by the cells within the hepatic microtissue at day 7. Nuclei were counterstained in blue.
