# Supplementary material for: Attacking COVID-19 Progression Using Multi-Drug Therapy for Synergetic Target Engagement
Source: Biomolecules. 2021 May 23;11(6):787. doi: 10.3390/biom11060787 (PMC8224684; doi:10.3390/biom11060787)
Supplement: Supplementary file 1 [file biomolecules-11-00787-s001.zip › Supplemental-Dataset_No2--repurposeSET.pdf]

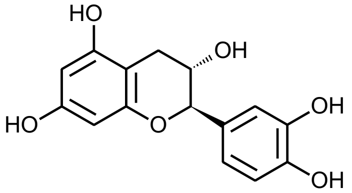<br>title: S4722 (+)-Catechin<br>ENZYME: MPro<br>docking score: -6.73	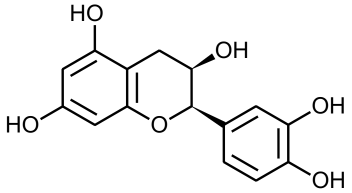<br>title: S4723 (-)Epicatechin<br>ENZYME: MPro<br>docking score: -6.322	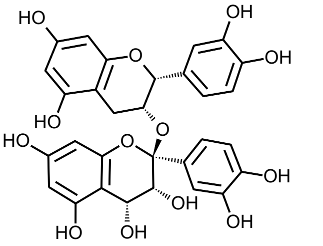<br>title: S5105<br>ENZYME: MPro<br>docking score: -6.19
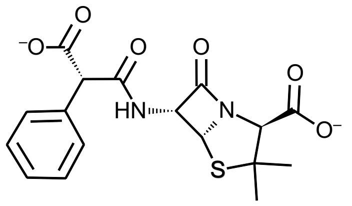<br>title: Carbenicillin disodium<br>ENZYME: MPro<br>docking score: -5.779	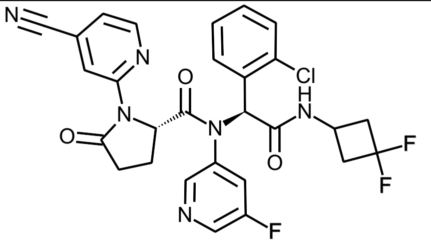<br>title: AG-120 (Ivosidenib)<br>ENZYME: MPro<br>docking score: -5.522	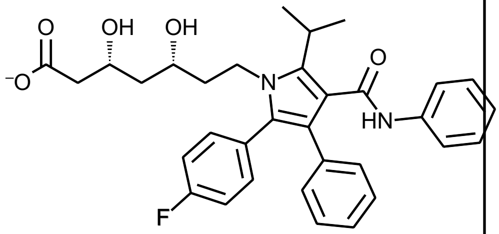<br>title: Atorvastatin calcium<br>ENZYME: MPro<br>docking score: -5.39
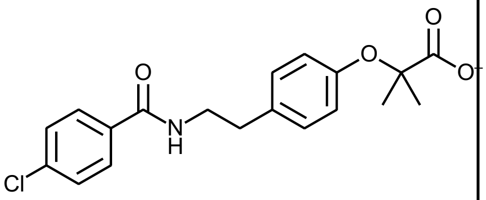<br>title: Bezafibrate<br>ENZYME: MPro<br>docking score: -4.933	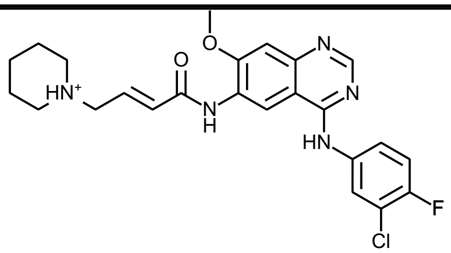<br>title: PF299804<br>ENZYME: MPro<br>docking score: -4.339	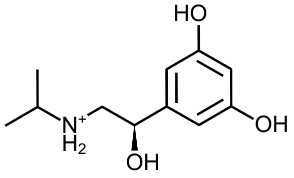<br>title: metaproterenol sulfate(orciprenaline sulfate)<br>ENZYME: Ace2<br>docking score: -8.048
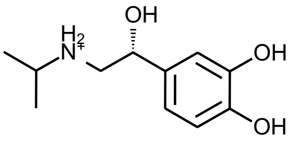<br>title: Isoprenaline hydrochloride<br>ENZYME: Ace2<br>docking score: -7.442	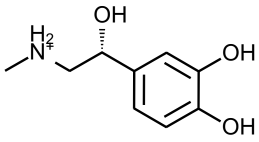<br>title: Epinephrine HCl<br>ENZYME: Ace2<br>docking score: -7.115	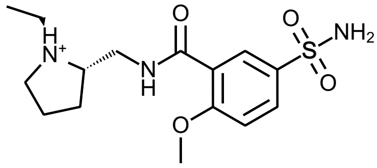<br>title: Levosulpiride<br>ENZYME: Ace2<br>docking score: -6.874

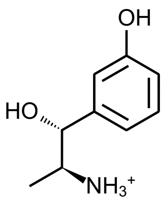

title: metaraminol bitartrate
ENZYME: Ace2
docking score: -6.838

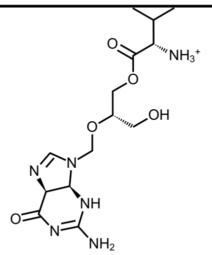

title: valganciclovir hydrochloride
ENZYME: Ace2
docking score: -6.584

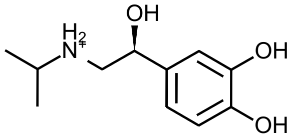

title: Isoprenaline hydrochloride
ENZYME: Ace2
docking score: -6.454

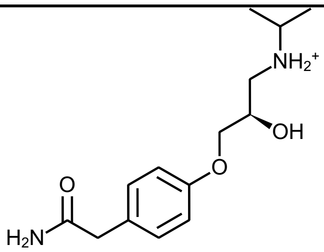

title: S4817 Atenolol
ENZYME: Ace2
docking score: -6.346

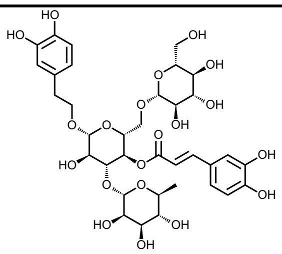

title: S3783 Echinacoside
ENZYME: Ace2
docking score: -6.086

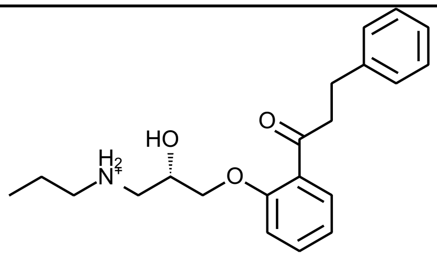

title: Propafenone
ENZYME: Ace2
docking score: -6.04

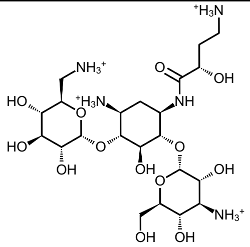

title: Amikacin sulfate
ENZYME: Ace2
docking score: -5.976

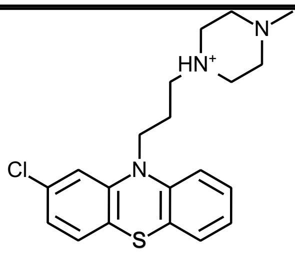

title: Prochlorperazine dimaleate salt
ENZYME: Ace2
docking score: -5.793

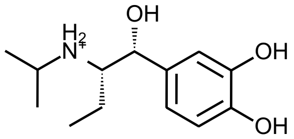

title: isoetharine mesylate
ENZYME: Ace2
docking score: -5.467

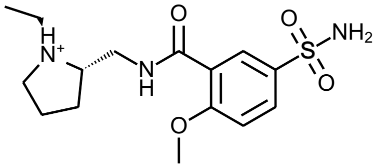

title: Levosulpiride
ENZYME: Ace2
docking score: -6.874

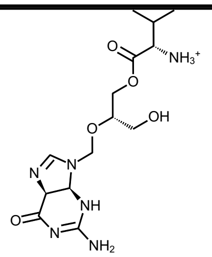

title: valganciclovir hydrochloride
ENZYME: Ace2
docking score: -6.366

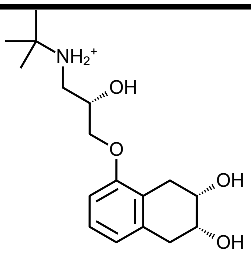

title: S5023 Nadolol
ENZYME: Ace2
docking score: -5.158

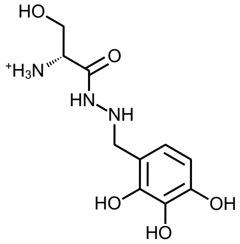

title: Benserazide hydrochloride
ENZYME: Ace2
docking score: -5.928

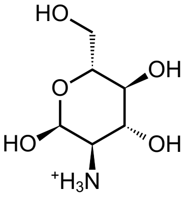

title: S3694 Glucosamine (hydrochloride)
ENZYME: Ace2
docking score: -5.568

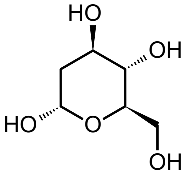

title: S4701 2-Deoxy-D-glucose
ENZYME: Ace2
docking score: -5.181

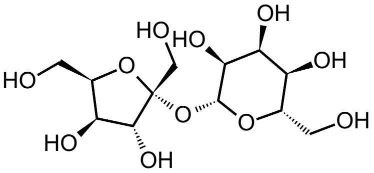

title: Inulin
ENZYME: Ace2
docking score: -5.175

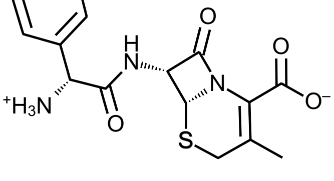

title: CEPHALEXIN (cephalexin)
ENZYME: Ace2
docking score: -5.108

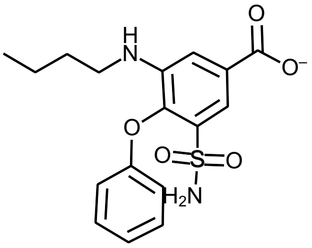

title: Bumetanide
ENZYME: TMPRSS2
docking score: -6.495

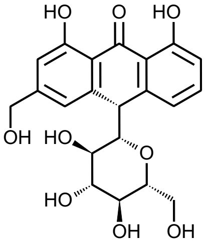

title: Aloin
ENZYME: TMPRSS2
docking score: -6.451

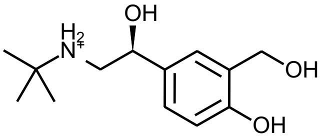

title: Salbutamol sulfate
ENZYME: TMPRSS2
docking score: -6.1

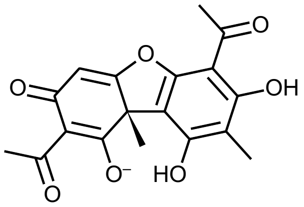

title: S4953 Usnic acid
ENZYME: TMPRSS2
docking score: -5.8

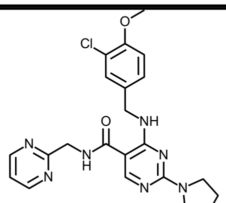

title: Avanafil
ENZYME: TMPRSS2
docking score: -5.616

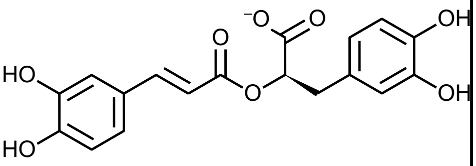

title: S3612 Rosmarinic acid
ENZYME: TMPRSS2
docking score: -5.604

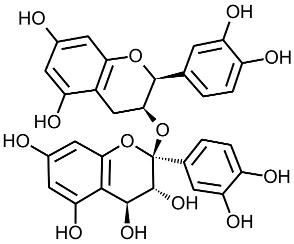

title: S5105
ENZYME: TMPRSS2
docking score: -5.505

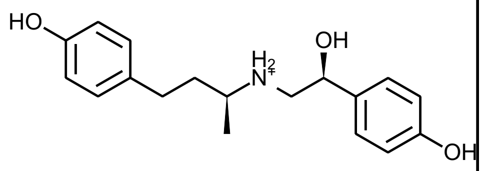

title: ractopamine hydrochloride
ENZYME: TMPRSS2
docking score: -5.218

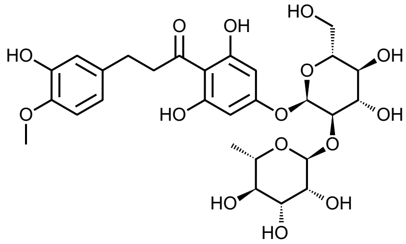

title: Neohesperidin dihydrochalcone
ENZYME: TMPRSS2
docking score: -5.202

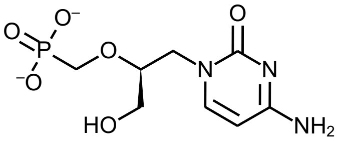

title: Cidofovir
ENZYME: TMPRSS2
docking score: -5.178

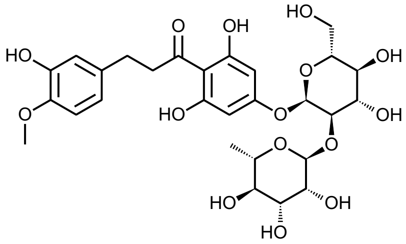

title: Neohesperidin dihydrochalcone
ENZYME: TMPRSS2
docking score: -5.029

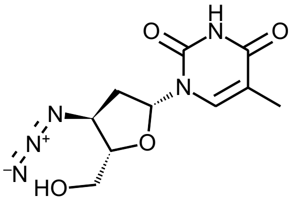

title: Zidovudine
ENZYME: TMPRSS2
docking score: -5.016
