# Supplementary material for: Attacking COVID-19 Progression Using Multi-Drug Therapy for Synergetic Target Engagement
Source: Biomolecules. 2021 May 23;11(6):787. doi: 10.3390/biom11060787 (PMC8224684; doi:10.3390/biom11060787)
Supplement: Supplementary file 1 [file biomolecules-11-00787-s001.zip › Supplemental-Dataset_No3--properties.pdf]

Table S1. Extensive Chemoinformatics Properties and Physiochemical Properties of the Top 310 Compounds Screened for Ace2, TMPRSS2, and MPro

Structure	ENZYME	Docking_score	mol MW	dipole	SASA	FOSA	FISA	PISA	WPSA	volume	donorHB	accptHB	logPo/w	logS	CIlogS	logHERG	PCaco	PMDCK	logKp	logKhsa	PSA	RuleOf5	RuleOf3
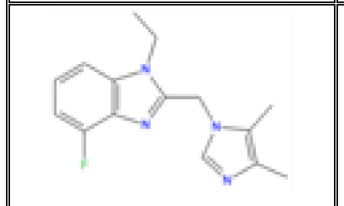	ACE2	-7.938425	272.324	9.772	544.879	283.009	32.461	184.837	44.572	930.925	0	3	4.118	-4.429	-4.304	-4.902	4876.081	4811.036	-1.179	0.391	35.64	0	0
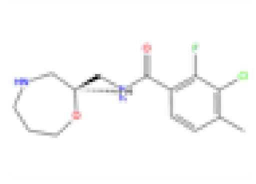	ACE2	-7.936678	300.76	3.719	558.789	319.018	61.931	92.665	85.175	949.235	2	5.7	2.23	-3.124	-2.049	-5.355	639.013	987.633	-4.109	-0.022	50.36	0	0
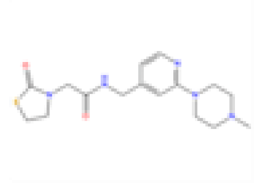	ACE2	-7.935508	349.45	9.231	659.658	411.299	102.348	95.226	50.784	1145.305	1	8.5	1.344	-3.05	-1.884	-4.533	205.553	246.561	-4.749	-0.448	94.08	0	0
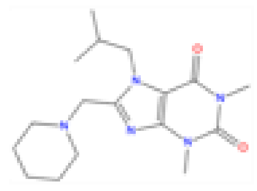	ACE2	-7.933447	333.433	6.286	622.384	529.83	81.265	11.289	0	1120.606	0	7	2.121	-2.475	-2.434	-4.928	418.951	213.714	-4.656	-0.171	65.06	0	0

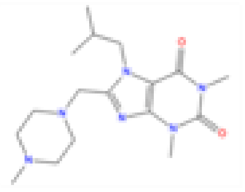	**ACE2**	-7.931598	348.447	6.124	646.555	553.566	83.215	9.774	0	1164.653	0	9	0.892	-0.667	-0.842	-5.814	100.132	50.332	-6.76	-0.578	68.3	0	0
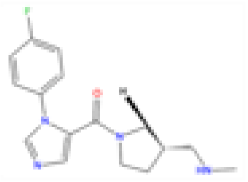	**ACE2**	-7.931379	302.351	3.532	586.418	248.96	77.722	212.94	46.794	1004.576	1	6	2.444	-3.305	-2.644	-6.07	452.645	419.26	-3.976	0.061	50.16	0	0
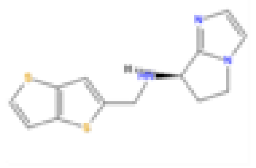	**ACE2**	-7.928268	275.386	5.192	522.396	149.158	28.54	250.81	93.888	870.281	1	3	3.394	-3.42	-3.307	-5.981	1324.829	2424.322	-2.937	0.279	86.33	0	0
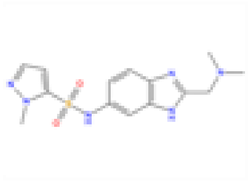	**ACE2**	-7.921568	334.395	7.906	614.473	253.135	141.522	219.816	0	1053.224	2	9	0.853	-2.383	-2.313	-6.304	112.395	51.546	-4.936	-0.415	104.29	0	0

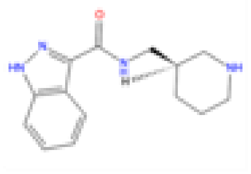	**ACE2**	-7.917339	258.322	5.446	520.712	217.024	125.653	178.034	0	881.416	2	4	1.791	-2.565	-2.356	-5.473	158.938	74.964	-4.983	0.081	69.81	0	0
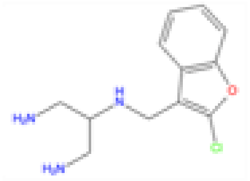	**ACE2**	-7.91669	253.731	4.3	481.915	114.951	113.908	184.493	68.563	813.798	5	4	0.088	1.397	0.164	-6.617	12.776	14.283	-6.422	-0.506	77.21	0	2
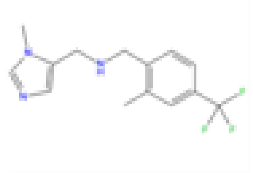	**ACE2**	-7.915507	283.296	3.095	546.024	202.847	38.229	187.17	117.778	919.44	1	3	3.604	-3.555	-3.363	-5.781	1072.197	2606.997	-3.243	0.368	29.85	0	0
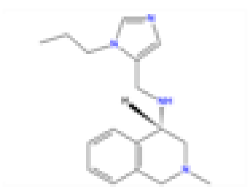	**ACE2**	-7.915427	284.403	4.693	550.797	282.058	43.791	224.948	0	1000.801	1	5	2.398	-1.29	-1.627	-6.023	236.825	127.626	-5.179	0.183	33.09	0	0

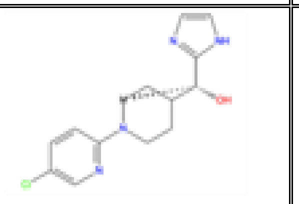	**ACE2**	-7.910061	292.767	7.97	547.317	153.991	94.892	226.364	72.07	930.771	2	4.7	2.901	-4.364	-3.95	-5.163	1247.489	1559.383	-2.183	0.093	65.04	0	0
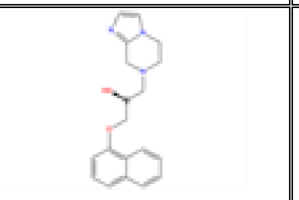	**ACE2**	-7.909158	323.394	2.888	627.736	186.463	61.344	379.929	0	1086.965	1	5.95	3.153	-3.348	-3.323	-7.072	647.248	341.995	-2.798	0.153	50.52	0	0
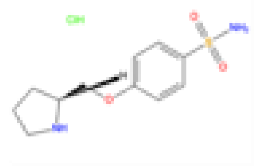	**ACE2**	-7.907147																			89.8		
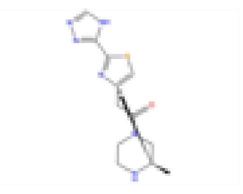	**ACE2**	-7.906252	292.358	11.11	559.083	243.065	150.945	118.12	46.954	939.382	2	8	-0.054	-1.667	-1.119	-4.203	57.609	74.614	-5.756	-0.693	115.04	0	0

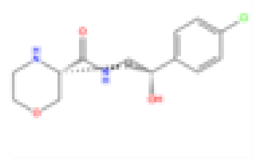	**ACE2**	-7.905742	284.742	5.458	504.984	230.116	79.286	124.069	71.514	880.649	3	7.4	0.38	-0.829	-0.945	-3.419	297.932	551.908	-4.126	-0.792	70.59	0	0
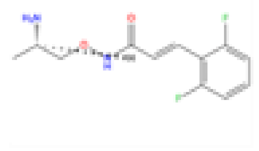	**ACE2**	-7.900215	256.252	7.699	525.185	171.196	123.507	153.165	77.316	859.712	3	5.2	1.218	-1.824	-1.659	-5.698	166.564	209.116	-4.647	-0.385	64.35	0	0
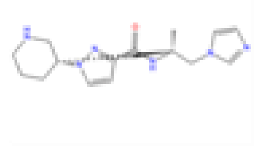	**ACE2**	-7.895666	302.378	5.754	598.413	315.985	110.926	171.501	0	1036.508	2	7.5	1.497	-2.728	-2.051	-5.864	219.224	106.122	-4.638	-0.15	76.77	0	0
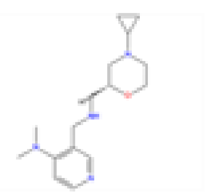	**ACE2**	-7.891833	290.408	3.331	617.599	468.485	38.841	110.273	0	1061.713	1	7.7	1.531	-1.481	-0.283	-6.505	263.856	143.442	-5.492	-0.216	40.63	0	0

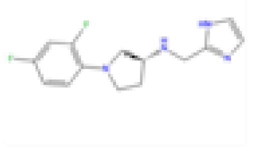	**ACE2**	-7.890993	278.304	7.324	531.383	158.202	57.648	237.625	77.908	895.448	2	4	2.711	-3.085	-2.866	-5.891	701.663	996.996	-3.519	0.112	43.95	0	0
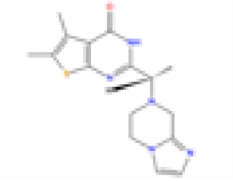	**ACE2**	-7.890335	329.419	5.949	602.159	337.191	100.271	120.072	44.625	1040.367	1	7	1.979	-3.373	-2.974	-5.669	276.652	239.597	-4.815	0	95.05	0	0
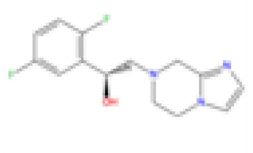	**ACE2**	-7.881457	279.289	4.953	510.805	149.085	57.177	225.398	79.145	873.757	1	5.2	2.258	-2.265	-2.5	-5.513	708.905	1023.97	-3.458	-0.15	41.29	0	0
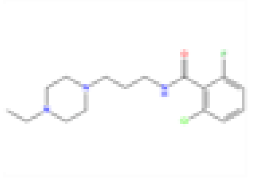	**ACE2**	-7.878207	327.829	6.222	626.962	351.534	40.968	140.929	93.531	1084.179	1	6.5	2.547	-2.321	-1.664	-6.665	251.886	443.859	-5.327	0.037	35.58	0	0

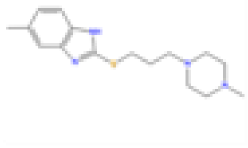	**ACE2**	-7.876119	304.452	5.813	635.876	407.626	46.279	141.559	40.411	1080.104	1	5.5	2.693	-2.773	-1.768	-6.885	224.303	200.361	-5.519	0.26	60.46	0	0
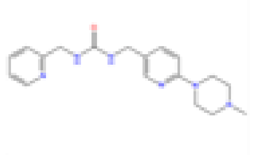	**ACE2**	-7.875927	340.427	6.363	684.886	324.008	88.45	272.429	0	1175.491	2	6.5	2.348	-3.739	-2.669	-5.638	240.159	180.381	-3.869	0.013	73.39	0	0
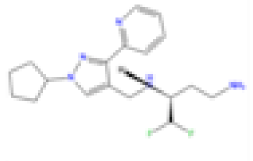	**ACE2**	-7.875737	349.426	4.416	641.986	304.786	74.281	192.482	70.437	1147.227	3	5	2.969	-2.8	-2.703	-6.725	121.7	151.103	-5.76	0.387	68.76	0	0
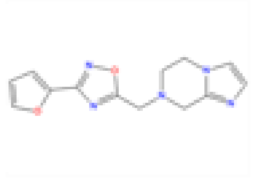	**ACE2**	-7.871089	271.278	5.947	519.363	152.81	93.57	272.983	0	869.545	0	7	0.704	-0.849	-1.483	-6.014	320.238	159.847	-4.153	-0.815	73.12	0	0

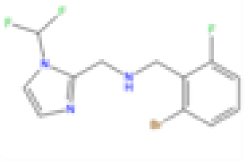	**ACE2**	-7.863935	334.138	2.142	509.836	75.993	23.768	241.955	168.12	856.065	1	3	3.613	-3.357	-4.403	-5.755	1470.297	6920.453	-2.784	0.201	29.85	0	0
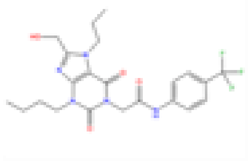	**ACE2**	-7.861967	481.474	9.133	790.791	396.83	127.075	149.314	117.572	1423.417	2	9.2	3.975	-6.111	-6.181	-5.903	617.801	1295.211	-2.375	0.209	111.15	0	1
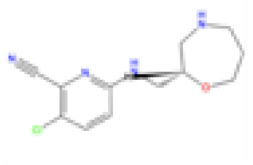	**ACE2**	-7.860086	266.73	9.811	526.892	235.096	111.367	114.63	65.799	871.074	2	6.2	1.184	-3.232	-2.244	-5.44	217.124	240.843	-4.847	-0.329	69.97	0	0
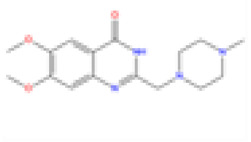	**ACE2**	-7.85665	318.375	3.939	614.248	454.196	92.47	67.582	0	1051.232	1	9	0.506	-1.118	-0.751	-6.316	81.809	40.456	-6.727	-0.457	70.69	0	0

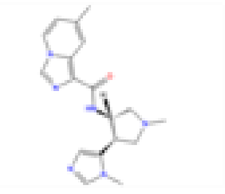	**ACE2**	-7.856272	338.411	10.408	636.211	309.067	81.807	245.337	0	1124.433	1	7.5	2.364	-3.578	-2.95	-6.309	414.02	210.996	-4.034	0.118	67.98	0	0
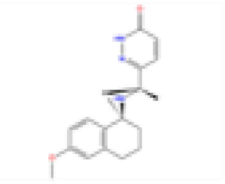	**ACE2**	-7.855813	299.372	4.632	569.586	295.145	116.651	157.79	0	997.834	2	4.25	2.454	-3.166	-3.192	-5.473	193.465	92.711	-4.792	0.296	67.01	0	0
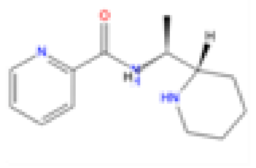	**ACE2**	-7.85541	233.313	5.069	513.903	269.391	68.079	176.432	0	858.984	2	5	1.683	-2.202	-1.387	-5.521	558.734	291.735	-3.927	-0.126	54.02	0	0
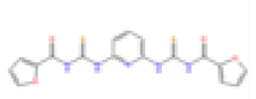	**ACE2**	-7.848658	415.441	16.725	692.807	0	144.333	445.163	103.31	1188.42	2	6	3.986	-6.424	-6.161	-7.163	423.826	719.974	-2.227	0.455	185.61	0	1

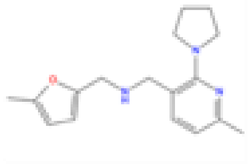	**ACE2**	-7.84725	285.388	2.357	571.612	376.031	12.28	183.301	0	1020.663	1	3.5	3.824	-3.556	-3.257	-5.435	1889.488	1088.742	-2.779	0.556	41.3	0	0
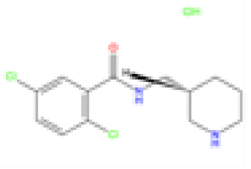	**ACE2**	-7.847098																			41.13		
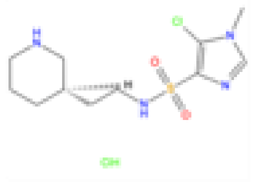	**ACE2**	-7.845325																			84.4		
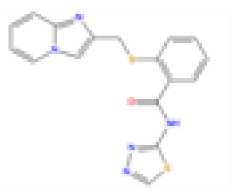	**ACE2**	-7.843258	367.443	5.873	596.154	43.878	70.811	393.663	87.801	1071.28	1	6.5	3.608	-4.368	-5.288	-5.847	2110.551	3357.039	-0.958	0.065	126.24	0	0

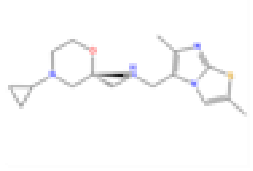	**ACE2**	-7.838944	320.452	6.234	610.877	488.308	31.854	42.526	48.189	1080.76	1	6.7	2.229	-2.1	-1.367	-5.877	307.346	310.653	-5.698	0.084	70.56	0	0
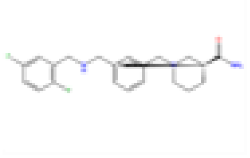	**ACE2**	-7.835397	373.445	4.832	635.405	220.03	82.851	267.927	64.597	1186.687	3	6	2.248	-1.504	-2.303	-5.264	66.778	114.668	-5.556	-0.006	58.36	0	1
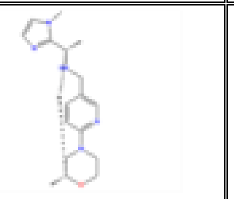	**ACE2**	-7.83441	315.417	4.25	617.372	402.259	40.969	174.143	0	1086.584	1	6.2	2.904	-3.402	-2.884	-5.873	1009.929	553.181	-3.34	0.195	55.21	0	0
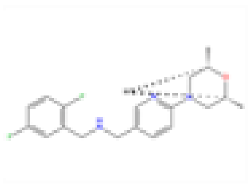	**ACE2**	-7.830545	347.407	2.247	671.348	331.326	25.891	231.222	82.909	1154.441	1	4.7	4.429	-5.267	-4.168	-6.715	1403.718	2246.975	-2.861	0.661	37.39	0	0

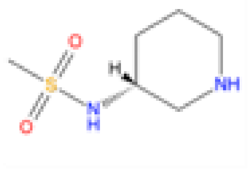	**ACE2**	-7.829874	178.249	5.142	391.406	279.732	111.674	0	0	624.607	2	6	-0.49	-0.311	0.181	-3.83	215.673	104.266	-5.448	-0.688	66.58	0	0
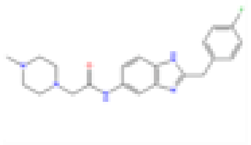	**ACE2**	-7.82796	381.452	2.196	719.04	303.61	87.726	280.892	46.812	1253.44	2	8	2.521	-3.488	-2.682	-7.853	90.738	81.666	-5.792	0.217	64.26	0	0
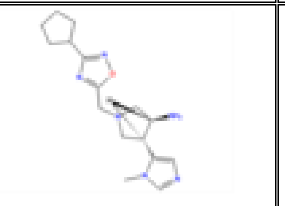	**ACE2**	-7.82452	316.405	8.079	612.288	387.85	136.156	88.282	0	1072.757	2	7.5	0.917	-1.894	-1.244	-6.193	31.516	14.428	-7.555	-0.02	86	0	0
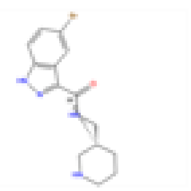	**ACE2**	-7.822889	337.218	6.949	545.734	217.053	125.675	125.936	77.07	930.472	2	4	2.322	-3.325	-3.905	-5.373	158.864	198.072	-5.167	0.202	69.81	0	0

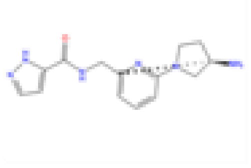	**ACE2**	-7.821383	286.336	4.655	580.953	197.691	174.436	208.826	0	968.845	4	6	0.921	-2.672	-2.227	-6.246	54.78	23.705	-5.677	-0.188	99.93	0	0
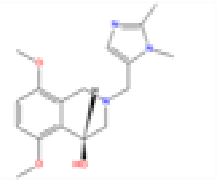	**ACE2**	-7.821377	317.387	7.661	606.538	412.557	69.616	124.366	0	1062.354	1	6.7	2.29	-2.872	-2.674	-5.6	540.3	281.346	-3.947	-0.008	59.75	0	1
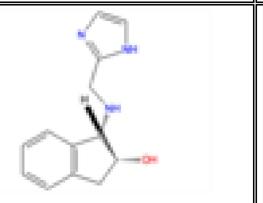	**ACE2**	-7.818057	229.281	2.258	485.61	112.497	93.632	279.482	0	799.975	3	4.7	1.221	-1.559	-1.612	-5.885	319.807	159.615	-3.939	-0.314	60.94	0	0
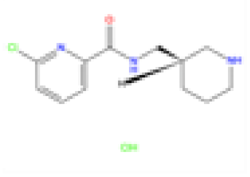	**ACE2**	-7.817936																			54.02		

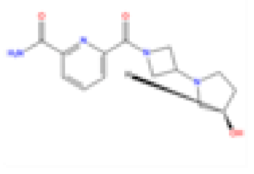	**ACE2**	-7.815683	290.321	5.526	553.428	228.138	202.254	123.036	0	945.322	3	9.2	-0.423	-1.69	-0.858	-5.406	29.842	12.294	-6.492	-0.574	99.76	0	0
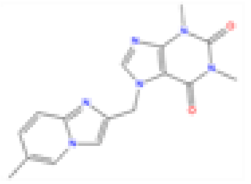	**ACE2**	-7.813326	324.341	9.785	598.382	282.669	100.585	215.128	0	1028.889	0	6.5	2.398	-3.828	-4.025	-5.411	1101.678	549.296	-2.423	-0.219	79.64	0	0
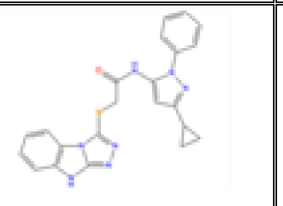	**MPRO**	-7.809379	429.498	7.068	744.24	171.578	147.031	391.51	34.121	1310.612	2	5.5	4.602	-7.18	-6.859	-6.988	399.584	282.261	-2.466	0.865	118.72	0	1
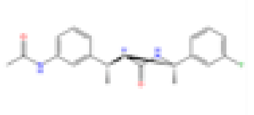	**MPRO**	-7.802171	343.4	2.69	604.347	277.475	84.347	228.2	14.326	1103.452	3	4.5	3.087	-3.964	-4.295	-3.6	1076.198	965.432	-1.79	0.088	70.23	0	0

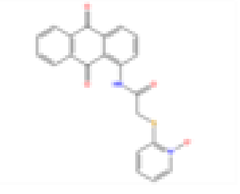	**MPRO**	-7.802135	390.413	11.368	662.357	26.763	166.592	439.872	29.13	1155.438	0	6.5	3.141	-4.841	-5.548	-6.802	260.679	167.033	-2.656	0.064	115.48	0	0
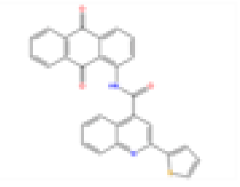	**MPRO**	-7.800731	460.506	5.016	753.309	0	113.988	595.742	43.579	1348.951	0	6.5	5.005	-6.953	-7.576	-7.872	822.157	693.669	-1.33	0.71	104.37	1	1
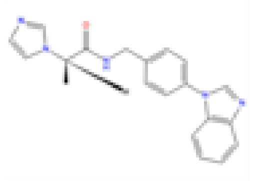	**ACE2**	-7.800908	345.403	8.746	638.095	134.667	93.068	410.36	0	1138.093	1	6	3.126	-4.5	-4.403	-4.927	954.948	655.922	-1.405	0.048	64.74	0	0
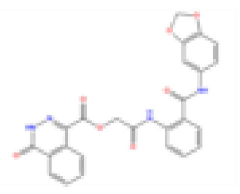	**MPRO**	-7.796552	486.44	10.331	754.515	111.825	221.391	421.299	0	1373.452	1	8.5	3.277	-5.812	-7.117	-6.907	78.785	31.735	-3.54	0.381	148.71	1	1

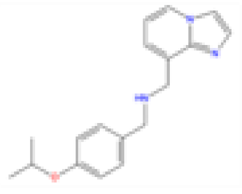	**ACE2**	-7.797499	295.383	2.583	616.302	242.961	30.087	343.254	0	1052.913	1	3.75	3.975	-3.943	-3.563	-6.935	1280.802	715.163	-2.351	0.496	39.08	0	0
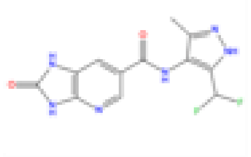	**MPRO**	-7.796011	308.247	7.028	526.269	106.344	243.929	99.542	76.454	875.269	4	6.5	0.456	-3.657	-3.654	-4.582	48.164	48.903	-5.472	-0.333	119.32	0	0
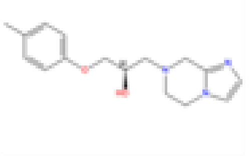	**ACE2**	-7.796023	287.361	2.921	596.042	276.439	64.177	255.426	0	1011.002	1	5.95	2.464	-2.797	-2.374	-6.432	608.429	319.879	-3.289	-0.045	50.52	0	0
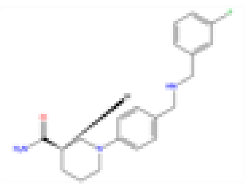	**ACE2**	-7.795565	341.427	2.158	582.793	218.697	124.457	193.806	45.833	1083.165	3	5	2.074	-1.853	-3.075	-3.827	82.233	137.463	-4.713	-0.019	58.36	0	0

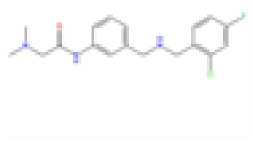	**ACE2**	-7.794252	349.835	6.238	671.614	250.845	57.184	257.632	105.953	1148.417	2	6	3.082	-3.146	-2.525	-7.616	176.776	354.058	-5.119	0.228	44.37	0	0
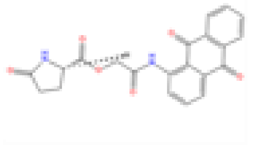	**MPRO**	-7.792261	392.367	7.238	650.912	137.106	246.041	267.764	0	1159.617	0.25	9.25	0.581	-2.106	-3.656	-4.346	19.375	17.737	-4.727	-0.719	118.64	0	1
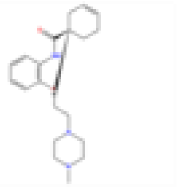	**ACE2**	-7.790159	343.468	5.733	687.292	408.654	45.767	232.871	0	1211.439	1	7.25	2.738	-2.682	-1.781	-7.337	226.824	121.811	-5.092	0.213	44.81	0	1
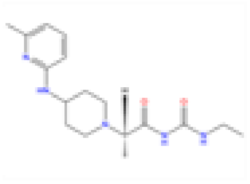	**ACE2**	-7.789715	333.433	8.409	686.614	455.28	98.993	132.341	0	1177.328	2	6	2.32	-3.616	-2.29	-4.989	175.776	140.644	-4.46	0.063	86.36	0	0

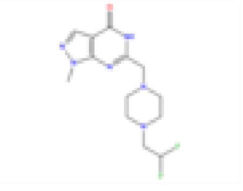	**ACE2**	-7.78928	312.322	5.161	579.858	317.827	112.376	62.363	87.292	983.218	1	8.5	0.495	-1.272	-0.769	-6.119	52.971	76.056	-7.208	-0.478	70.05	0	0
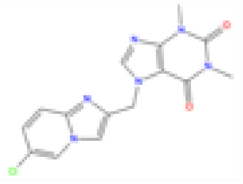	**ACE2**	-7.787989	344.76	7.552	588.853	194.036	98.623	224.398	71.796	1011.681	0	6.5	2.584	-3.96	-4.458	-5.391	1149.905	1423.039	-2.355	-0.269	79.64	0	0
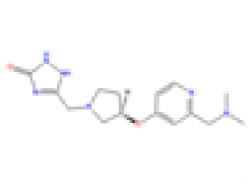	**ACE2**	-7.785194	318.378	10.617	628.678	346.511	173.855	108.312	0	1071.896	2	11.25	-0.798	-0.551	-0.034	-6.645	13.837	5.926	-7.891	-0.806	90.14	0	1
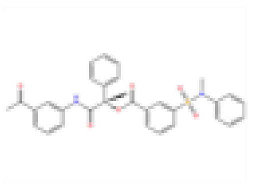	**MPRO**	-7.784198	542.605	8.531	914.847	151.081	178.885	584.056	0.825	1663.153	1	11	4.471	-7.133	-7.224	-8.491	199.311	87.447	-1.895	0.458	118.23	1	1

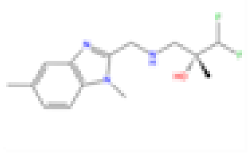	**ACE2**	-7.784154	283.32	5.234	537.948	278.944	63.875	120.085	75.044	929.309	2	3.75	2.833	-2.924	-2.889	-5.159	612.456	830.184	-3.856	0.158	50.08	0	0
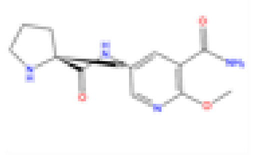	**ACE2**	-7.783586	264.283	8.864	527.684	267.918	181.881	77.885	0	875.299	4	7.5	-0.28	-1.654	-1.127	-5.241	46.561	19.885	-6.276	-0.523	106.34	0	0
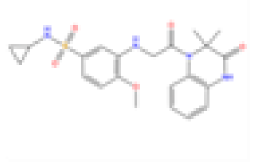	**MPRO**	-7.783271	458.531	7.585	675.328	297.888	136.892	239.068	1.48	1307.122	3	11.75	1.876	-3.537	-4.531	-5	498.6	237.554	-2.528	-0.275	125.22	0	1
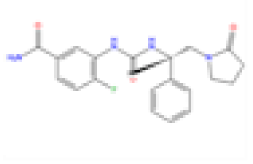	**MPRO**	-7.779582	384.409	12.546	674.308	174.154	201.658	265.462	33.034	1195.182	4	7.5	1.017	-3.071	-3.434	-3.16	45.203	76.695	-3.725	-0.591	104.53	0	0

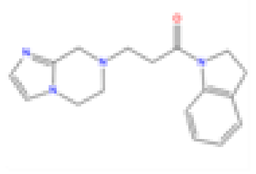	**ACE2**	-7.778121	296.371	4.355	585.58	249.575	67.651	268.354	0	1006.146	0	6.5	2.013	-2.203	-2.059	-6.301	563.987	294.7	-3.596	-0.327	41.37	0	0
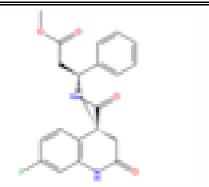	**MPRO**	-7.771538	370.379	5.287	629.8	170.768	141.25	270.892	46.891	1131.779	1	6	2.772	-4.338	-4.536	-4.136	322.834	380.068	-2.688	-0.007	84.5	0	0
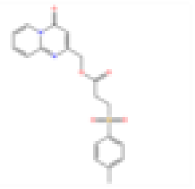	**MPRO**	-7.770157	386.422	10.037	702.02	181.112	192.741	327.14	1.026	1209.265	0	10	1.367	-3.312	-3.6	-6.621	147.276	63.215	-3.343	-0.801	103.71	0	0
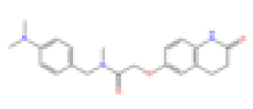	**MPRO**	-7.769056	367.447	7.494	712.043	367.483	124.793	219.768	0	1241.885	1	7.25	2.786	-4.571	-3.98	-4.689	376.848	310.223	-2.469	-0.036	61.88	0	0

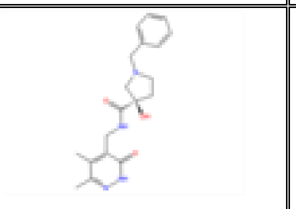	**MPRO**	-7.768783	356.424	9.4	559.315	292.223	127.695	139.397	0	1085.211	2	6.25	1.559	-1.305	-3.01	-2.955	96.565	71.437	-4.868	-0.164	98.32	0	0
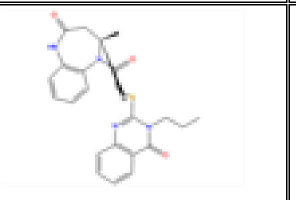	**MPRO**	-7.768639	436.528	10.148	697.187	282.049	110.368	281.773	22.996	1307.809	1	9.5	3.159	-4.701	-5.094	-5.634	889.765	582.769	-2.081	0.067	109.6	0	0
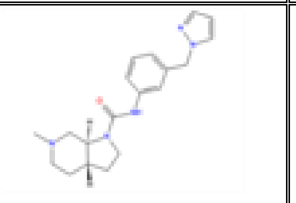	**MPRO**	-7.762516	339.439	6.879	656.341	329.453	61.906	264.982	0	1150.589	1	5.5	2.924	-3.942	-2.978	-5.237	468.603	337.495	-3.502	0.217	53.4	0	0
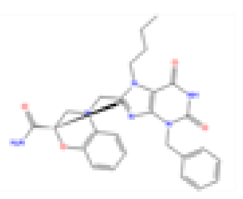	**MPRO**	-7.759647	488.545	5.389	694.663	249.863	207.423	237.377	0	1379.723	3	9.25	2.132	-2.855	-6.072	-3.509	47.061	44.128	-3.738	-0.066	128.24	0	0

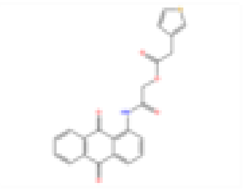	**MPRO**	-7.757383	405.424	6.482	619.84	87.295	160.604	340.454	31.487	1149.737	0	7.5	2.551	-3.359	-5.343	-5.481	297.091	198.195	-2.8	-0.263	117.78	0	0
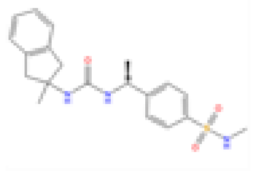	**MPRO**	-7.75727	387.496	7.587	650.312	265.245	165.626	217.858	1.583	1205.351	3	6.5	2.351	-3.764	-4.446	-3.749	145.45	120.727	-3.324	0.041	95.68	0	0
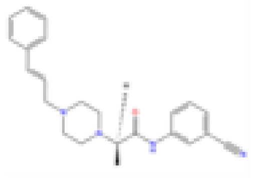	**MPRO**	-7.756551	374.485	4.321	760.238	260.495	104.873	394.87	0	1316.145	1	8	2.835	-4.507	-3.357	-8.686	62.4	30.189	-5.514	0.273	59.37	0	0
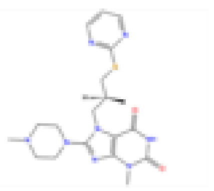	**ACE2**	-7.751422	430.527	8.736	711.981	399.671	142.644	139.978	29.688	1304.677	1	9.5	2.376	-3.763	-4.096	-5.984	109.675	73.002	-5.238	0.046	130.24	0	0

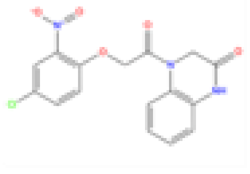	**MPRO**	-7.749496	361.741	7.181	574.598	20.995	201.398	280.617	71.589	1002.489	1	7.25	1.772	-3.83	-4.572	-5.41	121.91	125.496	-3.859	-0.218	101.78	0	0
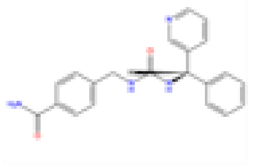	**MPRO**	-7.749195	360.415	9.576	583.777	53.382	175.571	354.824	0	1100.269	4	6	1.838	-2.878	-4.375	-3.744	142.862	93.584	-2.929	-0.217	97.11	0	1
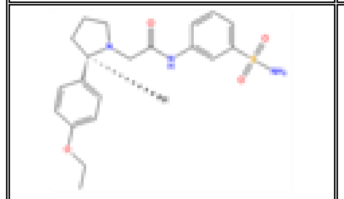	**MPRO**	-7.748233	403.495	5.632	746.619	303.685	179.365	261.749	1.819	1288.501	3	9.75	1.602	-3.966	-3.24	-7.277	49.191	21.591	-5.294	-0.12	110.11	0	0
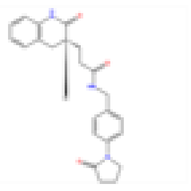	**MPRO**	-7.747528	391.469	10.529	740.772	294.389	158.077	288.306	0	1294.132	2	8	2.538	-5.302	-4.267	-5.169	216.158	141.42	-2.937	-0.006	78.51	0	0

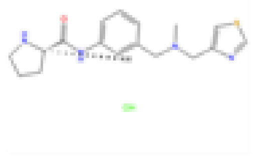	**ACE2**	-7.740865																			85.5		
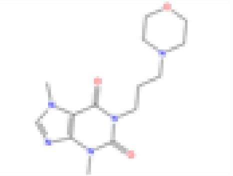	**ACE2**	-7.740514	307.352	5.373	554.897	418.338	77.38	59.179	0	981.695	0	8.7	0.47	-0.306	-1.156	-4.792	456.043	234.237	-4.416	-1.029	74.29	0	0
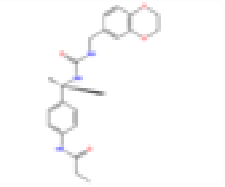	**MPRO**	-7.740255	383.446	10.161	593.829	343.692	89.354	160.783	0	1137.518	3	6	2.615	-3.194	-4.668	-2.746	953.915	716.011	-2.024	-0.057	88.69	0	0
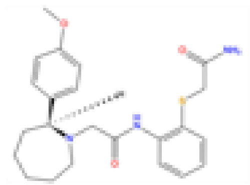	**MPRO**	-7.739322	427.56	7.751	726.557	329.669	102.613	262.358	31.917	1341.482	3	8.25	2.568	-3.655	-3.17	-5.215	193.632	193.133	-3.877	-0.015	109.96	0	1

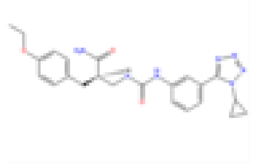	**MPRO**	-7.739139	449.511	15.893	841.599	353.054	233.968	254.577	0	1469.136	3	7.25	2.539	-5.442	-4.866	-4.162	20.767	23.585	-4.167	0.053	137.05	0	1
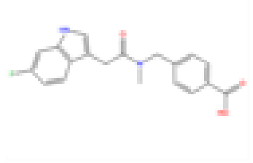	**MPRO**	-7.737004	340.353	6.904	614.156	118.226	161.242	288.121	46.567	1066.502	2	5	3.018	-4.248	-4.338	-2.605	49.446	68.072	-2.996	-0.189	73.4	0	0
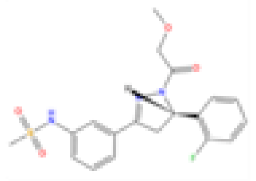	**MPRO**	-7.733552	405.443	10.121	666.025	249.499	112.969	267.325	36.231	1207.998	1	9.7	2.468	-4.32	-4.454	-5.661	840.653	647.657	-2.276	-0.193	96.45	0	0
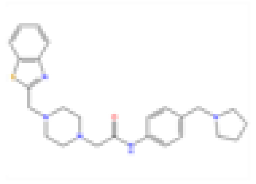	**ACE2**	-7.733448	449.613	6.37	833.552	419.838	59.235	309.305	45.174	1483.515	1	10	2.879	-2.973	-2.218	-9.155	42.157	38.646	-4.975	0.288	79.95	0	1

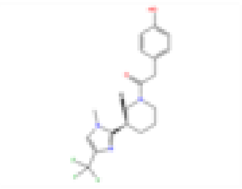	**MPRO**	-7.722702	367.37	9.205	641.662	235.1	89.515	192.709	124.338	1126.465	1	5.25	3.605	-5.322	-4.638	-4.055	938.696	3422.959	-2.202	0.205	58.36	0	0
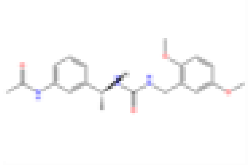	**MPRO**	-7.721882	371.435	5.18	704.332	396.58	89.192	218.559	0	1247.181	3	6	3.278	-5.059	-4.325	-4.508	1074.599	718.75	-1.721	0.1	88.69	0	0
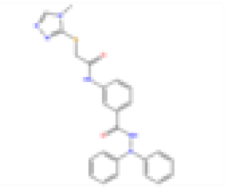	**MPRO**	-7.719529	458.537	5.482	785.56	99.541	127.197	520.976	37.845	1416.444	2	7.5	4.757	-6.586	-6.866	-7.634	616.158	472.443	-1.26	0.6	117.45	0	1
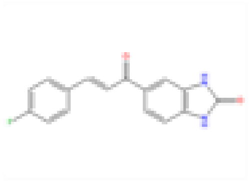	**MPRO**	-7.719031	282.273	1.681	538.212	13.254	172.139	305.989	46.83	890.268	2	4	2.367	-4.122	-4.191	-5.717	230.945	183.194	-3.23	0.049	65.72	0	0

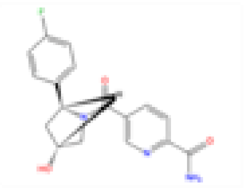	**MPRO**	-7.718797	329.33	6.441	577.401	93.792	209.559	227.235	46.814	1008.441	3	8.2	1.067	-3.835	-3.463	-5.168	102.012	75.73	-4.293	-0.263	96.52	0	0
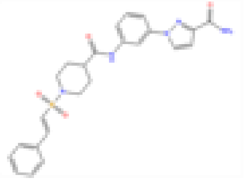	**MPRO**	-7.715121	479.553	7.166	825.841	155.654	232.44	436.998	0.749	1467.432	3	10.5	2.738	-6.392	-5.754	-7.561	61.897	24.683	-3.688	0.215	135.77	0	1
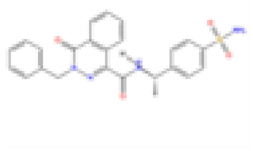	**MPRO**	-7.71278	462.522	7.779	767.028	132.965	207.575	424.729	1.759	1386.042	3	9	3.024	-5.735	-6.248	-7.056	106.529	44.957	-3.177	0.264	132.53	0	1
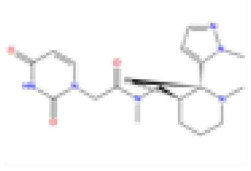	**MPRO**	-7.712632	374.442	10.862	585.166	340.07	147.588	97.508	0	1114.508	1	9.5	0.3	-1.334	-2.041	-3.155	81.011	44.671	-5.575	-0.66	96.23	0	0

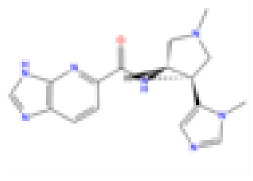	**ACE2**	-7.71168	325.372	8.665	607.257	228.571	150.383	228.303	0	1053.558	2	8.5	1.007	-2.905	-2.366	-6.185	92.623	41.819	-5.357	-0.193	91.73	0	0
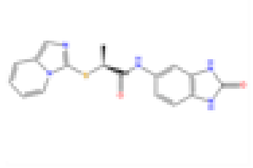	**MPRO**	-7.711375	353.398	12.99	595.916	82.738	181.553	297.497	34.128	1041.951	3	6	2.209	-4.404	-4.977	-5.635	188.034	124.98	-3.433	0.07	120.87	0	0
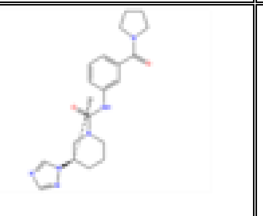	**MPRO**	-7.708583	368.438	6.662	665.965	331.611	109.049	225.304	0	1192.979	1	8	2.299	-4.794	-3.608	-4.196	763.657	449.829	-2.544	-0.129	83.36	0	0
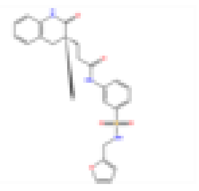	**MPRO**	-7.706308	453.512	10.696	793.021	126.488	207.998	456.744	1.792	1382.104	3	10	2.613	-5.693	-5.514	-7.648	105.55	44.529	-2.976	0.009	125.89	0	1

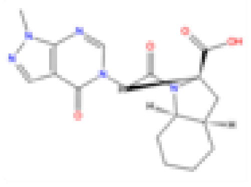	**MPRO**	-7.705968	359.384	9.903	608.123	319.976	194.655	93.493	0	1090.383	1	10	0.621	-2.35	-2.463	-1.345	23.755	17.194	-4.49	-0.973	110.32	0	0
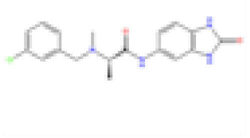	**MPRO**	-7.705485	358.827	10.499	655.567	162.237	151.169	270.574	71.587	1121.764	3	6.5	2.327	-4.027	-3.681	-6.842	91.047	101.268	-4.935	0.114	80.99	0	0
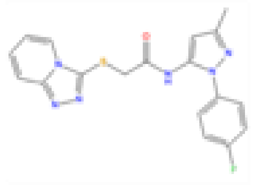	**MPRO**	-7.703882	382.414	6.119	641.936	133.215	116.833	316.852	75.037	1159.476	1	5.5	4.11	-5.633	-5.989	-5.77	772.63	964.522	-2.173	0.53	102.93	0	0
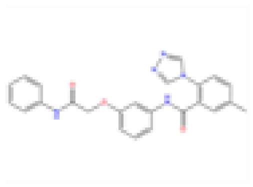	**MPRO**	-7.70219	427.462	10.385	772.394	123.807	154.469	494.118	0	1364.896	2	7.75	3.965	-6.435	-6.152	-7.603	339.683	153.991	-2.05	0.498	98.14	0	1

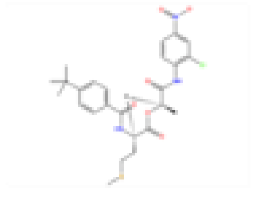	**MPRO**	-7.697784	536.041	9.034	912.128	438.627	161.984	209.519	101.998	1656.122	1.25	7.75	5.885	-8.546	-7.72	-6.703	288.274	466.885	-2.711	1.067	152.94	2	1
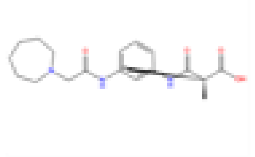	**ACE2**	-7.696227	347.413	8.401	659.725	352.725	173.678	133.322	0	1163.224	1	7	0.715	-4.181	-2.415	-4.148	14.106	6.957	-5.737	-0.018	98.74	0	1
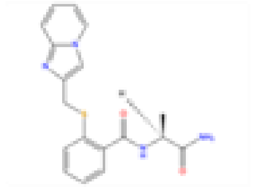	**ACE2**	-7.695058	354.426	5.916	637.721	102.588	151.648	357.405	26.081	1119.377	2.25	6.25	2.374	-3.588	-4.144	-4.814	191.707	228.706	-2.479	-0.159	115.31	0	0
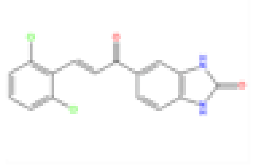	**MPRO**	-7.693111	333.173	4.637	561.182	2.457	172.014	275.772	110.939	946.309	2	4	2.924	-4.794	-5.197	-5.587	231.575	412.458	-3.334	0.193	65.72	0	0

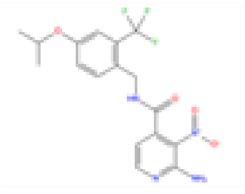	**MPRO**	-7.691484	398.341	7.844	636.227	209.188	161.294	170.393	95.352	1134.016	3	5.75	3.135	-4.899	-5.58	-5.15	292.651	436.391	-3.22	0.199	120.38	0	0
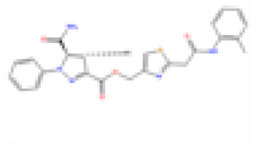	**MPRO**	-7.69133	477.537	13.002	851.627	205.432	196.557	400.495	49.144	1491.333	3	9.5	3.026	-5.985	-5.583	-6.254	69.815	105.998	-3.059	0.071	155.22	0	2
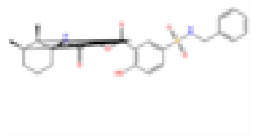	**MPRO**	-7.691032	474.571	7.282	876.721	348.263	220.409	306.266	1.784	1549.425	2	8.75	3.383	-6.658	-5.37	-5.848	53.378	33.218	-3.639	0.314	130.18	0	1
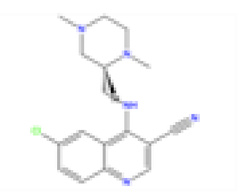	**ACE2**	-7.690945	329.831	5.461	606.775	270.368	87.972	177.043	71.392	1060.769	1	7.5	1.628	-2.844	-2.554	-6.596	90.253	110.705	-6.258	-0.127	55.19	0	0

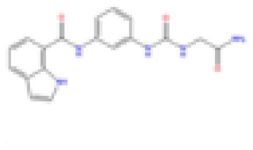	**MPRO**	-7.68726	351.364	6.062	652.633	49.503	232.715	370.415	0	1102.321	4.25	5.25	0.963	-3.037	-3.597	-3.936	21.062	24.292	-4.024	-0.508	129.11	1	1
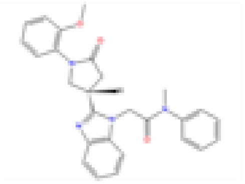	**MPRO**	-7.687013	454.527	6.284	796.525	240.066	78.623	477.835	0	1451.944	0	8.25	4.765	-6.34	-6.263	-7.395	1779.568	922.381	-0.901	0.456	67.67	0	1
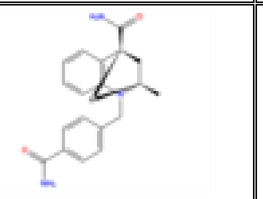	**MPRO**	-7.686286	323.394	3.516	582.998	145.747	207.06	230.192	0	1037.208	4	6	1.064	-2.728	-3.368	-3.683	55.583	44.508	-4.141	-0.314	89.42	0	1
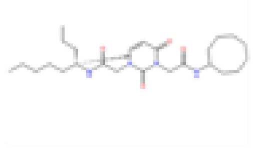	**MPRO**	-7.685229	462.631	9.017	908.905	721.146	118.182	69.578	0	1634.911	2	8.5	3.821	-6.472	-3.74	-3.347	411.756	362.607	-2.3	0.142	102.2	0	1

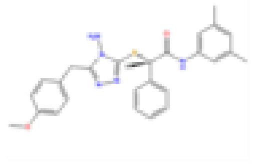	**MPRO**	-7.684616	473.592	12.224	686.495	235.352	95.619	323.145	32.379	1372.545	3	7.25	3.944	-3.51	-6.131	-5.902	306.229	229.129	-3.342	0.52	120.36	0	1
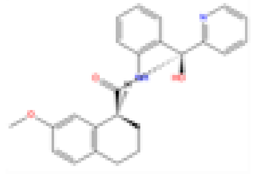	**MPRO**	-7.680547	388.465	5.282	658.355	208.447	81.163	368.745	0	1231.156	2	5.95	4.267	-5.075	-5.742	-5.818	1683.579	868.723	-1.14	0.53	71.45	0	1
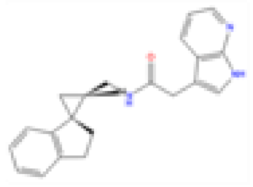	**MPRO**	-7.679692	331.416	4.881	612.951	198.585	100.407	313.959	0	1094.571	2	3.5	3.537	-4.607	-4.496	-4.276	731.651	551.609	-1.88	0.348	57.78	0	0
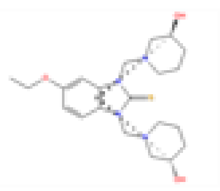	**ACE2**	-7.6796	420.569	8.429	751.077	517.755	106.653	77.15	49.52	1350.484	2	10.65	1.696	-2.55	-2.046	-6.804	60.022	54.06	-6.57	-0.15	98.12	0	0

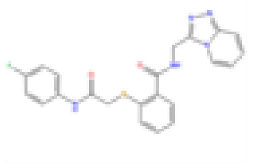	**MPRO**	-7.678648	435.475	12.089	601.136	68.45	115.344	338.115	79.227	1174.825	2	7.5	3.39	-3.848	-6.286	-4.832	798.158	1053.236	-1.782	0.121	114.21	0	0
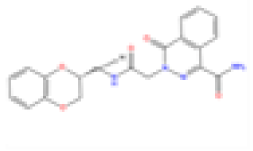	**MPRO**	-7.677514	394.386	9.404	592.3	148.222	183.342	260.736	0	1110.146	3	8.5	1.012	-2.506	-4.232	-3.417	106.826	77.901	-3.5	-0.443	125.54	0	0
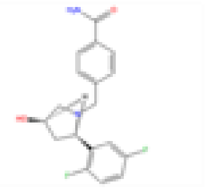	**MPRO**	-7.676844	332.349	6.069	576.207	124.799	150.994	236.398	64.017	1016.877	3	6.2	1.786	-2.957	-3.142	-5.833	91.396	92.427	-5.148	-0.004	66.56	0	0
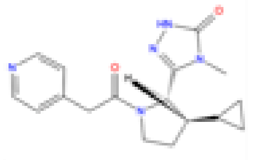	**MPRO**	-7.675123	327.385	8.976	606.39	284.648	161.383	160.358	0	1072.389	1	7.5	1.333	-3.531	-2.768	-3.583	191.761	130.805	-3.737	-0.33	83.88	0	0

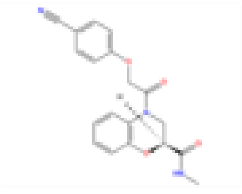	**MPRO**	-7.673447	351.361	2.092	656.987	156.304	162.728	337.955	0	1124.803	1	8.5	1.412	-4.302	-4.168	-5.068	155.036	126.719	-2.848	-0.548	91.66	0	0
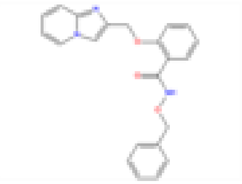	**ACE2**	-7.671655	373.41	3.101	700.931	71.285	70.489	559.157	0	1220.85	1	6.45	4.394	-5.501	-5.488	-7.627	2125.441	1117.593	-0.177	0.354	65.38	0	0
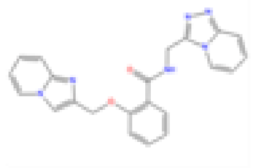	**ACE2**	-7.670744	398.423	10.532	620.866	80.4	88.23	452.236	0	1178.466	1	6.75	3.797	-4.157	-6.051	-5.808	1442.832	735.264	-0.976	0.266	86.86	0	0
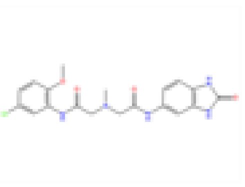	**ACE2**	-7.666898	417.851	12.393	744.554	229.667	205.403	237.993	71.491	1268.058	4	9.75	1.385	-4.186	-3.596	-7.261	27.859	28.122	-5.857	-0.209	119.32	0	0

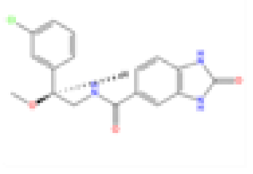	**MPRO**	-7.666192	345.785	2.153	622.361	125.568	166.817	258.391	71.585	1062.755	3	6.2	2.496	-4.783	-4.583	-5.851	259.404	283.836	-3.204	0.026	86.98	0	0
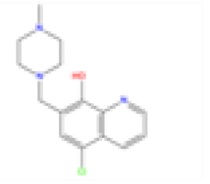	**ACE2**	-7.666059	291.78	1.81	542.156	251.874	58.698	168.294	63.289	934.36	1	5.75	1.739	-1.487	-1.484	-6.173	171.028	199.455	-5.846	-0.06	39.6	0	0
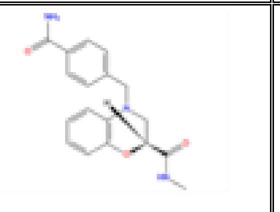	**MPRO**	-7.658907	325.366	8.069	605.009	173.041	165.934	266.034	0	1046.745	3	6.75	1.332	-3.203	-3.304	-4.273	150.318	117.483	-3.256	-0.357	84.66	0	0
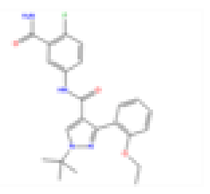	**MPRO**	-7.656709	424.474	11.708	749.166	297.504	140.838	274.251	36.573	1349.659	3	6.75	4.092	-6.417	-6.033	-6.264	457.442	336.947	-2.573	0.598	99.24	0	1

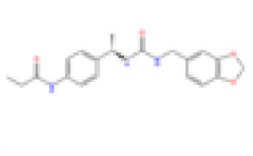	**MPRO**	-7.656456	369.419	9.11	690.427	354.514	92.783	243.13	0	1198.026	3	6	2.974	-4.927	-4.401	-4.713	960.754	660.349	-1.797	0.013	88.69	0	0
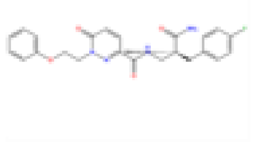	**MPRO**	-7.656212	438.457	7.479	770.462	139.213	161.181	423.258	46.81	1374.632	2	6.75	3.859	-5.26	-5.814	-5.8	165.446	237.205	-2.039	0.224	116.31	0	0
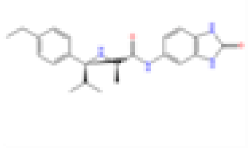	**MPRO**	-7.654702	380.489	8.888	685.561	357.053	136.01	192.497	0	1251.623	4	6	2.873	-3.851	-4.171	-6.1	126.769	58.708	-4.739	0.369	89.78	0	0
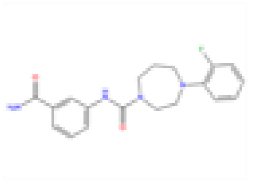	**MPRO**	-7.652268	356.399	6.643	634.667	162.608	146.976	302.414	22.669	1112.046	3	5.5	2.436	-4.786	-3.883	-4.546	279.61	244.613	-2.971	0.065	78.67	0	0

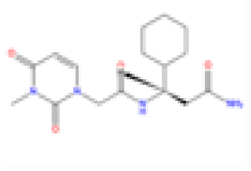	**MPRO**	-7.651972	336.39	9.364	627.727	352.55	205.489	69.688	0	1104.11	2	7.5	0.404	-2.511	-2.148	-1.979	46.379	46.189	-4.485	-0.718	116.19	0	0
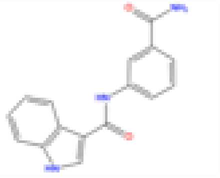	**MPRO**	-7.65138	279.298	10.007	531.598	0	179.332	352.266	0	892.665	3	4	2.021	-3.806	-4.038	-5.746	197.376	85.634	-3.296	0.023	87.98	0	0
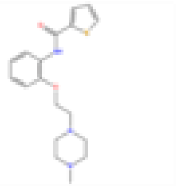	**ACE2**	-7.649969	345.459	6.911	664.26	282.187	57.266	278.801	46.006	1152.776	1	7.25	2.521	-2.483	-2.019	-7.543	176.46	165.9	-5.142	0.059	73.05	0	0
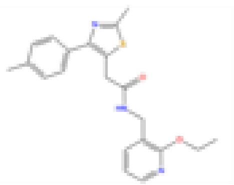	**MPRO**	-7.648012	381.492	4.467	716.141	374.917	45.049	260.817	35.358	1261.151	1	5	4.601	-6.037	-5.349	-4.827	2647.418	3182.29	-0.855	0.483	92.35	0	2

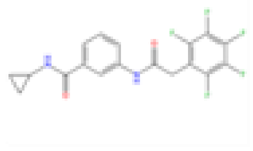	**MPRO**	-7.647161	384.305	8	658.376	193.635	83.48	190.444	190.817	1113.435	2	5	4.374	-6.364	-5.479	-5.853	1600.51	9129.024	-1.907	0.397	58.2	0	1
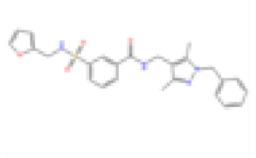	**MPRO**	-7.646959	478.565	9.25	803.954	232.14	129.083	441.023	1.708	1482.631	2	8.5	4.521	-6.235	-6.816	-7.16	591.294	286.452	-1.481	0.545	114.61	0	1
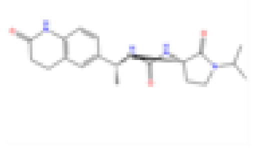	**MPRO**	-7.6444	358.439	12.698	647.645	402.822	161.028	83.794	0	1163.509	2.25	6.75	1.35	-3.748	-3.077	-1.997	150.131	131.904	-3.808	-0.361	90.54	0	0
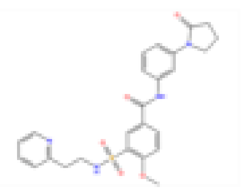	**MPRO**	-7.644347	494.564	9.01	764.625	293.89	180.772	288.301	1.661	1429.814	2	11.75	2.358	-4.629	-5.722	-6.099	191.265	84.524	-3.067	-0.141	126.08	0	0

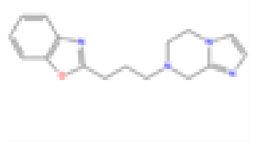	**ACE2**	-7.644226	282.344	3.216	565.84	213.409	57.898	294.533	0	959.938	0	5.5	2.296	-2.189	-2.354	-6.386	697.843	370.98	-3.228	-0.236	47.09	0	0
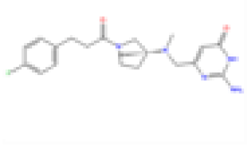	**ACE2**	-7.643808	373.429	8.618	667.723	237.015	204.618	179.279	46.811	1187.274	3	9	0.614	-1.991	-2.306	-4.779	15.07	20.984	-6.05	-0.503	95.32	0	1
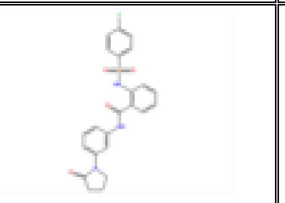	**MPRO**	-7.643129	453.487	8.079	764.445	148.25	149.1	419.599	47.496	1343.872	1	9	3.543	-6.159	-5.994	-7.248	381.931	318.205	-2.309	0.225	103.96	0	1
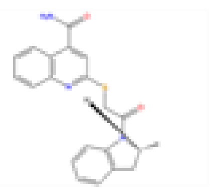	**MPRO**	-7.642768	377.46	8.669	654.212	151.495	138.045	332.444	32.228	1166.327	2	6.5	3.319	-5.302	-5.269	-6.045	486.206	340.711	-2.509	0.335	101.59	0	0

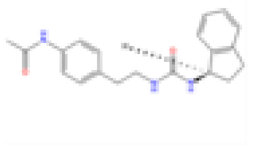	**MPRO**	-7.640749	337.421	9.05	699.034	267.344	128.045	303.645	0	1186.005	3	4.5	3.28	-5.013	-4.164	-5.244	312.938	287.302	-2.33	0.252	70.23	0	0
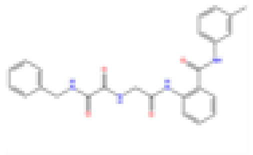	**MPRO**	-7.640353	444.489	4.559	827.037	182.662	149.247	495.128	0	1444.721	1.5	7.5	4.658	-7.168	-6.311	-7.982	380.708	174.189	-1.758	0.671	116.4	0	1
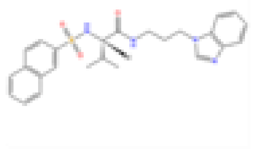	**MPRO**	-7.638746	464.581	7.858	778.65	218.336	130.147	428.737	1.429	1441.32	1.25	7.75	4.024	-5.318	-6.042	-5.56	381.858	278.371	-1.544	0.277	101.47	0	0
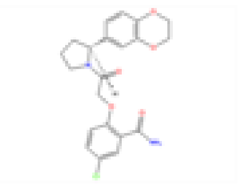	**MPRO**	-7.637594	416.86	5.322	647.227	274.53	110.899	190.263	71.534	1195.151	2	7.75	2.572	-4.159	-4.793	-3.63	516.907	1061.559	-2.509	-0.112	91.09	0	0

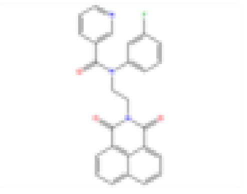	**MPRO**	-7.63636	439.445	5.491	705.235	38.46	118.946	500.971	46.858	1290.563	0	7.5	4.004	-5.045	-6.425	-6.949	737.792	643.116	-1.467	0.146	70.58	0	0
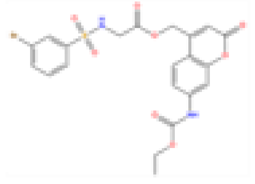	**MPRO**	-7.636072	539.354	11.601	684.276	226.477	238.429	181.75	37.62	1307.463	1.25	10.75	1.7	-3.671	-6.789	-4.906	54.309	34.118	-4.505	-0.271	149.39	1	0
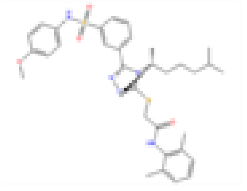	**MPRO**	-7.635464	635.838	3.262	909.605	439.204	112.635	331.755	26.01	1858.157	2	9.75	6.45	-7.058	-9.552	-6.292	846.804	573.822	-1.179	1.14	148.89	2	1
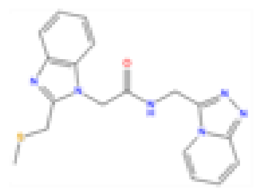	**MPRO**	-7.632684	366.44	13.855	613.567	194.938	102.13	270.949	45.551	1114.831	1	6.5	2.731	-3.776	-4.507	-3.918	788	940.809	-1.871	-0.186	102.93	0	0

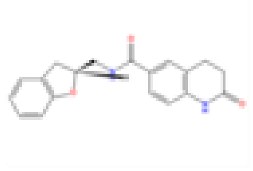	**MPRO**	-7.632513	322.363	6.037	619.585	204.565	122.77	292.249	0	1053.71	2	5.75	2.844	-4.968	-4.338	-6.028	678.691	325.392	-2.465	0.211	67.43	0	0
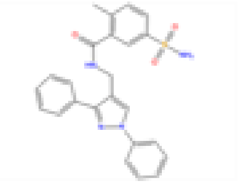	**MPRO**	-7.629789	446.523	9.8	763.058	89.696	186.087	485.463	1.813	1359.408	3	8	3.461	-6.293	-6.367	-7.443	170.309	74.704	-2.759	0.445	115.46	0	1
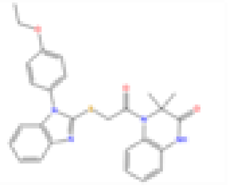	**MPRO**	-7.629347	486.587	4.686	719.797	258.651	57.297	369.96	33.89	1412.228	1	7.75	5.078	-5.808	-7.43	-5.793	2835.021	2339.698	-0.792	0.715	101.76	1	1
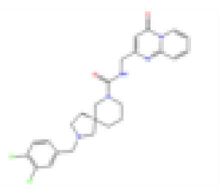	**MPRO**	-7.628686	500.427	8.774	795.002	277.991	94.03	293.068	129.913	1469.357	1	8	4.167	-5.822	-5.527	-5.74	231.049	814.054	-3.899	0.504	70.47	1	1

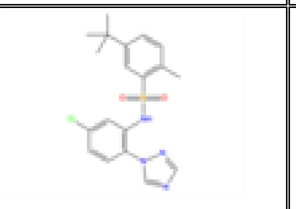	**MPRO**	-7.628456	404.913	3.533	670.678	250.817	105.721	242.455	71.685	1217.916	1	7	3.821	-5.564	-5.662	-5.563	984.799	1201.851	-2.23	0.379	85.26	0	0
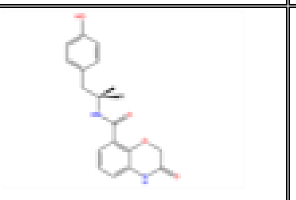	**MPRO**	-7.626542	326.351	9.225	583.801	175.772	154.867	253.163	0	1029.201	3	6.5	2.024	-3.786	-4.111	-5.257	336.74	152.55	-3.002	-0.063	87.66	0	0
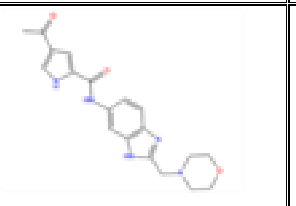	**ACE2**	-7.627306	367.407	6.2	668.655	302.175	169.035	197.445	0	1163.719	2	8.7	1.401	-3.336	-3.141	-6.433	61.638	26.928	-5.522	-0.115	103.11	0	0
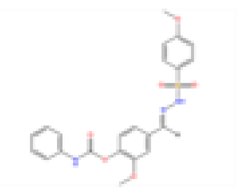	**MPRO**	-7.623766	455.484	10.128	780.429	203.448	150.466	424.753	1.762	1372.219	2	9	3.451	-5.663	-5.924	-7.357	370.705	173.05	-1.932	0.15	123.7	0	0

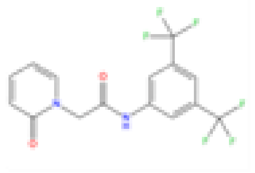	**MPRO**	-7.623712	364.247	9.286	583.221	42.073	79.763	227.557	233.828	986.082	1	5.5	3.775	-5.461	-5.141	-5.506	1735.831	10000	-1.9	0.106	51.1	0	0
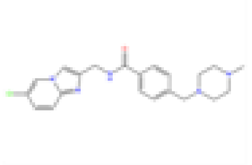	**ACE2**	-7.623186	397.906	3.743	737.453	304.705	69.546	291.397	71.805	1288.395	1	8	3.101	-3.746	-3.003	-7.991	134.956	171.908	-5.42	0.292	53.4	0	0
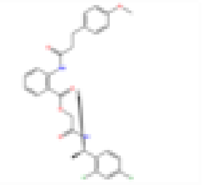	**MPRO**	-7.620529	496.51	5.821	874.099	301.694	115.427	376.901	80.077	1557.62	1	6.75	5.535	-7.546	-7.052	-6.106	513.824	1062.51	-1.359	0.738	93.73	1	1
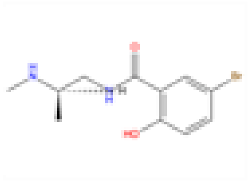	**ACE2**	-7.621056	287.156	3.793	491.449	204.739	94.888	114.553	77.269	815.016	2	3.75	1.91	-2.147	-2.784	-5.092	311.158	410.661	-4.447	-0.133	61.36	0	0

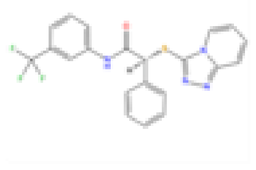	**MPRO**	-7.618894	428.431	12.831	686.58	2.311	82.251	454.107	147.911	1220.296	1	4.5	5.582	-6.956	-7.395	-6.863	1644.052	5469.964	-0.956	0.844	85.11	1	1
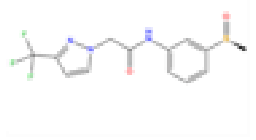	**MPRO**	-7.618535	331.312	8.148	569.568	128.101	97.689	201.918	141.861	967.555	1	8	2.136	-2.491	-3.444	-5.236	46.722	3520.506	-2.225	-0.458	83.2	0	0
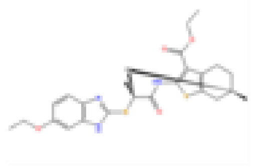	**MPRO**	-7.618485	487.631	9.997	762.087	469.506	88.389	137.735	66.456	1437.154	1	5.75	5.709	-6.988	-7.665	-5.293	1437.825	1693.792	-1.895	1.055	146.85	1	1
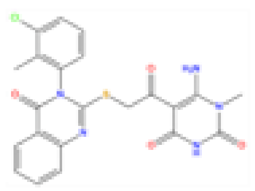	**MPRO**	-7.617443	483.928	8.285	714.002	164.456	195.089	272.889	81.569	1316.735	1	8	3.467	-5.819	-7.158	-5.848	139.917	165.185	-3.674	0.389	158.14	0	1

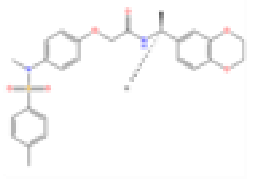	**MPRO**	-7.61314	496.577	11.744	827.081	399.189	110.793	315.432	1.667	1503.15	1	9.25	3.776	-5.597	-6.127	-5.428	544.278	440.862	-1.682	0.146	102.55	0	0
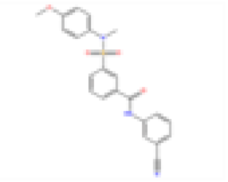	**MPRO**	-7.608325	421.47	7.657	621.463	125.563	171.859	322.322	1.719	1194.427	1	9.25	2.138	-4.105	-6.145	-5.075	232.36	104.391	-2.879	-0.225	107.88	0	0
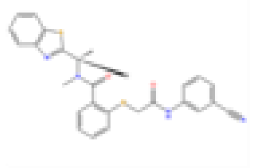	**MPRO**	-7.604902	486.605	12.202	706.68	182.236	111.177	343.467	69.799	1387.408	1	9	4.128	-5.77	-7.739	-5.576	874.187	1031.79	-1.59	0.266	139.63	0	1
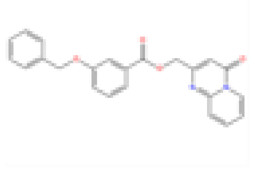	**MPRO**	-7.60401	386.406	2.258	730.355	71.121	123.982	535.252	0	1254.76	0	6.75	3.963	-5.546	-5.467	-7.801	660.97	316.219	-1.343	0.2	70.42	0	0

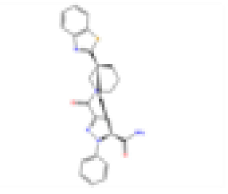	**MPRO**	-7.602391	433.527	6.336	689.025	205.531	161.271	286.95	35.273	1282.043	2	8	2.621	-3.888	-5.261	-4.304	104.326	204.644	-3.289	0.136	120.13	0	0
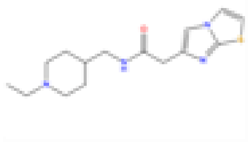	**ACE2**	-7.597419	306.425	4.515	558.609	327.043	53.294	143.885	34.386	1009.39	1	6	1.913	-1.537	-1.898	-3.655	465.553	638.117	-3.577	-0.31	78.4	0	0
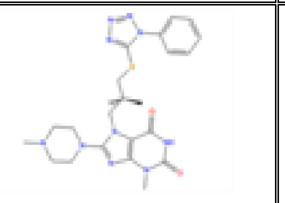	**ACE2**	-7.596339	496.589	7.745	729.83	361.335	168.267	174.7	25.528	1409.413	1	10.5	2.325	-3.664	-5.313	-5.794	62.68	37.836	-5.588	0.128	148.06	1	0
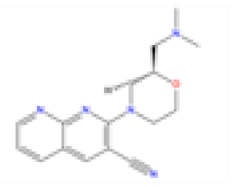	**ACE2**	-7.596166	297.359	4.599	575.188	289.114	91.56	194.513	0	996.944	0	7.7	1.116	-2.377	-2.468	-5.79	334.607	167.614	-4.296	-0.675	65.28	0	0

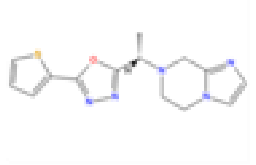	**ACE2**	-7.595696	301.365	3.195	540.394	182.326	74.845	244.748	38.475	941.944	0	6	1.907	-1.939	-2.586	-5.692	482.003	404.027	-3.907	-0.337	88.22	0	0
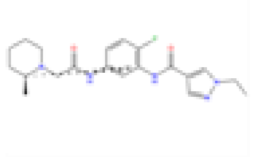	**ACE2**	-7.589474	387.456	6.406	740.969	410.574	107.058	185.033	38.303	1291.171	2	8.5	2.794	-4.597	-3.355	-6.799	238.543	188.485	-4.327	0.172	79.26	0	0
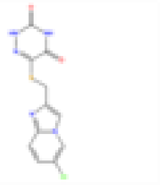	**ACE2**	-7.577509	309.729	5.758	514.303	34.1	196.191	180.307	103.705	856.468	2	4.5	1.889	-4.004	-4.554	-4.851	136.589	212.781	-4.212	-0.06	121.73	0	0
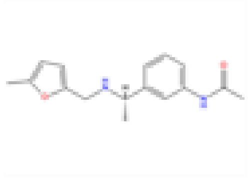	**ACE2**	-7.57716	272.346	6.188	591.843	312.827	62.633	216.383	0	992.623	2	4.5	2.75	-3.315	-2.542	-6.314	629.294	331.753	-3.494	0.18	54.27	0	1

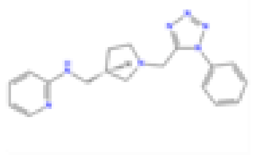	ACE2	-7.576307	335.411	7.971	658.187	173.14	111.81	373.237	0	1136.329	1	6.5	2.817	-3.839	-3.462	-7.272	215.035	103.933	-3.848	0.209	71.76	0	0
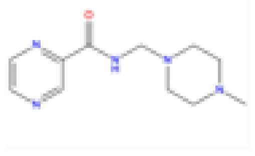	ACE2	-7.566398	235.288	5.279	500.875	261.033	99.389	140.454	0	834.655	1	9	-0.723	0.536	1.008	-5.985	70.339	34.361	-6.694	-0.899	61.36	0	0
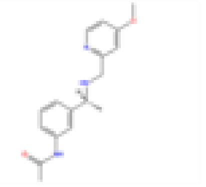	ACE2	-7.566234	299.372	4.94	615.219	307.07	85.933	222.216	0	1052.253	2	5.75	2.485	-3.187	-2.689	-6.34	378.353	191.42	-3.807	0.084	63.25	0	1
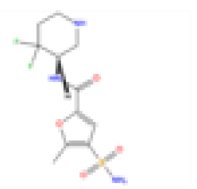	ACE2	-7.550276	323.314	3.102	542.239	232.313	211.152	41.781	56.993	914.192	4	9	-0.573	-1.87	-1.594	-5.045	24.572	20.451	-6.942	-0.605	122.81	0	0

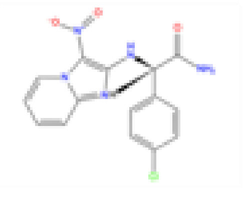	**ACE2**	-7.548985	345.744	8.065	557.782	0	185.378	300.892	71.512	972.834	2.25	4.75	1.937	-3.008	-4.512	-3.999	90.812	182.984	-3.396	-0.224	116.08	0	0
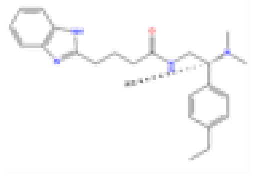	**ACE2**	-7.538094	378.516	1.02	751.227	393.323	76.706	281.198	0	1342.219	2	6	3.677	-4.228	-3.676	-5.731	309.894	237.994	-3.141	0.326	61.02	0	0
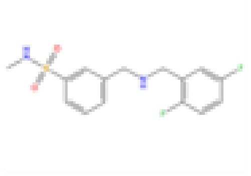	**ACE2**	-7.527599	326.36	6.308	596.035	147.927	108.43	258.053	81.624	1010.069	2	6	2.331	-3.15	-3.118	-6.453	231.502	315.156	-4.095	-0.062	66.58	0	0
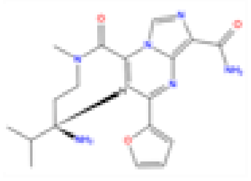	**ACE2**	-7.521512	384.437	6.146	692.471	264.157	215.65	212.664	0	1229.706	4	9.5	0.938	-3.143	-3.01	-6.482	22.274	8.962	-6.135	-0.2	133.27	0	1

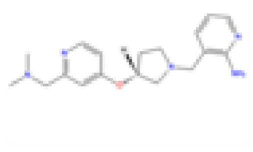	**ACE2**	-7.520437	327.428	1.263	653.815	332.174	89.901	231.741	0	1136.315	2	7.25	1.869	-1.997	-1.684	-7.237	86.53	42.985	-5.813	-0.009	67.51	0	1
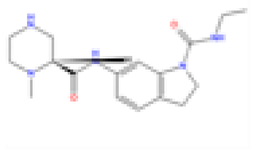	**ACE2**	-7.505628	331.417	5.268	653.198	449.322	101.063	102.813	0	1126.136	2	7	1.047	-2.075	-0.958	-5.314	47.588	33.029	-6.857	-0.147	76.71	0	0
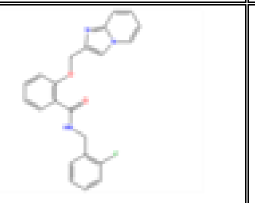	**ACE2**	-7.504407	375.401	2.446	694.757	81.23	50.84	536.06	26.627	1204.72	1	4.75	5.222	-6.317	-6.205	-7.517	3264.289	2486.318	0.008	0.702	56.15	1	1
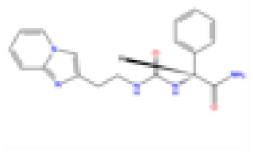	**ACE2**	-7.495692	337.38	5.066	553.004	98.244	154.475	300.284	0	1028.172	3.25	5.25	1.262	-1.326	-3.439	-1.981	109.133	153.967	-2.733	-0.558	102.04	0	0

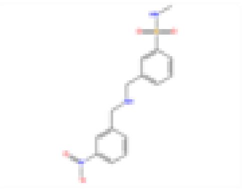	**ACE2**	-7.486144	335.377	7.168	622.634	149.704	206.544	264.737	1.649	1055.59	2	7	1.238	-2.785	-2.932	-6.672	27.173	11.344	-5.784	-0.167	109.72	0	0
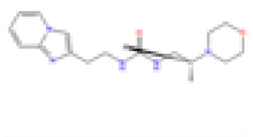	**ACE2**	-7.478851	331.417	5.339	646.24	345.461	79.394	221.385	0	1124.807	2	7.2	1.783	-2.665	-2.064	-5.024	312.346	223.361	-3.689	-0.305	71.42	0	0
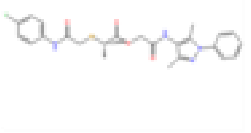	**ACE2**	-7.465323	484.544	8.291	888.116	302.042	139.484	371.717	74.873	1543.744	2	8.5	4.983	-8.17	-6.636	-7.716	471.164	563.949	-2.013	0.704	127.62	0	1
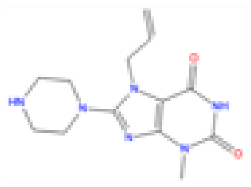	**ACE2**	-7.460227	290.324	7.182	514.637	325.38	142.292	46.966	0	910.482	2	7	0.623	-1.87	-2.008	-4.379	110.52	50.618	-5.847	-0.231	87.95	0	0

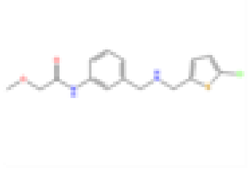	**ACE2**	-7.445454	324.824	7.319	607.458	217.233	56.13	230.713	103.381	1027.435	2	5.7	2.96	-3.359	-3.135	-6.423	725.297	1424.905	-3.132	-0.015	78.6	0	1
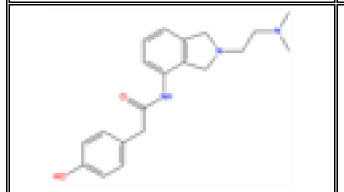	**ACE2**	-7.443014	339.436	4.158	690.631	306.193	108.371	276.066	0	1193.033	2	7.25	2.126	-2.611	-1.921	-7.736	57.812	27.797	-5.997	0.119	55.81	0	1
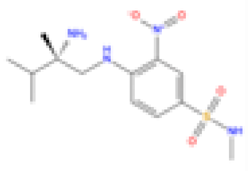	**ACE2**	-7.442683	330.401	6.797	588.603	295.48	215.045	76.489	1.59	1032.119	4	7.5	0.387	-1.87	-2.212	-5.226	22.57	9.275	-6.508	-0.366	135.74	0	1
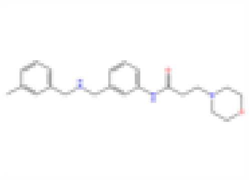	**ACE2**	-7.438191	367.49	5.19	716.644	389.14	66.054	261.449	0	1268.261	2	7.7	2.689	-2.74	-2.344	-7.618	145.65	75.465	-5.173	0.19	53.6	0	1

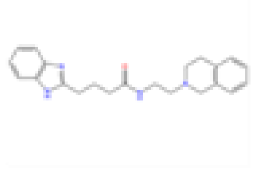	**ACE2**	-7.425942	362.474	6.574	732.411	285.689	88.299	358.422	0	1268.518	2	6	3.219	-3.981	-3.405	-6.254	210.07	181.022	-3.275	0.209	61.02	0	0
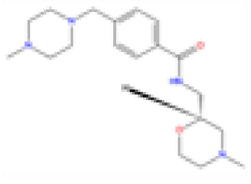	**ACE2**	-7.415595	346.472	4.944	687.662	511.727	52.182	123.753	0	1208.77	1	10.2	0.775	-0.516	0.368	-7.569	49.177	25.819	-5.69	-0.355	48.05	0	0
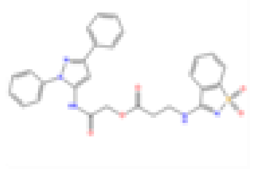	**ACE2**	-7.414	529.569	11.503	917.639	108.719	217.629	589.783	1.509	1613.357	1	10	4.324	-7.925	-7.432	-8.795	85.532	35.348	-2.781	0.617	140.13	1	1
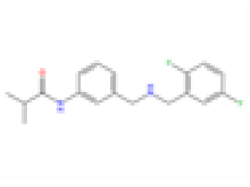	**ACE2**	-7.412559	318.366	4.696	650.527	244.585	41.022	282.084	82.836	1092.645	2	4	4.085	-4.681	-3.674	-7.023	1008.755	1570.704	-2.768	0.473	41.13	0	0

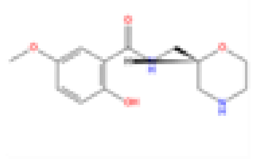	**ACE2**	-7.411243	266.296	5.651	523.862	297.944	113.466	112.451	0	882.994	2	6.2	0.998	-1.658	-1.66	-5.227	207.395	99.947	-4.797	-0.333	79.82	0	0
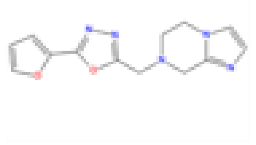	**ACE2**	-7.402563	271.278	4.912	516.59	150.262	93.486	272.842	0	867.818	0	6.5	0.937	-1.058	-1.715	-5.958	320.827	160.165	-4.152	-0.686	73.12	0	0
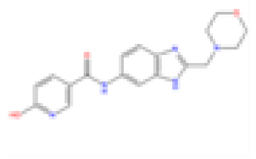	**ACE2**	-7.398419	353.38	3.353	635.808	218.845	167.613	249.35	0	1101.483	3	8.7	1.001	-2.841	-2.922	-6.501	63.581	27.846	-5.313	-0.25	103.37	0	0
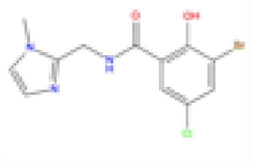	**ACE2**	-7.396006	344.595	6.192	536.412	117.44	93.68	180.991	144.301	893.592	1	3.75	3.423	-4.694	-5.372	-5.042	1280.949	3990.826	-2.224	0.158	67.15	0	0

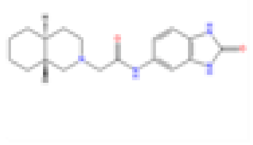	**ACE2**	-7.392527	328.413	6.963	624.473	327.866	170.774	125.833	0	1083.956	3	6.5	1.54	-3.537	-2.763	-5.816	59.341	25.845	-5.998	0.126	80.99	0	0
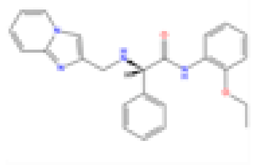	**ACE2**	-7.382268	400.479	3.995	771.996	169.159	62.406	540.431	0	1345.819	2	6.25	4.613	-5.393	-5.019	-8.66	632.421	333.535	-2.06	0.64	68.18	0	0
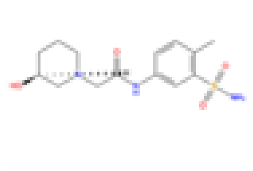	**ACE2**	-7.377284	327.398	12.976	601.662	278.144	219.822	102.023	1.674	1026.614	4	10.7	-0.763	-1.776	-1.19	-5.69	20.334	8.295	-6.698	-0.715	121.11	0	1
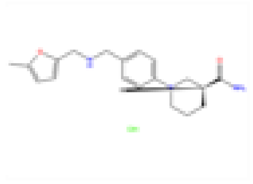	**ACE2**	-7.368915																			71.5		

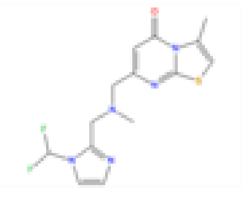	**ACE2**	-7.368598	339.362	3.833	584.672	209.287	64.182	172.441	138.762	1011.323	0	7.5	2.001	-2.104	-2.592	-5.777	608.36	1841.082	-3.773	-0.622	84.19	0	0
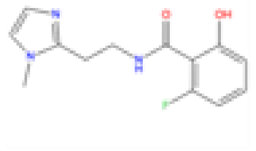	**ACE2**	-7.366927	263.271	8.468	530.298	142.865	102.804	248.173	36.456	877.149	1	3.75	3.072	-4.457	-3.727	-5.375	1049.567	825.596	-2.06	0.071	67.15	0	0
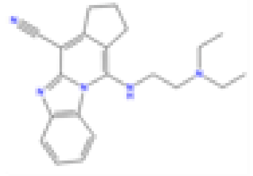	**ACE2**	-7.363593	347.462	12.33	652.286	356.758	93.017	202.511	0	1173.025	1	5.5	3.41	-4.78	-4.8	-6.061	324.129	161.947	-3.911	0.436	56.88	0	0
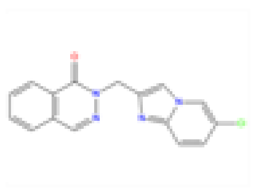	**ACE2**	-7.361447	310.742	4.704	549.681	41.797	62.333	373.746	71.805	940.344	0	4	3.762	-4.525	-4.956	-5.831	2539.793	3351.476	-1.16	0.193	52.71	0	0

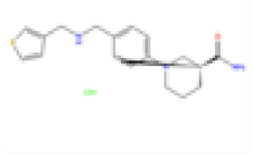	**ACE2**	-7.346939																			86.6		
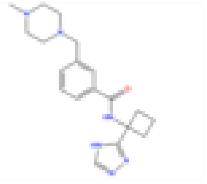	**ACE2**	-7.344405	354.454	11.953	684.497	398.262	106.841	179.394	0	1203.2	2	8.5	1.601	-2.6	-1.482	-7.088	59.775	28.819	-6.598	0.059	77.15	0	0
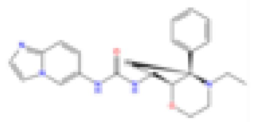	**ACE2**	-7.339682	379.461	8.183	687.841	228.811	88.837	370.193	0	1223.4	2	7.2	2.493	-4.001	-3.349	-5.817	268.558	178.74	-3.531	0.025	71.42	0	0
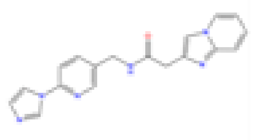	**ACE2**	-7.33799	332.364	7.7	631.993	91.639	100.798	439.556	0	1089.678	1	6.5	2.587	-4.015	-3.948	-5.323	723.71	546.542	-1.445	-0.188	77.63	0	0

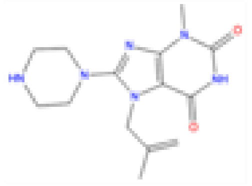	**ACE2**	-7.33643	304.351	8.103	514.289	340.305	145.182	28.802	0	934.615	2	7	0.74	-1.784	-2.267	-4.031	103.762	47.28	-5.964	-0.161	87.95	0	0
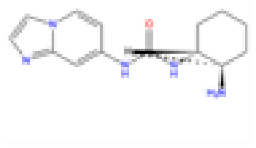	**ACE2**	-7.3361	273.337	9.299	558.621	207.153	130.917	220.552	0	930.43	4	4.5	0.888	-2.04	-1.595	-4.766	95.988	66.207	-4.93	-0.374	84.97	0	0
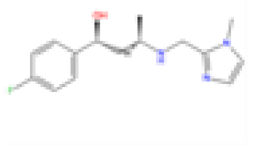	**ACE2**	-7.334131	277.341	4.879	555.38	190.578	79.746	238.24	46.815	957.033	2	4.7	2.808	-2.976	-2.57	-5.892	433.076	399.81	-3.541	-0.009	50.08	0	0
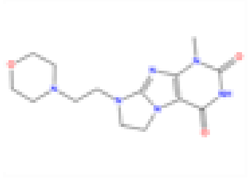	**ACE2**	-7.330341	320.35	5.6	549.917	398.882	133.093	17.942	0	971.006	1	9.2	0.165	-1.384	-1.75	-4.571	135.106	62.892	-5.684	-0.611	88.39	0	0

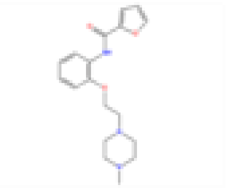	**ACE2**	-7.32206	329.398	6.744	639.348	282.8	61.805	294.744	0	1112.905	1	7.75	1.883	-1.669	-1.508	-7.427	159.811	83.426	-5.169	-0.138	57.95	0	0
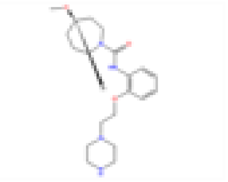	**ACE2**	-7.309969	376.498	7.298	703.092	483.784	65.633	153.674	0	1268.179	2	7.95	1.853	-2.155	-1.036	-5.484	105.262	76.218	-5.737	-0.08	66.07	0	0
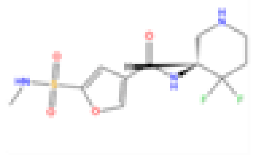	**ACE2**	-7.306415	323.314	8.631	538.488	232.383	177.146	72.453	56.506	917.411	3	9	-0.171	-1.774	-1.617	-5.064	51.633	45.352	-6.208	-0.595	108.82	0	0
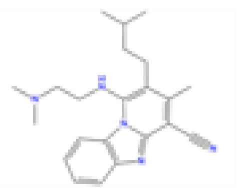	**ACE2**	-7.305675	363.505	11.975	681.21	392.713	94.279	194.218	0	1247.545	1	5.5	3.862	-5.13	-5.138	-6.053	315.321	157.196	-3.867	0.594	56.88	0	0

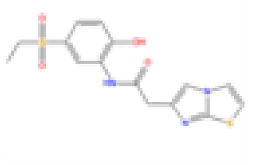	**ACE2**	-7.304225	365.421	14.8	617.576	167.718	164.746	228.898	56.214	1078.961	2	8.75	1.704	-3.807	-4.033	-5.475	271.404	245.524	-3.173	-0.346	137.91	0	0
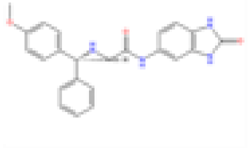	**ACE2**	-7.302589	402.452	9.819	728.311	132.67	177.373	418.268	0	1273.941	4	6.75	2.677	-4.372	-4.698	-7.807	51.377	22.117	-4.706	0.278	99.01	0	0
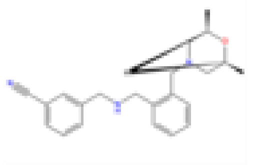	**ACE2**	-7.301904	349.475	3.947	710.759	317.825	81.729	311.205	0	1243.946	1	6.7	3.011	-4.14	-3.375	-7.915	103.435	52.129	-5.383	0.364	48.29	0	0
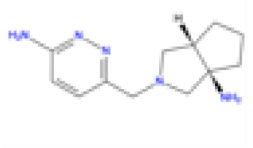	**ACE2**	-7.279934	233.316	4.78	483.209	242.124	154.441	86.644	0	814.597	4	5.5	-0.28	-0.073	-0.099	-5.453	21.141	9.371	-7.802	-0.41	81.06	0	1

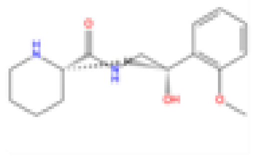	**ACE2**	-7.278091	278.35	4.226	575.036	336.357	89.639	149.04	0	980.428	3	6.45	0.914	-1.62	-1.065	-4.345	242.418	175.388	-4.133	-0.549	70.59	0	0
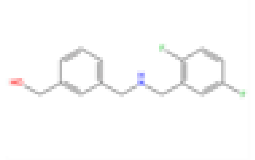	**ACE2**	-7.273845	263.286	1.272	535.008	120.014	60.175	275.533	79.285	889.512	2	3.2	3.011	-2.986	-2.861	-6.225	663.986	955.716	-3.144	0.087	32.26	0	0
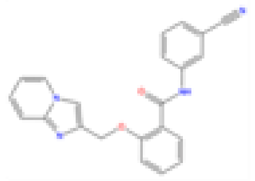	**ACE2**	-7.272896	368.394	7.612	697.512	35.663	123.562	538.288	0	1201.685	1	6.25	3.964	-6.68	-6.466	-7.599	667.058	319.368	-1.324	0.384	79.94	0	1
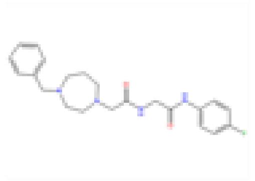	**ACE2**	-7.271333	398.479	9.225	746.133	267.382	85.355	346.577	46.819	1320.666	1.25	8.25	2.381	-2.658	-1.677	-6.819	63.498	86.375	-5.325	-0.069	64.68	0	0

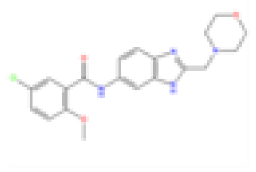	**ACE2**	-7.268946	400.864	4.02	699.58	312.751	82.823	232.459	71.547	1221.074	2	8.45	2.749	-4.381	-3.937	-6.769	404.936	507.92	-3.81	0.061	79.48	0	0
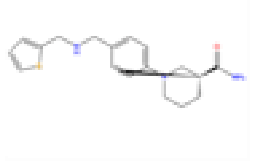	**ACE2**	-7.26034	329.459	5.427	623.37	214.272	120.069	240.792	48.237	1091.302	3	5	2.179	-2.503	-2.783	-4.907	88.877	157.158	-4.467	-0.037	86.6	0	0
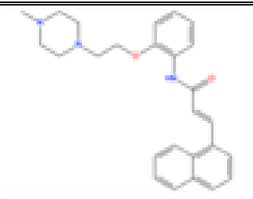	**ACE2**	-7.25658	415.534	5.526	815.057	308.322	61.467	445.268	0	1425.099	1	7.25	4.198	-4.613	-3.72	-9.13	160.994	84.094	-4.441	0.677	44.81	0	0
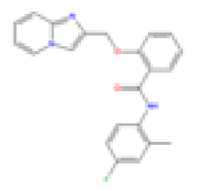	**ACE2**	-7.256458	375.401	6.613	691.971	107.874	47.582	489.647	46.869	1200.064	1	4.75	5.239	-6.518	-6.205	-7.277	3504.969	3466.053	-0.191	0.732	56.15	1	1

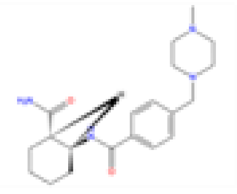	**ACE2**	-7.2544	358.483	2.144	683.052	441.196	122.528	119.328	0	1219.428	2	8	1.102	-1.798	-1.032	-5.284	27.655	19.901	-7.002	-0.167	78.67	0	0
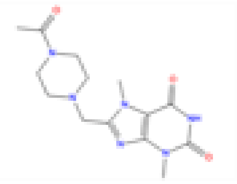	**ACE2**	-7.252306	320.35	1.471	565.766	378.849	172.245	14.673	0	1002.49	1	10	-0.878	-0.371	-0.749	-3.28	30.366	24.963	-6.513	-0.971	96.23	0	0
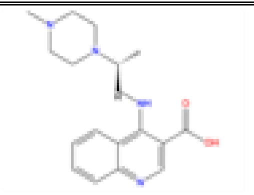	**ACE2**	-7.251865	328.413	3.645	612.465	297.962	117.734	196.769	0	1087.185	1	7	0.189	-2.311	-1.721	-4.787	11.935	6.424	-6.641	-0.161	68.7	0	1
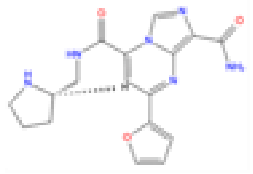	**ACE2**	-7.250268	354.368	7.873	633.778	212.976	192.196	228.606	0	1094.188	4	9.5	0.348	-2.773	-2.515	-6.419	37.171	15.588	-5.935	-0.355	128.07	0	0

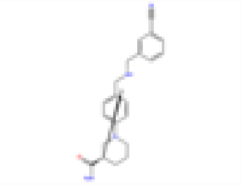	**ACE2**	-7.247289	348.447	3.687	677.845	212.632	185.469	279.744	0	1186.358	3	6.5	1.598	-3.652	-3.698	-5.461	21.359	18.27	-5.439	-0.087	82.15	0	1
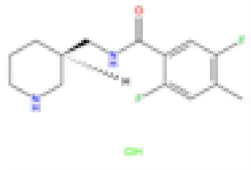	**ACE2**	-7.244727																			41.13		
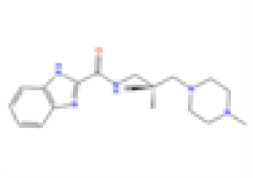	**ACE2**	-7.198821	315.417	1.511	640.184	351.025	94.841	194.317	0	1105.1	2	8	1.31	-1.87	-1.073	-7.026	77.682	38.254	-6.228	-0.129	64.26	0	0
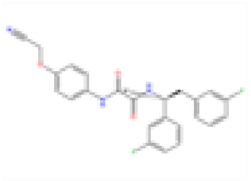	**TMPRSS2**	-7.198013	435.429	5.901	758.866	79.377	139.358	447.359	92.773	1337.641	0.5	5.75	5.223	-7.861	-7.858	-7.328	472.468	708.916	-1.648	0.721	91.22	1	1

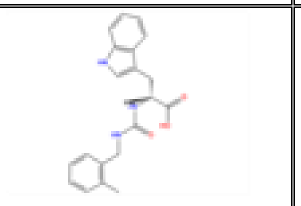	**TMPRSS2**	-7.196331	351.404	1.402	648.738	118.106	165.673	364.959	0	1136.756	3.25	3.25	3.725	-4.755	-5.026	-3.133	46.367	34.076	-2.711	0.102	94.22	0	0
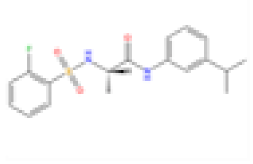	**TMPRSS2**	-7.193332	364.434	5.51	579.16	244.487	102.546	202.749	29.378	1080.461	1.25	6.25	3.1	-3.775	-4.846	-4.453	1055.491	759.684	-2.119	0.12	83.65	0	0
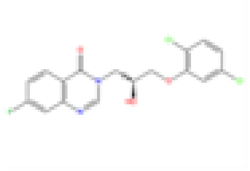	**TMPRSS2**	-7.190593	383.206	2.998	627.08	61.708	101.542	280.118	183.713	1079.409	1	6.45	3.621	-5.145	-5.385	-5.918	1078.896	5449.733	-1.828	0.033	64.35	0	0
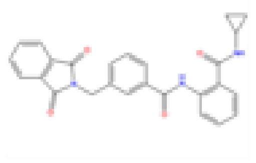	**TMPRSS2**	-7.180751	439.47	9.343	708.867	139.296	133.923	435.649	0	1320.028	1	7	4.219	-5.594	-6.408	-6.507	531.998	250.087	-1.877	0.566	95.58	0	0

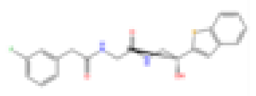	**TMPRSS2**	-7.173833	386.44	5.309	676.549	146.915	105.569	350.51	73.554	1193.44	2.25	5.95	2.803	-4.01	-4.24	-3.622	468.999	1234.943	-1.462	-0.336	106.67	0	0
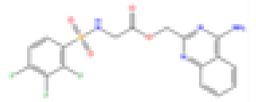	**TMPRSS2**	-7.160395	426.369	11.352	696.444	66.994	228.497	285.014	115.939	1184.441	2.25	8.25	2.313	-5.333	-5.509	-6.494	67.463	115.831	-4.055	-0.093	132.65	0	0
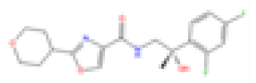	**TMPRSS2**	-7.159434	366.364	9.028	613.691	311.3	70.187	161.739	70.465	1088.784	2	6.95	3.097	-4.499	-4.571	-4.989	2139.515	2737.735	-1.763	0.032	84.59	0	0
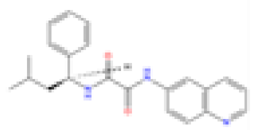	**TMPRSS2**	-7.149125	361.443	3.133	698.042	181.974	95.675	420.392	0	1228.936	0.5	4.5	5.004	-6.367	-5.734	-6.856	1226.353	616.784	-1.226	0.832	71.09	1	1

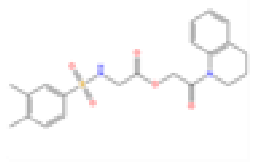	**TMPRSS2**	-7.148417	416.491	7.66	750.631	325.174	182.811	240.844	1.801	1317.019	0.25	8.75	2.833	-5.373	-4.83	-6.336	182.936	80.696	-3.464	0.053	101.16	0	0
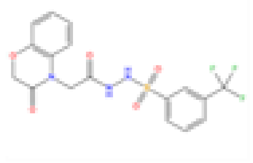	**TMPRSS2**	-7.143432	429.37	7.185	642.193	75.412	166.892	282.141	117.747	1132.701	1.25	10	1.837	-4.088	-4.829	-5.818	258.978	507.196	-3.121	-0.457	113.19	0	0
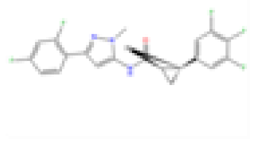	**TMPRSS2**	-7.139957	407.342	9.809	647.691	172.131	45.146	223.073	207.341	1158.454	1	3.5	5.828	-7.375	-6.929	-5.45	3696.428	10000	-1.374	1.009	46.92	1	1
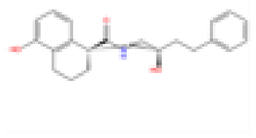	**TMPRSS2**	-7.139395	339.433	4.189	659.73	246.553	112.288	300.889	0	1163.333	3	4.95	3.1	-3.778	-4.046	-4.653	453.669	416.728	-1.761	0.019	69.56	0	0

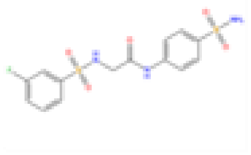	**TMPRSS2**	-7.139361	387.4	9.787	642.752	31.129	278.874	282.589	50.16	1086.52	3.25	10.75	0.103	-3.556	-3.591	-6.193	22.456	15.386	-4.992	-0.693	152.19	0	1
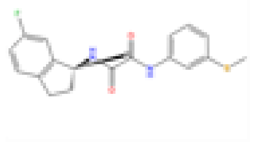	**TMPRSS2**	-7.129755	344.403	3.595	640.97	202.911	87.038	262.124	88.897	1092.952	0.5	4	4.567	-6.246	-5.335	-6.009	1480.862	2320.783	-1.816	0.65	83.5	0	1
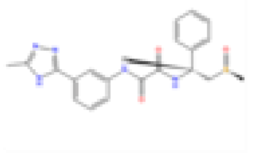	**TMPRSS2**	-7.126673	411.478	7.882	746.337	202.535	205.884	319.503	18.415	1295.551	1.25	9.25	2.502	-4.204	-4.899	-6.776	4.401	57.726	-3.612	0.02	136.05	0	1
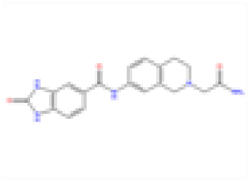	**ACE2**	-7.125758	365.391	2.756	654.339	143.284	283.504	227.551	0	1126.27	5	9	-0.724	-1.796	-2.235	-5.204	2.459	1.807	-7.621	-0.601	124.08	0	1

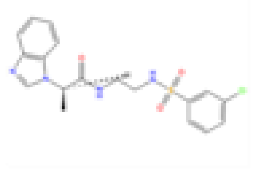	**TMPRSS2**	-7.125183	406.886	5.164	640.292	132.761	143.949	311.817	51.765	1173.513	2	8.5	1.985	-3.371	-4.342	-4.228	291.915	379.222	-2.402	-0.423	101.47	0	0
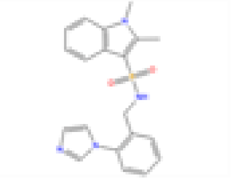	**TMPRSS2**	-7.124544	380.464	11.12	636.819	145.355	94.849	395.518	1.098	1156.995	1	6	3.85	-5.037	-5.749	-6.056	1248.669	637.694	-1.49	0.426	77.3	0	0
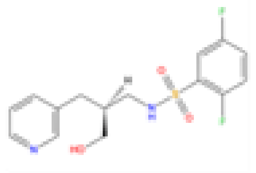	**TMPRSS2**	-7.123749	342.36	5.687	556.93	90.353	134.098	259.071	73.408	983.273	2	7.7	1.883	-2.908	-3.86	-5.055	529.968	628.678	-2.31	-0.448	87.67	0	0
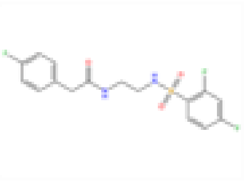	**TMPRSS2**	-7.121373	372.361	7.1	630.702	113.691	115.692	282.292	119.028	1082.843	2	7	2.405	-3.948	-4.052	-4.601	537.341	1725.801	-1.985	-0.419	83.65	0	0

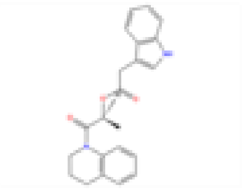	**TMPRSS2**	-7.120618	362.427	6.637	676.998	237.861	79.23	359.907	0	1192.818	1	5	4.543	-6.114	-5.527	-6.429	1756.139	909.263	-1.328	0.709	62.4	0	1
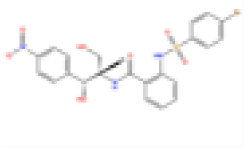	**TMPRSS2**	-7.119263	550.38	5.289	786.923	59.506	268.469	380.872	78.076	1419.012	3	10.4	2.489	-5.317	-7.201	-6.972	28.185	27.97	-4.07	-0.073	167.25	1	0
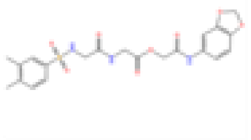	**TMPRSS2**	-7.118744	477.488	5.774	778.932	353.272	216.943	207.058	1.659	1393.637	1.5	11.5	1.261	-4.185	-4.487	-4.792	65.158	35.993	-3.924	-0.609	157.51	1	0
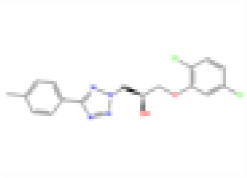	**TMPRSS2**	-7.115764	379.245	2.889	673.94	163.33	103.54	270.211	136.859	1150.596	1	5.95	4.065	-5.974	-5.565	-6.252	1032.835	2878.931	-1.9	0.31	73.06	0	1

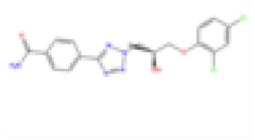	TMPRSS2	-7.113681	408.243	3.775	691.755	82.947	207.404	264.373	137.031	1185.612	3	8.45	2.351	-5.269	-5.104	-6.302	106.927	248.638	-3.739	-0.134	116.15	0	0
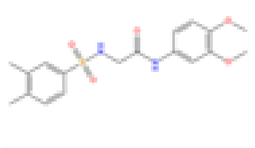	TMPRSS2	-7.113242	378.442	8.564	692.511	366.63	136.154	188.137	1.59	1196.49	1.25	7.75	2.791	-4.898	-4.539	-5.875	506.696	242.062	-2.694	0.033	102.11	0	0
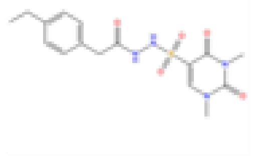	TMPRSS2	-7.112836	380.418	8.015	684.629	334.77	203.513	145.722	0.623	1179.999	1	9.5	1.447	-4.228	-3.871	-5.644	116.409	48.776	-4.181	-0.335	127.65	0	0
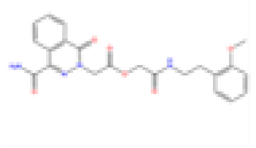	TMPRSS2	-7.108118	438.439	4.607	781.93	242.632	235.806	303.492	0	1371.885	3	9.75	1.653	-5.077	-4.507	-5.43	47.596	22.583	-3.932	-0.329	142.61	0	0

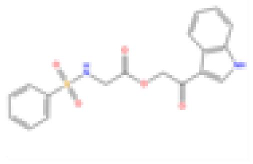	**TMPRSS2**	-7.106572	372.395	11.376	645.657	60.852	205.6	377.792	1.413	1123.387	0.25	6.75	2.519	-4.207	-4.92	-6.417	111.224	46.897	-3.306	-0.043	113.71	0	0
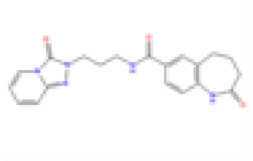	**TMPRSS2**	-7.104517	379.418	7.175	687.2	233.08	178.977	275.143	0	1202.409	2	8	2.458	-5.079	-4.444	-6.149	198.911	86.354	-3.369	0.101	98.02	0	0
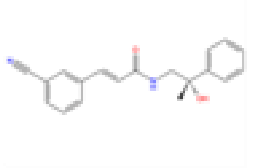	**TMPRSS2**	-7.100698	306.363	9.28	626.595	124.041	140.726	361.828	0	1067.935	2	4.75	3.268	-5.523	-5.221	-6.39	458.559	212.99	-2.07	0.203	73.12	0	0
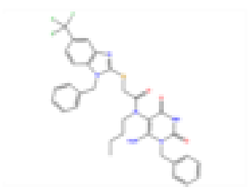	**TMPRSS2**	-7.099033	636.69	13.957	828.232	224.023	100.19	356.765	147.253	1682.305	3	8.5	6.306	-6.722	-10.219	-6.05	1111.218	3552.286	-0.957	0.935	144.31	2	1

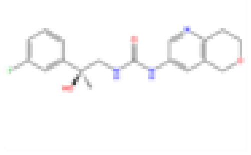	**TMPRSS2**	-7.095425	345.373	7.548	615.637	262.199	93.293	213.342	46.803	1077.988	3	5.45	2.611	-4.165	-4.056	-3.992	935.62	1177.411	-2.007	-0.134	83.48	0	0
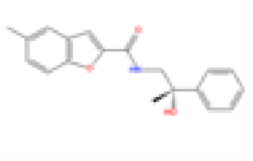	**TMPRSS2**	-7.090923	309.364	4.762	601.423	190.574	70.907	339.942	0	1040.559	2	3.75	3.956	-4.915	-4.811	-5.973	2106.149	1106.632	-1.149	0.436	62.47	0	0
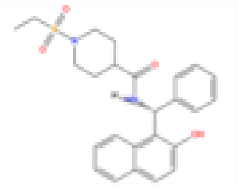	**TMPRSS2**	-7.089504	452.567	8.415	749.07	253.442	105.812	388.73	1.086	1387.553	2	7.75	3.724	-5.474	-5.625	-5.196	785.527	492.245	-1.428	0.219	95.09	0	0
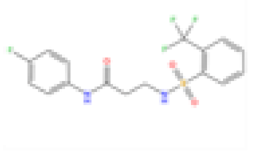	**TMPRSS2**	-7.088645	390.352	7.337	591.206	52.022	120.712	291.479	126.993	1056.239	1	6	3.405	-4.469	-5.47	-5.375	709.879	1694.98	-2.141	0.08	83.65	0	0

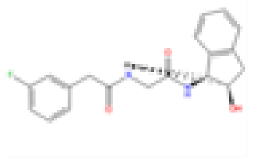	**TMPRSS2**	-7.083628	342.369	4.5	629.784	167.638	94.787	320.465	46.893	1097.01	2.25	5.95	2.155	-3.41	-3.183	-3.276	586.718	1137.925	-1.561	-0.488	78.43	0	0
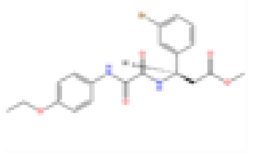	**TMPRSS2**	-7.082909	449.3	1.091	760.509	278.98	129.923	274.286	77.32	1310.404	0.25	6	4.739	-6.816	-6.663	-6.699	580.55	728.866	-2.18	0.602	93.73	0	1
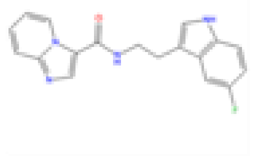	**TMPRSS2**	-7.082199	322.341	5.213	549.522	86.169	91.738	336.722	34.893	984.024	2	4	3.516	-4.263	-5.16	-5.231	1336.452	1051.076	-1.64	0.312	62.71	0	0
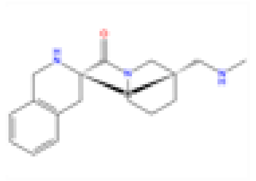	**ACE2**	-7.078639	287.404	2.728	580.966	355.941	58.263	166.762	0	1014.303	2	6	1.151	-1	-0.409	-4.989	110.004	90.699	-5.843	-0.228	44.37	0	0

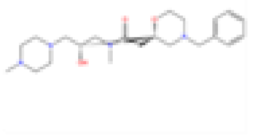	**ACE2**	-7.062327	404.551	3.138	778.981	522.587	67.043	189.351	0	1398.309	1	12.4	0.336	0.239	0.766	-6.858	22.943	18.181	-5.349	-0.838	59.49	0	2
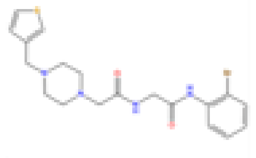	**ACE2**	-7.056257	451.38	4.416	737.258	247.003	80.781	283.956	125.517	1282.761	1.25	8.25	2.399	-2.479	-3.099	-6.614	57.219	259.641	-5.461	-0.172	92.92	0	1
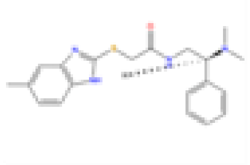	**ACE2**	-7.040755	368.496	4.712	603.772	301.717	63.367	203.659	35.029	1139.715	2	6	2.613	-2.249	-3.479	-3.895	428.802	507.196	-3.36	-0.057	86.32	0	0
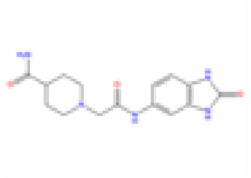	**ACE2**	-7.011648	317.347	8.631	589.06	188.963	274.875	125.222	0	1003.725	5	9	-1.432	-0.967	-1.05	-4.345	3.082	2.215	-7.823	-0.809	124.08	0	1

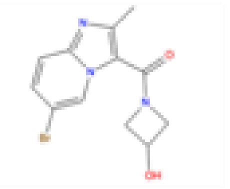	**TMPRSS2**	-7.004282	310.15	4.035	492.17	175.934	111.708	127.091	77.438	823.474	1	5.2	1.975	-3.325	-3.798	-4.35	864.122	1122.014	-2.843	-0.236	58.36	0	0
